# Supplementary material for: Amplicon-Based Microbiome Profiling: From Second- to Third-Generation Sequencing for Higher Taxonomic Resolution
Source: Genes (Basel). 2023 Jul 31;14(8):1567. doi: 10.3390/genes14081567 (PMC10454624; doi:10.3390/genes14081567)

**Supplementary Figure S1: Correlation between the expected and observed relative abundances (%) of reads mapping on 16S rRNA genes of the mock species.** (a) Correlation for the V3V4 region; (b) correlation for the V5V6 region; (c) correlation for the V4 region; (d) correlation for the multiplex approach; (e) correlation for preliminary ASVs of the multiplex approach; (f) correlation for the full-length approach. For each method, the adjusted R2, linear model coefficient (coef.), Pearson correlation coefficient (r) and p-value are shown.

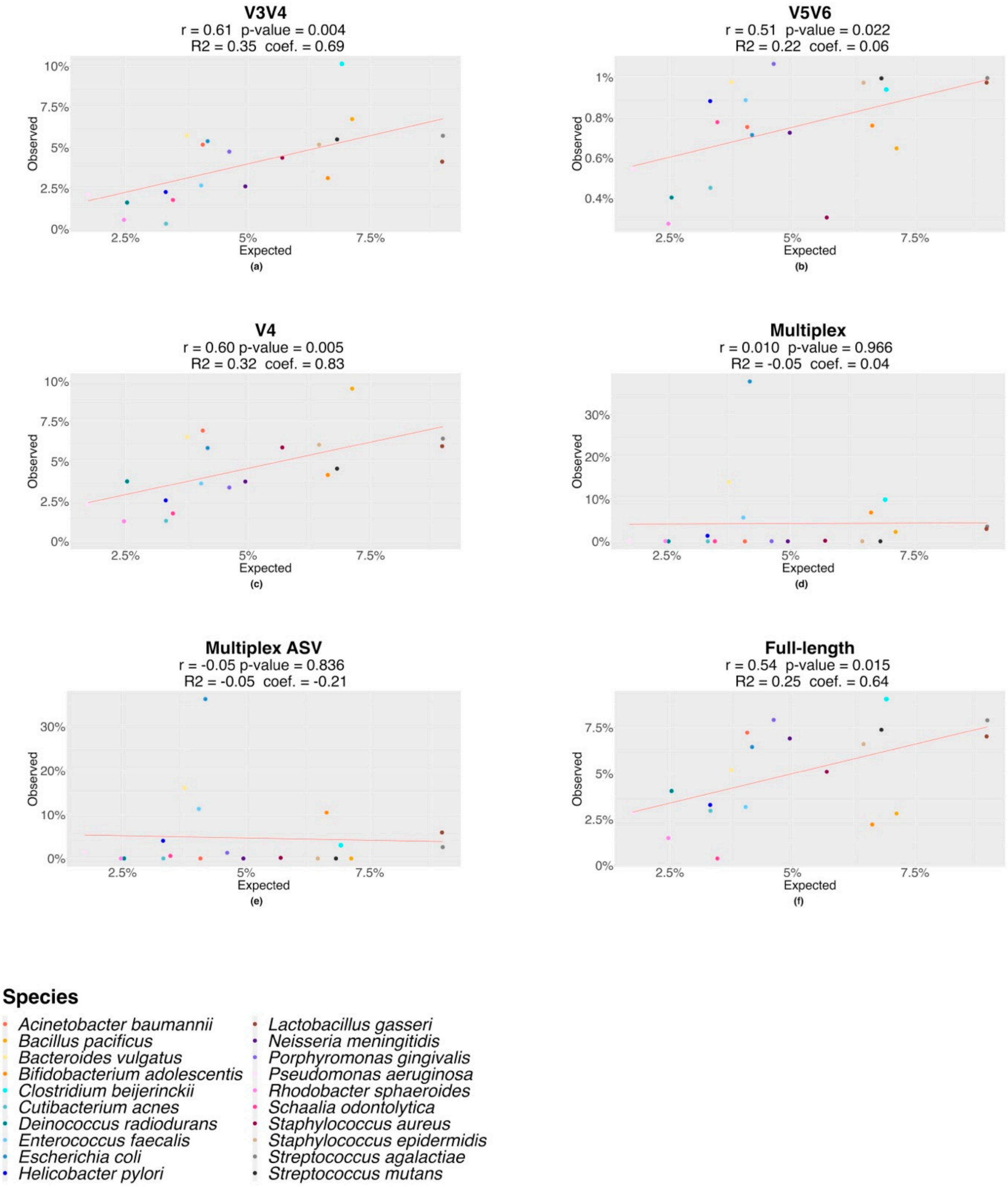

Supplement: Supplementary file 1 [file genes-14-01567-s001.zip › Supplementary Figure S1_ok.pdf]
