# Supplementary material for: Amplicon-Based Microbiome Profiling: From Second- to Third-Generation Sequencing for Higher Taxonomic Resolution
Source: Genes (Basel). 2023 Jul 31;14(8):1567. doi: 10.3390/genes14081567 (PMC10454624; doi:10.3390/genes14081567)

Supplementary Figure S2: The observed 16S rRNA gene coverage by the multiplex approach. The figure shows the different coverage of the 16S rRNA gene produced by multiplex raw reads ("Multiplex") and pASVs ("Multiplex ASV"). Only the species identified by both multiplex raw reads (18/20) and pASVs (12/20) are shown.

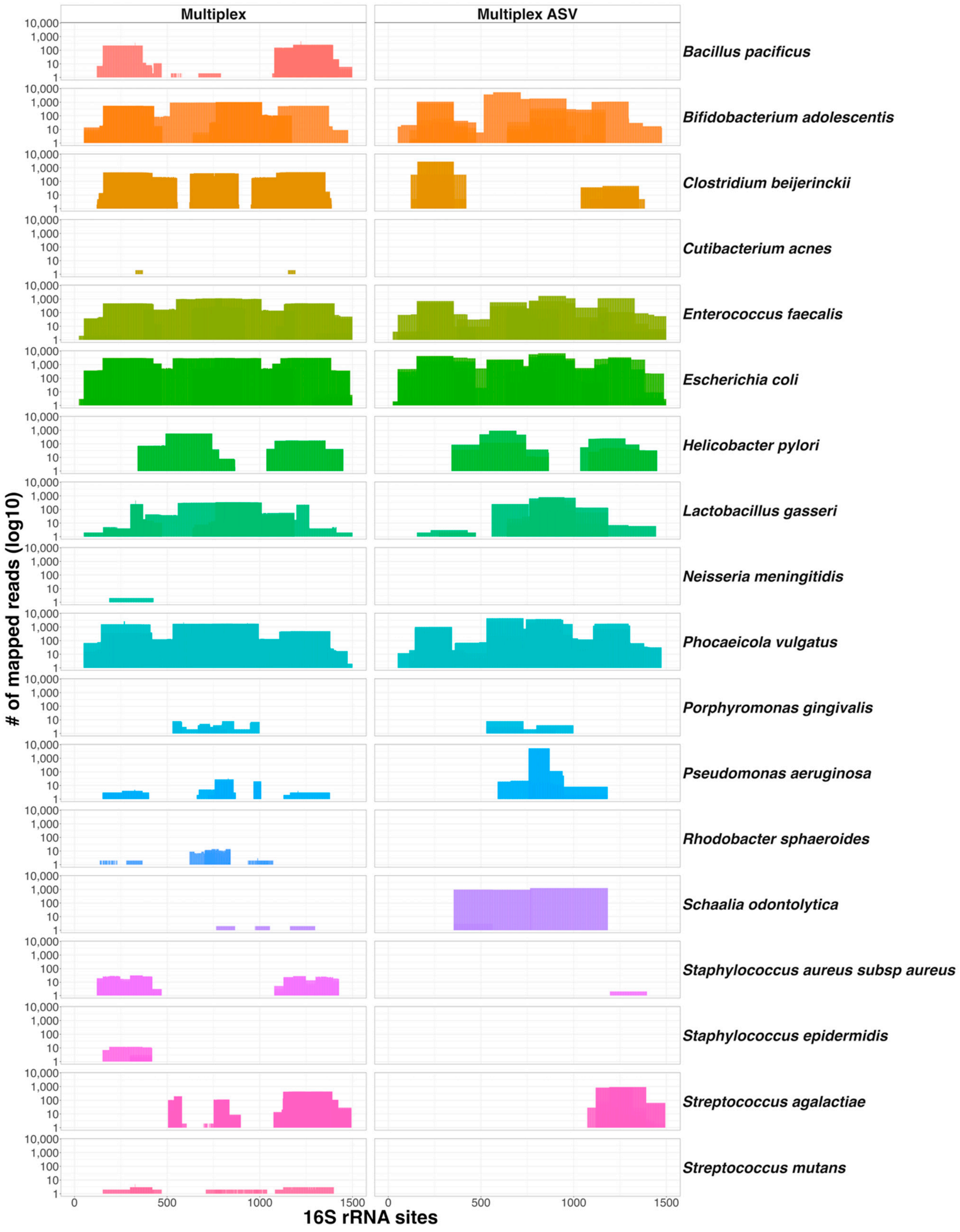

Supplement: Supplementary file 1 [file genes-14-01567-s001.zip › Supplementary Figure S2_ok.pdf]
