# Supplementary material for: Amplicon-Based Microbiome Profiling: From Second- to Third-Generation Sequencing for Higher Taxonomic Resolution
Source: Genes (Basel). 2023 Jul 31;14(8):1567. doi: 10.3390/genes14081567 (PMC10454624; doi:10.3390/genes14081567)
Supplement: Supplementary file 1 [file genes-14-01567-s001.zip › Supplementary Figure S3.pdf]

Supplementary Figure S3: ROC curves and AUC scores. The Receiver Operating Characteristics Curves and the Area Under The Curve scores generated considering the true positive (ASVs that map to the relevant reference genomes and were correctly classified) and false positive (ASVs which map on genomic region annotated for 16S rRNA genes and were not correctly classified) rate for the tested sequencing methods: Single-region (V3V4, V5V6 and V4 region), Multiplex and Full-length (FL). (a) ROC Curves at Genus level; (b) ROC Curves at Species level.

.

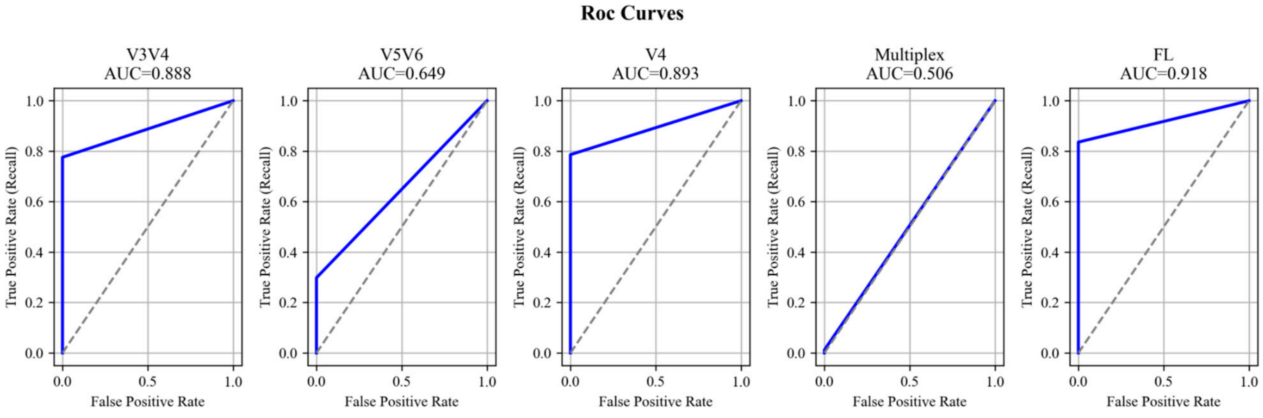

(a)

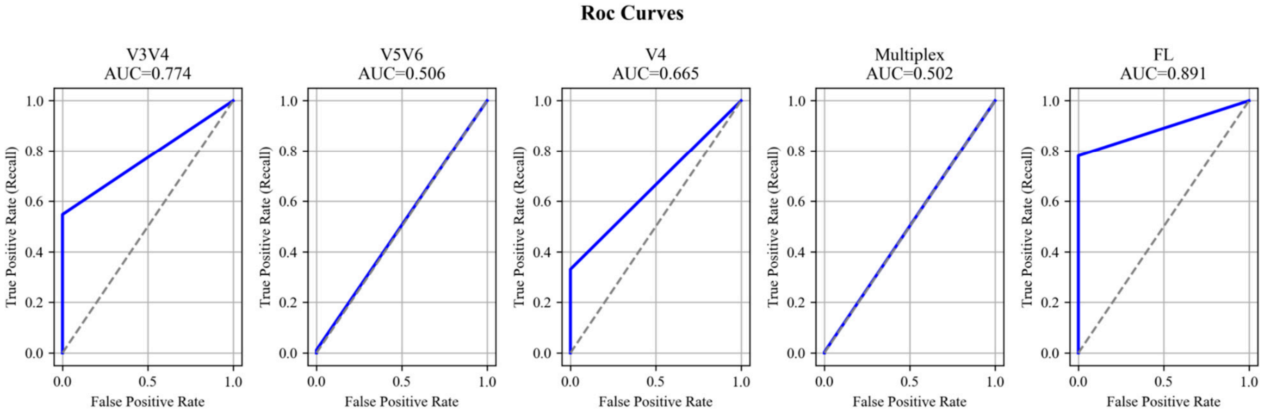

(b)
