# Supplementary material for: Amplicon-Based Microbiome Profiling: From Second- to Third-Generation Sequencing for Higher Taxonomic Resolution
Source: Genes (Basel). 2023 Jul 31;14(8):1567. doi: 10.3390/genes14081567 (PMC10454624; doi:10.3390/genes14081567)
Supplement: Supplementary file 1 [file genes-14-01567-s001.zip › Supplementary Table S1.pdf]

Supplementary Table S1: Taxonomic composition and genomic characteristics of the Mock ATCC 20 Strain Even Mix (MSA-1002TM, ATCC®, USA). Per each strain, the species and the genus scientific name, the number of 16S rRNA gene copies and the genome size are listed. Complete genome and annotation are available at ATCC genome browser (https://www.atcc.org/products/msa-1002).

**Phylum**	**Species**	**16S copies**	**Genome (bp)**
Proteobacteria	*Acinetobacter baumannii*	6	4,075,779
Firmicutes	*Bacillus pacificus*	14	5,442,819
Bacteroidota	*Bacteroides vulgatus*	7	5,163,177
Actinobacteriota	*Bifidobacterium adolescentis*	5	2,089,630
Firmicutes	*Clostridium beijerinckii*	15	6,007,460
Actinobacteriota	*Cutibacterium acnes*	3	2,497,484
Deinococcota	*Deinococcus radiodurans*	3	3,280,465
Firmicutes	*Enterococcus faecalis*	4	2,738,556
Proteobacteria	*Escherichia coli*	7	4,642,497
Proteobacteria	*Helicobacter pylori*	2	1,667,794
Firmicutes	*Lactobacillus gasseri*	6	1,854,273
Proteobacteria	*Neisseria meningitidis*	4	2,240,102
Bacteroidota	*Porphyromonas gingivalis*	4	2,399,479
Proteobacteria	*Pseudomonas aeruginosa*	4	6,374,461
Proteobacteria	*Rhodobacter sphaeroides*	4	4,488,518
Actinobacteriota	*Schaalia odontolytica*	3	2,396,044
Firmicutes	*Staphylococcus aureus*	6	2,915,427
Firmicutes	*Staphylococcus epidermidis*	6	2,575,951
Firmicutes	*Streptococcus agalactiae*	7	2,159,783
Firmicutes	*Streptococcus mutans*	5	2,031,444
