# Supplementary material for: Amplicon-Based Microbiome Profiling: From Second- to Third-Generation Sequencing for Higher Taxonomic Resolution
Source: Genes (Basel). 2023 Jul 31;14(8):1567. doi: 10.3390/genes14081567 (PMC10454624; doi:10.3390/genes14081567)
Supplement: Supplementary file 1 [file genes-14-01567-s001.zip › Supplementary Table S2.pdf]

Supplementary Table S2: The expected and observed 16S rRNA abundances (%) for each sequencing approach at the genus and species level.

	Expected (%)	Observed (%)				
		V3V4	V5V6	V4	Multiplex	Full-length
**Genus**						
Acinetobacter	4.07	6.76	11.46	7.19	0.00	7.60
Bacillus	7.12	7.60	0.95	9.94	0.00	1.97
Bacteroides	3.75	7.62	0.00	6.66	0.34	5.79
Bifidobacterium	6.62	4.12	1.73	4.17	0.21	2.18
Clostridium	6.91	13.2	37.6	13.8	7	9.42
Cutibacterium	3.33	0.43	0.00	1.31	0.00	3.08
Deinococcus	2.53	2.06	0.00	3.86	0.00	4.52
Enterococcus	4.04	2.90	0.88	3.58	0.21	2.38
Escherichia-Shigella	4.17	6.90	0.63	5.76	0.39	6.60
Helicobacter	3.32	3.12	0.00	2.52	0.04	3.62
Lactobacillus	8.96	5.09	0.00	5.74	0.20	6.77
Neisseria	4.94	3.50	0.00	3.85	0.00	7.37
Porphyromonas	4.62	6.39	0.00	3.39	0.00	9.41
Pseudomonas	1.74	2.72	0.30	2.38	0.03	2.72
Rhodobacter	2.47	0.77	0.00	1.30	0.00	1.57
Schaalia	3.47	2.30	0.2	1.75	0.00	0.36
Staphylococcus	12.15	11.23	13.58	12.31	0.004	10.21
Streptococcus	15.79	13.17	1.30	10.07	0.05	14.31
**Species**						
Acinetobacter baumannii	4.08	6.75	0.00	0.00	0.00	7.60
Bacillus pacificus	7.12	0.00	0.00	0.00	0.00	0.00
Bacteroides vulgatus	3.75	7.60	0.00	6.66	0.002	5.79
Bifidobacterium adolescentis	6.62	3.83	0.00	0.00	0.02	2.18
Clostridium beijerinckii	6.91	0.00	0.00	0.83	0.00	9.42

	Expected (%)	Observed (%)				
		**V3V4**	**V5V6**	**V4**	**Multiplex**	**Full-length**
Cutibacterium acnes	3.33	0.00	0.00	0.00	0.00	0.00
Deinococcus radiodurans	2.53	2.05	0.00	3.86	0.00	4.52
Enterococcus faecalis	4.04	0.00	0.18	0.00	0.06	2.38
Escherichia coli	4.17	0.00	0.62	0.00	0.00	0.00
Helicobacter pylori	3.32	3.11	0.00	2.52	0.004	3.62
Lactobacillus gasseri	8.96	5.00	0.00	0.00	0.00	6.77
Neisseria meningitidis	4.94	3.50	0.00	3.85	0.00	7.37
Porphyromonas gingivalis	4.62	6.39	0.00	3.39	0.00	9.41
Pseudomonas aeruginosa	1.74	0.00	0.30	0.00	0.00	2.72
Rhodobacter sphaeroides	2.47	0.77	0.00	0.00	0.00	1.57
Schaalia odontolytica	3.47	2.27	0.00	1.75	0.00	0.36
Staphylococcus aureus	5.70	0.00	0.00	0.00	0.00	0.00
Staphylococcus epidermidis	6.45	0.00	0.02	0.00	0.00	0.00
Streptococcus agalactiae	8.97	6.84	0.00	5.55	0.00	7.24
Streptococcus mutans	6.81	6.29	0.00	4.52	0.00	7.07
