# Supplementary material for: Amplicon-Based Microbiome Profiling: From Second- to Third-Generation Sequencing for Higher Taxonomic Resolution
Source: Genes (Basel). 2023 Jul 31;14(8):1567. doi: 10.3390/genes14081567 (PMC10454624; doi:10.3390/genes14081567)
Supplement: Supplementary file 1 [file genes-14-01567-s001.zip › Supplementary Table S3.pdf]

Supplementary Table S3: Summary of raw reads mapping on the mock species reference genomes. The table shows the rate of PE reads mapping on the same 16S rRNA genes or on identical copies ("On target"), PE reads mapped in genomic region out of those annotate to contain 16S rRNA genes ("Out target"), PE reads mapping on different non identical copies of the 16S rRNA genes considered ambiguous ("On target low qual") and unmapped reads ("unmapped").

Methods	On target	Out target	On target low qual	unmapped
**V3V4**	79.81%	0.001%	20.11%	0.07%
**V4**	98.76%	0.03%	0.89%	0.32%
**V5V6**	15.04%	0.00%	78.40%	6.57%
**Full-length**	99.98%	0.00%	0.00%	0.02%
**Multiplex**	84.62%	0.00%	2.23%	13.14%
