# Supplementary material for: Amplicon-Based Microbiome Profiling: From Second- to Third-Generation Sequencing for Higher Taxonomic Resolution
Source: Genes (Basel). 2023 Jul 31;14(8):1567. doi: 10.3390/genes14081567 (PMC10454624; doi:10.3390/genes14081567)
Supplement: Supplementary file 1 [file genes-14-01567-s001.zip › Supplementary Table S4.pdf]

Supplementary Table S4: The expected and observed relative abundances (%) of reads mapping on 16S rRNA genes per each species of the mock community. The table exposes the expected and observed relative abundances of reads mapping to the 16S rRNA genes per each species, generated from the different sequencing approaches. Additionally, the relative abundances of preliminary ASVs, derived from the specific Swift bioinformatic workflow, are shown ("Multiplex ASV").

	**Expected (%)**	**Observed (%)**					
		**V3V4**	**V5V6**	**V4**	**Multiplex**	**Full-length**	**Multiplex ASV**
**Species**							
Acinetobacter baumannii	4.08	5.17	0.75	6.93	0.0	7.22	0.0
Bacillus pacificus	7.12	6.73	0.65	9.55	2.23	2.83	0.0
Bacteroides vulgatus	3.75	5.73	0.98	6.53	14.05	5.19	16.09
Bifidobacterium adolescentis	6.62	3.12	0.76	4.15	6.82	2.21	10.49
Clostridium beijerinckii	6.91	10.13	0.94	13.27	9.89	9.04	3.02
Cutibacterium acnes	3.33	0.32	0.45	1.28	0.004	2.99	0.0
Deinococcus radiodurans	2.53	1.61	0.40	3.75	0.0	4.06	0.0
Enterococcus faecalis	4.04	2.66	0.89	3.62	5.64	3.19	11.35
Escherichia coli	4.17	5.38	0.71	5.83	37.88	6.44	36.21
Helicobacter pylori	3.32	2.27	0.88	2.56	1.31	3.30	4.02
Lactobacillus gasseri	8.96	4.12	0.97	5.95	2.93	7.01	5.89
Neisseria meningitidis	4.94	2.61	0.72	3.74	0.005	6.90	0.0
Porphyromonas gingivalis	4.62	4.74	1.07	3.36	0.03	7.91	1.29
Pseudomonas aeruginosa	1.74	2.11	0.54	2.32	0.07	2.88	1.44
Rhodobacter sphaeroides	2.47	0.56	0.27	1.26	0.05	1.48	0.0
Schaalia odontolytica	3.47	1.78	0.78	1.75	0.02	0.37	0.57
Staphylococcus aureus	5.70	4.36	0.30	5.87	0.15	5.10	0.144
Staphylococcus epidermidis	6.45	5.17	0.97	6.04	0.02	6.60	0.0
Streptococcus agalactiae	8.97	5.72	0.99	6.43	3.51	7.89	2.59
Streptococcus mutans	6.81	5.49	0.99	4.55	0.01	7.37	0.0
