# Supplementary material for: Amplicon-Based Microbiome Profiling: From Second- to Third-Generation Sequencing for Higher Taxonomic Resolution
Source: Genes (Basel). 2023 Jul 31;14(8):1567. doi: 10.3390/genes14081567 (PMC10454624; doi:10.3390/genes14081567)
Supplement: Supplementary file 1 [file genes-14-01567-s001.zip › Supplementary Table S5.pdf]

Supplementary Table S5. ASVs mapping to the reference genomes and the corresponding absolute and relative abundances. Parameters considered: query coverage ≥90% and similarity ≥97%. "Genome" represents the contig where the ASVs align and "sstart" / "send" the location coordinates; "pident" is the % of similarity. "Method" refers to the sequencing approach. "Genome Name" is the name of the species of the mock. whereas "Assigned Species" and "Assigned Genus" correspond to the classification obtained at species and genus level. respectively. "Expected" shows whether the region of the match is annotated as the 16S rRNA gene.

ASV	**Genome**	**pident**	**sstart**	**send**	**Method**	**Genome Name**	**Expected**	**Assigned Species**	**Assigned Genus**	**abs**	**rel**
116988315318af814daac19fb60efb18					V3V4				d__Bacteria;p__Firmicutes;c__Bacilli;o__Lactobacillales;f__Streptococcaceae;g__Streptococcus	2	0.00
ddd048fa84342d57fc9206683593998c					V3V4				d__Bacteria;p__Firmicutes;c__Bacilli;o__Staphylococcales;f__Staphylococcaceae;g__Staphylococcus	1.00E+00	0.00
0af0987236663cf1a404ee8f8d0d9ef4					V3V4				d__Bacteria;p__Bacteroidota;c__Bacteroidia;o__Bacteroidales;f__Bacteroidaceae;g__Bacteroides	4	0.00
c84fc45ac0df1b36f969d4b7d4b4d76a					V3V4				d__Bacteria;p__Actinobacteriota;c__Actinobacteria;o__Bifidobacteriales;f__Bifidobacteriaceae;g__Bifidobacterium	1	0.00
2fd84e313a8e8a45246db82a092a03c1					V3V4			d__Bacteria;p__Proteobacteria;c__Gammaproteobacteria;o__Enterobacterales;f__Enterobacteriaceae;g__Escherichia-Shigella; s__uncultured_Pantoea	d__Bacteria;p__Proteobacteria;c__Gammaproteobacteria;o__Enterobacterales;f__Enterobacteriaceae;g__Escherichia-Shigella	1	0.00
9d3843fd30f6e5882ad76d87bf912be1					V3V4			d__Bacteria;p__Actinobacteriota;c__Actinobacteria;o__Bifidobacteriales;f__Bifidobacteriaceae;g__Bifidobacterium;s__Bifidobacterium_faecale	d__Bacteria;p__Actinobacteriota;c__Actinobacteria;o__Bifidobacteriales;f__Bifidobacteriaceae;g__Bifidobacterium	2	0.00
80b9934b6e140e0309d745b33db8da19					V3V4				d__Bacteria;p__Firmicutes;c__Bacilli;o__Lactobacillales;f__Enterococcaceae;g__Enterococcus	3	0.00
b347624d5c5225ccf4cd38d7a0dcc598					V3V4				d__Bacteria;p__Proteobacteria;c__Gammaproteobacteria;o__Enterobacterales;f__Enterobacteriaceae;g__Escherichia-Shigella	2	0.00
f9aa8d0e7ab74dd5b1c80f4e823b6886					V3V4				d__Bacteria;p__Actinobacteriota;c__Actinobacteria;o__Bifidobacteriales;f	4	0.00

			__Bifidobacteriaceae;g__Bifidobacterium		
456726fcb18558bdbc6377e38896547e	V3V4		d__Bacteria;p__Proteobacteria;c__Gammaproteobacteria;o__Pseudomonadales;f__Pseudomonadaceae;g__Pseudomonas	19	0.02
6a0eae87b93fd07ad46db3b1eaab2d43	V3V4		d__Bacteria;p__Firmicutes;c__Bacilli;o__Lactobacillales;f__Lactobacillaceae;g__Lactobacillus	2	0.00
1c98130024b2622235bedbaf30c690d9	V3V4		d__Bacteria;p__Bacteroidota;c__Bacteroidia;o__Bacteroidales;f__Bacteroidaceae;g__Bacteroides	1.00E+00	0.00
e22ead36335328e214f9b66dfb1b30d1	V3V4		d__Bacteria;p__Proteobacteria;c__Gammaproteobacteria;o__Pseudomonadales;f__Pseudomonadaceae;g__Pseudomonas	3	0.00
ac2d45456f92a5bc851ac12762130a96	V3V4			5	0.01
1c02778d589eec0fecf00d976b50fec8	V3V4		d__Bacteria;p__Campilobacterota;c__Campylobacteria;o__Campylobacterales;f__Helicobacteraceae;g__Helicobacter	2	0.00
ab86e2b48526034e6f335615a043d483	V3V4		d__Bacteria;p__Firmicutes;c__Bacilli;o__Lactobacillales;f__Lactobacillaceae;g__Lactobacillus	1	0.00
b0a2169e2a052ead8185d4ac9668ede5	V3V4		d__Bacteria;p__Firmicutes;c__Bacilli;o__Lactobacillales;f__Lactobacillaceae;g__Lactobacillus	2	0.00
245ba600e28a95b9df533245cb5a222d	V3V4	d__Bacteria;p__Firmicutes;c__Bacilli;o__Lactobacillales;f__Lactobacillaceae;g__Lactobacillus;s__uncultured_bacterium	d__Bacteria;p__Firmicutes;c__Bacilli;o__Lactobacillales;f__Lactobacillaceae;g__Lactobacillus	25	0.03
4dbd2311865e718e629475768ec56594	V3V4			4	0.00
5a92b2a407a87bb5d987cec082ef10db	V3V4		d__Bacteria;p__Proteobacteria;c__Gammaproteobacteria;o__Pseudomonadales;f__Pseudomonadaceae;g__Pseudomonas	4	0.00
ae8c26f926b77ff67bb2949dbcf67be3	V3V4		d__Bacteria;p__Actinobacteriota;c__Actinobacteria;o__Bifidobacteriales;f__Bifidobacteriaceae;g__Bifidobacterium	1	0.00

3871f21058d3b19c17520843acb6e109	V3V4	d__Bacteria;p__Proteobacteria;c__Gammaproteobacteria;o__Pseudomonadales;f__Pseudomonadaceae;g__Pseudomonas;s__uncultured_Marinobacter	d__Bacteria;p__Proteobacteria;c__Gammaproteobacteria;o__Pseudomonadales;f__Pseudomonadaceae;g__Pseudomonas	2	0.00
e9970500442b3168e1d6522bb65e33f3	V3V4		d__Bacteria;p__Actinobacteriota;c__Actinobacteria;o__Actinomycetales;f__Actinomycetaceae;g__Actinomyces	3	0.00
b154827d580ab44bf2c451c4f9e74a60	V3V4		d__Bacteria;p__Firmicutes;c__Bacilli;o__Lactobacillales;f__Enterococcaceae;g__Enterococcus	3	0.00
052f8d7804cf053a144f4474566d4fd0	V3V4	d__Bacteria;p__Firmicutes;c__Bacilli;o__Lactobacillales;f__Streptococcaceae;g__Streptococcus;s__Streptococcus_troglodytae	d__Bacteria;p__Firmicutes;c__Bacilli;o__Lactobacillales;f__Streptococcaceae;g__Streptococcus	5	0.01
d86828b11a697d3aee5c4d466b988380	V3V4	d__Bacteria;p__Firmicutes;c__Bacilli;o__Lactobacillales;f__Lactobacillaceae;g__Lactobacillus;s__uncultured_bacterium	d__Bacteria;p__Firmicutes;c__Bacilli;o__Lactobacillales;f__Lactobacillaceae;g__Lactobacillus	18	0.02
2f11acb78d09ec9436a120ef712836de	V3V4		d__Bacteria;p__Firmicutes;c__Bacilli;o__Staphylococcales;f__Staphylococcaceae;g__Staphylococcus	3	0.00
80dbafcfb4b1f684d52673147ac8d308	V3V4	d__Bacteria;p__Bacteroidota;c__Bacteroidia;o__Bacteroidales;f__Bacteroidaceae;g__Bacteroides;s__Bacteroides_thetaiotaomicron	d__Bacteria;p__Bacteroidota;c__Bacteroidia;o__Bacteroidales;f__Bacteroidaceae;g__Bacteroides	5	0.01
901163b6f627002d8cbc1afae9f4f405	V3V4	d__Bacteria;p__Firmicutes;c__Bacilli;o__Lactobacillales;f__Lactobacillaceae;g__Lactobacillus;s__uncultured_bacterium	d__Bacteria;p__Firmicutes;c__Bacilli;o__Lactobacillales;f__Lactobacillaceae;g__Lactobacillus	1	0.00
2c5c63739b3f140a688647b359e290c1	V3V4	d__Bacteria;p__Firmicutes;c__Bacilli;o__Lactobacillales;f__Streptococcaceae;g__Streptococcus;s__Streptococcus_troglodytae	d__Bacteria;p__Firmicutes;c__Bacilli;o__Lactobacillales;f__Streptococcaceae;g__Streptococcus	1	0.00
7ffac11a381face986d02ca535d4acb4	V3V4		d__Bacteria;p__Actinobacteriota;c__Actinobacteria;o__Actinomycetales;f__Actinomycetaceae;g__Actinomyces	1	0.00
063506beba6c1fbb0929c8d0e8a3ff81	V3V4	d__Bacteria;p__Actinobacteriota;c__Actinobacteria;o__Actinomycetales;f__Actinomycetaceae;g__Actinomyces;s__Mycobacterium_tuberculosis	d__Bacteria;p__Actinobacteriota;c__Actinobacteria;o__Actinomycetales;f__Actinomycetaceae;g__Actinomyces	1	0.00

66fc81d0779ee3543c336e55f997ff18	V3V4		d__Bacteria;p__Proteobacteria;c__Gammaproteobacteria;o__Pseudomonadales;f__Moraxellaceae;g__Acinetobacter	1	0.00
33bc5391ced32708caab9eeee2f6d17a	V3V4	d__Bacteria;p__Firmicutes;c__Bacilli;o__Lactobacillales;f__Streptococcaceae;g__Streptococcus;s__Streptococcus_troglodytae	d__Bacteria;p__Firmicutes;c__Bacilli;o__Lactobacillales;f__Streptococcaceae;g__Streptococcus	7	0.01
e8119a79e6d3fd00d40d024ec7db9280	V3V4		d__Bacteria;p__Firmicutes;c__Bacilli;o__Staphylococcales;f__Staphylococcaceae;g__Staphylococcus	1	0.00
847571fab7d7e46326f4d957421d55f4	V3V4		d__Bacteria;p__Firmicutes;c__Bacilli;o__Lactobacillales;f__Lactobacillaceae;g__Lactobacillus	2	0.00
e61cd640ab153c0aa0eb50ef858c4762	V3V4		d__Bacteria;p__Deinococcota;c__Deinococci;o__Deinococcales;f__Deinococcaceae;g__Deinococcus	2	0.00
3ca103332462a6f22bc3f3a71e192861	V3V4			4	0.00
c66358457390ce993711d5e370a5f286	V3V4		d__Bacteria;p__Firmicutes;c__Bacilli;o__Lactobacillales;f__Lactobacillaceae;g__Lactobacillus	1	0.00
6076e162d2870cad4ff4cccf66bea562	V3V4			1	0.00
07a15062be8ee30ddc3329c1e78768dc	V3V4		d__Bacteria;p__Firmicutes;c__Bacilli;o__Lactobacillales;f__Enterococcaceae;g__Enterococcus	4.00E+00	0.00
edd5425137c3ff776e8e9bc317afb58b	V3V4	d__Bacteria;p__Firmicutes;c__Bacilli;o__Lactobacillales;f__Streptococcaceae;g__Streptococcus;s__Streptococcus_troglodytae	d__Bacteria;p__Firmicutes;c__Bacilli;o__Lactobacillales;f__Streptococcaceae;g__Streptococcus	4	0.00
905130d5a231d43581bea3477d953f09	V3V4		d__Bacteria;p__Firmicutes;c__Bacilli;o__Lactobacillales;f__Lactobacillaceae;g__Lactobacillus	1	0.00
b527ff38a17f5fdb76c2636c890e15ce	V3V4		d__Bacteria;p__Firmicutes;c__Clostridia;o__Clostridiales;f__Clostridiaceae;g__Clostridium_sensu_stricto_1	1	0.00
b0cc6e3b244191ab1357fe19e1fc9a2e	V3V4		d__Bacteria;p__Firmicutes;c__Bacilli;o__Staphylococcales;f__Staphylococcaceae;g__Staphylococcus	1	0.00

492ed26e8fe4359d9dd1c133c3e7b3cb	V3V4			1	0.00
cb93c047af2ba2e33389d8b998a28150	V3V4		d__Bacteria;p__Actinobacteriota;c__Actinobacteria;o__Actinomycetales;f__Actinomycetaceae;g__Actinomyces	10	0.01
5283f476090a0248b7f6fffc1348a8e8	V3V4		d__Bacteria;p__Actinobacteriota;c__Actinobacteria;o__Bifidobacteriales;f__Bifidobacteriaceae;g__Bifidobacterium	40	0.04
90a6a6f14d4d80c00fa17c150ca9ebce	V3V4	d__Bacteria;p__Firmicutes;c__Bacilli;o__Lactobacillales;f__Lactobacillaceae;g__Lactobacillus;s__mouse_gut	d__Bacteria;p__Firmicutes;c__Bacilli;o__Lactobacillales;f__Lactobacillaceae;g__Lactobacillus	2	0.00
fa7f3fe11e36c4b6d30ae148713ff05f	V3V4		d__Bacteria;p__Firmicutes;c__Clostridia;o__Peptostreptococcales-Tissierellales;f__Peptostreptococcaceae;g__Romboutsia	9	0.01
f74124cf2698857f11924bc4cc47b6c2	V3V4	d__Bacteria;p__Proteobacteria;c__Gammaproteobacteria;o__Enterobacterales;f__Enterobacteriaceae;g__Escherichia-Shigella;s__uncultured_Escherichia	d__Bacteria;p__Proteobacteria;c__Gammaproteobacteria;o__Enterobacterales;f__Enterobacteriaceae;g__Escherichia-Shigella	9	0.01
44163af77873c2c3b907dd79b05e2f0f	V3V4		d__Bacteria;p__Proteobacteria;c__Gammaproteobacteria;o__Pseudomonadales;f__Pseudomonadaceae;g__Pseudomonas	10	0.01
397d12199a2dee22c6c3cb0607d4b244	V3V4		d__Bacteria;p__Firmicutes;c__Bacilli;o__Lactobacillales;f__Lactobacillaceae;g__Lactobacillus	1	0.00
ee63c66fa9845ff018f2385400ffac1c	V3V4	d__Bacteria;p__Firmicutes;c__Bacilli;o__Lactobacillales;f__Streptococcaceae;g__Streptococcus;s__Streptococcus_troglodytae	d__Bacteria;p__Firmicutes;c__Bacilli;o__Lactobacillales;f__Streptococcaceae;g__Streptococcus	2	0.00
4498526ac722cce4d07cae5cbaca1224	V3V4		d__Bacteria;p__Firmicutes;c__Bacilli;o__Lactobacillales;f__Enterococcaceae;g__Enterococcus	2	0.00
30552d6e4905472fe90f1d1ea2584c57	V3V4		d__Bacteria;p__Firmicutes;c__Bacilli;o__Lactobacillales;f__Streptococcaceae;g__Streptococcus	4	0.00
2e081b7ff660c34d0503c723de0faab3	V3V4		d__Bacteria;p__Proteobacteria;c__Gammaproteobacteria;o__Pseudomonadales;f__Moraxellaceae;g__Acinetobacter	3	0.00

abce05ed9ee1d389232f2a3ea3bc9657	V3V4		d__Bacteria;p__Firmicutes;c__Clostridia;o__Clostridiales;f__Clostridiaceae;g__Clostridium_sensu_stricto_1	2	0.00
7be95baaddca163f1c6b316ba86219ee	V3V4		d__Bacteria;p__Proteobacteria;c__Gammaproteobacteria;o__Enterobacterales;f__Enterobacteriaceae;g__Escherichia-Shigella	2	0.00
7dd0c4a5fa63118d9da0fe733dbe7f5c	V3V4		d__Bacteria;p__Firmicutes;c__Bacilli;o__Lactobacillales;f__Lactobacillaceae;g__Lactobacillus	1	0.00
3f012a293d1c7f7075486004465ca156	V3V4		d__Bacteria;p__Firmicutes;c__Bacilli;o__Lactobacillales;f__Lactobacillaceae;g__Lactobacillus	1	0.00
b348310b9ad5413880fcab00d56e2494	V3V4	d__Bacteria;p__Firmicutes;c__Bacilli;o__Lactobacillales;f__Lactobacillaceae;g__Lactobacillus;s__mouse_gut	d__Bacteria;p__Firmicutes;c__Bacilli;o__Lactobacillales;f__Lactobacillaceae;g__Lactobacillus	2	0.00
c92d34b04f93ee76340cf1dfd390cad3	V3V4		d__Bacteria;p__Firmicutes;c__Bacilli;o__Lactobacillales;f__Lactobacillaceae;g__Lactobacillus	2	0.00
8176a59241125253f19efb1a2a1025a7	V3V4		d__Bacteria;p__Firmicutes;c__Clostridia;o__Clostridiales;f__Clostridiaceae;g__Clostridium_sensu_stricto_1	1	0.00
f32c3098ccb00b274be15992dbf7c1e3	V3V4			1	0.00
1cfaf7686133c22ec3308efb96e0a22a	V3V4			2	0.00
3829fc0b69d79439dc17485f0ec03fff	V3V4		d__Bacteria;p__Bacteroidota;c__Bacteroidia;o__Bacteroidales;f__Bacteroidaceae;g__Bacteroides	1	0.00
5c666123cdf2417dcff3c0ce45e89436	V3V4		d__Bacteria;p__Proteobacteria;c__Gammaproteobacteria;o__Burkholderiales;f__Neisseriaceae;g__Neisseria	1	0.00
381b667dc5dd68c1fbe51b785562c860	V3V4		d__Bacteria;p__Bacteroidota;c__Bacteroidia;o__Bacteroidales;f__Bacteroidaceae;g__Bacteroides	5	0.01
e7988e34895ba1648612e0e677fc9e4e	V3V4			1	0.00
0e157a51af08007fc87a7c2925c674f1	V3V4	d__Bacteria;p__Proteobacteria;c__Gammaproteobacteria;o__Enterobacterales;f__Enterobacteriaceae;g__Escher	d__Bacteria;p__Proteobacteria;c__Gammaproteobacteria;o__Enterobacter	1	0.00

		ichia-Shigella;s__uncultured_Pantoea	ales;f__Enterobacteriaceae;g__Escherichia-Shigella		
76444b43bd86d5ee5f46904d49c9754d	V3V4		d__Bacteria;p__Actinobacteriota;c__Actinobacteria;o__Bifidobacteriales;f__Bifidobacteriaceae;g__Bifidobacterium	19	0.02
78964bebb9fd2e86a40c9ed3585969a6	V3V4	d__Bacteria;p__Firmicutes;c__Bacilli;o__Lactobacillales;f__Lactobacillaceae;g__Lactobacillus;s__uncultured_bacterium	d__Bacteria;p__Firmicutes;c__Bacilli;o__Lactobacillales;f__Lactobacillaceae;g__Lactobacillus	1	0.00
e07ea05065633374ef3159033952de15	V3V4			2	0.00
c1a575ede20550366883decab3d0b7e1	V3V4			16	0.02
5848a447494c6f23aa875baa5de5a8da	V3V4		d__Bacteria;p__Firmicutes;c__Clostridia;o__Clostridiales;f__Clostridiaceae;g__Clostridium_sensu_stricto_1	2	0.00
1d927a61fda6a288d40c2479c1da7b53	V3V4		d__Bacteria;p__Proteobacteria;c__Gammaproteobacteria;o__Enterobacterales;f__Enterobacteriaceae;g__Escherichia-Shigella	3	0.00
699e0f7469c165b701834385f9a13ed7	V3V4		d__Bacteria;p__Firmicutes;c__Bacilli;o__Staphylococcales;f__Staphylococcaceae;g__Staphylococcus	1	0.00
b468694797c5990818fe1cf509f2f807	V3V4		d__Bacteria;p__Proteobacteria;c__Gammaproteobacteria;o__Pseudomonadales;f__Pseudomonadaceae;g__Pseudomonas	4	0.00
08bc05c4756c28aca9d5e9f1dc5901ad	V3V4			1	0.00
04a6e1bfd2e94a99f1bd4c7f4f5b7017	V3V4			3	0.00
4c7b75d43255eed2661601ed23c87123	V3V4			3	0.00
b96951bab7f5282a3531696b0e3ec620	V3V4		d__Bacteria;p__Firmicutes;c__Bacilli;o__Lactobacillales;f__Lactobacillaceae;g__Lactobacillus	1	0.00

278f5855ed26318212ca668522a925c0	V3V4		d__Bacteria;p__Actinobacteriota;c__Actinobacteria;o__Actinomycetales;f__Actinomycetaceae;g__Actinomyces	7	0.01
1070c4298b1ec670ca36faf1e6acca40	V3V4		d__Bacteria;p__Proteobacteria;c__Gammaproteobacteria;o__Burkholderiales;f__Neisseriaceae;g__Neisseria	1	0.00
1f68bc4e3aeeb65d48f63fbef319adae	V3V4		d__Bacteria;p__Bacteroidota;c__Bacteroidia;o__Bacteroidales;f__Bacteroidaceae;g__Bacteroides	1	0.00
41fc41da8fe491d0ab4515cc43afd58e	V3V4		d__Bacteria;p__Proteobacteria;c__Gammaproteobacteria;o__Enterobacterales;f__Enterobacteriaceae;g__Escherichia-Shigella	15	0.02
bd2bc954f821d98b470847b8a59478b0	V3V4		d__Bacteria;p__Firmicutes;c__Bacilli;o__Lactobacillales;f__Lactobacillaceae;g__Lactobacillus	1	0.00
a1d3cc081207900015d39f5b81ecdc35	V3V4	d__Bacteria;p__Firmicutes;c__Bacilli;o__Lactobacillales;f__Streptococcaceae;g__Streptococcus;s__Streptococcus_troglodytae	d__Bacteria;p__Firmicutes;c__Bacilli;o__Lactobacillales;f__Streptococcaceae;g__Streptococcus	1	0.00
7129605bb839e8e5c432cf15aa68c864	V3V4		d__Bacteria;p__Firmicutes;c__Bacilli;o__Lactobacillales;f__Enterococcaceae;g__Enterococcus	1	0.00
b3289bc3fc342223a3882db9a80d88c6	V3V4		d__Bacteria;p__Actinobacteriota;c__Actinobacteria;o__Bifidobacteriales;f__Bifidobacteriaceae;g__Bifidobacterium	14	0.01
fd63fd7ea904a2c74a93909008964afb	V3V4			1	0.00
0aaa2ce281156cedcc1dba12c7fb0b43	V3V4		d__Bacteria;p__Actinobacteriota;c__Actinobacteria;o__Actinomycetales;f__Actinomycetaceae;g__Actinomyces	4	0.00
656a3f9265f027eedeae38b5a655f057	V3V4	d__Bacteria;p__Bacteroidota;c__Bacteroidia;o__Bacteroidales;f__Bacteroidaceae;g__Bacteroides;s__Bacteroides_vulgatus	d__Bacteria;p__Bacteroidota;c__Bacteroidia;o__Bacteroidales;f__Bacteroidaceae;g__Bacteroides	1	0.00
bcc1f1c4c18982e2887807cbe6e1b0fc	V3V4			2	0.00

26055d990cb566310cc60ca222661e9c	V3V4		d__Bacteria;p__Firmicutes;c__Bacilli;o__Lactobacillales;f__Streptococcaceae;g__Streptococcus	1	0.00
8b1242f5d16dcca61bf8d5c4ce68b69c	V3V4		d__Bacteria;p__Firmicutes;c__Bacilli;o__Staphylococcales;f__Staphylococcaceae;g__Staphylococcus	3	0.00
40c09ab1cbda980dbfdbaeff778e1cc1	V3V4	d__Bacteria;p__Firmicutes;c__Clostridia;o__Peptostreptococcales-Tissierellales;f__Anaerovoracaceae;g__Mogibacterium;s__uncultured_bacterium	d__Bacteria;p__Firmicutes;c__Clostridia;o__Peptostreptococcales-Tissierellales;f__Anaerovoracaceae;g__Mogibacterium	4	0.00
a17b3f33f0dd27b289b0f9ef201f53c5	V3V4		d__Bacteria;p__Deinococcota;c__Deinococci;o__Deinococcales;f__Deinococcaceae;g__Deinococcus	2	0.00
68ffc7181ac528d49f7458b4547eb47b	V3V4			5	0.01
f91f431a88c377230dd1c8bb79703853	V3V4		d__Bacteria;p__Proteobacteria;c__Gammaproteobacteria;o__Burkholderiales;f__Neisseriaceae;g__Neisseria	1	0.00
b0060cc6b7ce620870420f8b3fa3b6c1	V3V4		d__Bacteria;p__Proteobacteria;c__Gammaproteobacteria;o__Enterobacterales;f__Enterobacteriaceae;g__Escherichia-Shigella	1	0.00
08dfad997477a5742bc80007f5277ef8	V3V4		d__Bacteria;p__Firmicutes;c__Bacilli;o__Lactobacillales;f__Streptococcaceae;g__Streptococcus	4	0.00
4a8f3208e638a1c148bb51ecbb6ef18d	V3V4			4	0.00
9089af94287bb71026b03ad629aac9a0	V3V4	d__Bacteria;p__Proteobacteria;c__Gammaproteobacteria;o__Pseudomonadales;f__Pseudomonadaceae;g__Pseudomonas;s__uncultured_Marinobacter	d__Bacteria;p__Proteobacteria;c__Gammaproteobacteria;o__Pseudomonadales;f__Pseudomonadaceae;g__Pseudomonas	2	0.00
7d0c97639308754ea15f6592b87cb884	V3V4		d__Bacteria;p__Firmicutes;c__Bacilli;o__Bacillales;f__Bacillaceae;g__Bacillus	4	0.00
52f955461f86ad883c55d8a3e8202279	V3V4		d__Bacteria;p__Proteobacteria;c__Gammaproteobacteria;o__Pseudomonadales;f__Moraxellaceae;g__Acinetobacter	5	0.01

c2b613466af9051a1e76bf383e85e2cf	V3V4		d__Bacteria;p__Firmicutes;c__Bacilli;o__Staphylococcales;f__Staphylococcaceae;g__Staphylococcus	3	0.00
c646867128acf18b40a0553724e2d3d9	V3V4		d__Bacteria;p__Firmicutes;c__Bacilli;o__Lactobacillales;f__Lactobacillaceae;g__Lactobacillus	1	0.00
c2e4ed60db611124a48fcecbc819041b	V3V4		d__Bacteria;p__Proteobacteria;c__Gammaproteobacteria;o__Pseudomonadales;f__Pseudomonadaceae;g__Pseudomonas	16	0.02
edd03326781345dce17c282af79662d9	V3V4		d__Bacteria;p__Firmicutes;c__Bacilli;o__Lactobacillales;f__Lactobacillaceae;g__Lactobacillus	1	0.00
03359371173b50f5325d305f0cf4ac20	V3V4		d__Bacteria;p__Actinobacteriota;c__Actinobacteria;o__Propionibacteriales;f__Propionibacteriaceae;g__Cutibacterium	4	0.00
11e8bc474b260bc0d712df6ea46b5199	V3V4			4	0.00
090b5dca9050c83d67c1b7482f9ec905	V3V4		d__Bacteria;p__Proteobacteria;c__Gammaproteobacteria;o__Pseudomonadales;f__Pseudomonadaceae;g__Pseudomonas	4	0.00
9713c18671402dea22a8cf2aaf4d75b7	V3V4		d__Bacteria;p__Actinobacteriota;c__Actinobacteria;o__Actinomycetales;f__Actinomycetaceae;g__Actinomyces	9	0.01
e63f1461c4c56b18a07fbf6cd5b09142	V3V4	d__Bacteria;p__Proteobacteria;c__Gammaproteobacteria;o__Enterobacterales;f__Enterobacteriaceae;g__Escherichia-Shigella;s__Malaclemys_terrapin	d__Bacteria;p__Proteobacteria;c__Gammaproteobacteria;o__Enterobacterales;f__Enterobacteriaceae;g__Escherichia-Shigella	1	0.00
44fe027804a633841108da2a936cf813	V3V4			1	0.00
f16b81eedac66ac1b68662de35eb5f5a	V3V4	d__Bacteria;p__Firmicutes;c__Bacilli;o__Lactobacillales;f__Streptococcaceae;g__Streptococcus;s__Streptococcus_troglodytae	d__Bacteria;p__Firmicutes;c__Bacilli;o__Lactobacillales;f__Streptococcaceae;g__Streptococcus	2	0.00
7132ebbc3bfce41e701cfe1f86ed45dd	V3V4	d__Bacteria;p__Firmicutes;c__Bacilli;o__Lactobacillales;f__Lactobacillaceae;g__Lactobacillus;s__uncultured_bacterium	d__Bacteria;p__Firmicutes;c__Bacilli;o__Lactobacillales;f__Lactobacillaceae;g__Lactobacillus	1	0.00

dc2ae076f87511208642bf8508d3e904	V3V4			1	0.00
d9a9cba2990f6a6c154303d3ec709205	V3V4		d__Bacteria;p__Proteobacteria;c__Gammaproteobacteria;o__Enterobacterales;f__Enterobacteriaceae;g__Escherichia-Shigella	1	0.00
f2ed2c40a65cdcd33edffde8adbeab62	V3V4		d__Bacteria;p__Actinobacteriota;c__Actinobacteria;o__Bifidobacteriales;f__Bifidobacteriaceae;g__Bifidobacterium	5	0.01
52f23ca04c38e4e75a6c8391c1a5cc57	V3V4		d__Bacteria;p__Proteobacteria;c__Alphaproteobacteria;o__Rhodobacterales;f__Rhodobacteraceae;g__Rhodobacter	2	0.00
88a0532c9cd77a5109be4a3c8136d363	V3V4	d__Bacteria;p__Deinococcota;c__Deinococci;o__Deinococcales;f__Deinococcaceae;g__Deinococcus;s__Deinococcus_radiodurans	d__Bacteria;p__Deinococcota;c__Deinococci;o__Deinococcales;f__Deinococcaceae;g__Deinococcus	7	0.01
c07ec260b3193f7708e297285494e549	V3V4		d__Bacteria;p__Firmicutes;c__Clostridia;o__Clostridiales;f__Clostridiaceae;g__Clostridium_sensu_stricto_1	1	0.00
fd6d065f1839c95af0c64f26f383a16c	V3V4	d__Bacteria;p__Firmicutes;c__Bacilli;o__Lactobacillales;f__Lactobacillaceae;g__Lactobacillus;s__mouse_gut	d__Bacteria;p__Firmicutes;c__Bacilli;o__Lactobacillales;f__Lactobacillaceae;g__Lactobacillus	2	0.00
50fdd882f9a2f686b0bb371512ce5c56	V3V4			9	0.01
183d55896ac0ec415369542ee7bc7d09	V3V4	d__Bacteria;p__Firmicutes;c__Bacilli;o__Lactobacillales;f__Streptococcaceae;g__Streptococcus;s__Streptococcus_troglodytae	d__Bacteria;p__Firmicutes;c__Bacilli;o__Lactobacillales;f__Streptococcaceae;g__Streptococcus	3	0.00
b1c1e045e4aa3c070d1ca97bb34c377f	V3V4			1	0.00
f3f6af8f3182835c90c5712e22748ccb	V3V4		d__Bacteria;p__Firmicutes;c__Bacilli;o__Staphylococcales;f__Staphylococcaceae;g__Staphylococcus	4	0.00
b0f8cd8129d46ed63ef0548638543eaf	V3V4		d__Bacteria;p__Proteobacteria;c__Gammaproteobacteria;o__Pseudomonadales;f__Moraxellaceae;g__Acinetobacter	1	0.00

84d98d85677ebac820163018f3b584b0	V3V4		d__Bacteria;p__Actinobacteriota;c__Actinobacteria;o__Propionibacteriales;f__Propionibacteriaceae;g__Cutibacterium	1	0.00
532b8c57ef373713b169f64953ceb7eb	V3V4		d__Bacteria;p__Proteobacteria;c__Gammaproteobacteria;o__Pseudomonadales;f__Pseudomonadaceae;g__Pseudomonas	1	0.00
4a8150ec3ef5381fe77e67c074d9d96f	V3V4	d__Bacteria;p__Proteobacteria;c__Gammaproteobacteria;o__Pseudomonadales;f__Pseudomonadaceae;g__Pseudomonas;s__uncultured_Marinobacter	d__Bacteria;p__Proteobacteria;c__Gammaproteobacteria;o__Pseudomonadales;f__Pseudomonadaceae;g__Pseudomonas	25	0.03
9246824a9517e9d0ff9477deab0a7ac7	V3V4		d__Bacteria;p__Firmicutes;c__Bacilli;o__Bacillales;f__Bacillaceae;g__Bacillus	2	0.00
314a64ec81e037428ef7102a4eb0b8da	V3V4		d__Bacteria;p__Firmicutes;c__Bacilli;o__Lactobacillales;f__Enterococcaceae;g__Enterococcus	2	0.00
a1cc90a21c23d2137d56bb6dc2921c4f	V3V4		d__Bacteria;p__Firmicutes;c__Bacilli;o__Lactobacillales;f__Lactobacillaceae;g__Lactobacillus	3	0.00
ec9e6cc25798a85f930369b2d00d82fd	V3V4		d__Bacteria;p__Firmicutes;c__Bacilli;o__Lactobacillales;f__Lactobacillaceae;g__Lactobacillus	3	0.00
1168280d13dbc651e68f5caf1d7bce82	V3V4		d__Bacteria;p__Firmicutes;c__Bacilli;o__Lactobacillales;f__Lactobacillaceae;g__Lactobacillus	1	0.00
71ae4c3f71b09401305f029a0f7d4643	V3V4			2	0.00
a9434f8c6924d8946a7a9f473c178038	V3V4		d__Bacteria;p__Firmicutes;c__Bacilli;o__Lactobacillales;f__Lactobacillaceae;g__Lactobacillus	2	0.00
293a8d61d2ae493e6eaf6996542ef8a6	V3V4		d__Bacteria;p__Firmicutes;c__Bacilli;o__Lactobacillales;f__Lactobacillaceae;g__Lactobacillus	2	0.00
988117d050ad203b779ce3d394bf89a2	V3V4			3	0.00
f05922e3d687273e586590a7e83a10a3	V3V4			2	0.00

967efff4d421800813303a3b0a03a194	V3V4	d__Bacteria;p__Firmicutes;c__Bacilli;o__Erysipelotrichales;f__Erysipelotrichaceae;g__Holdemanella	3	0.00
f12f13f1a547f87ab5808f9626d2f216	V3V4	d__Bacteria;p__Firmicutes;c__Clostridia;o__Lachnospirales;f__Lachnospiraceae;g__Blautia	12	0.01
3040e0c8ab58060a4e34cc39ef03da52	V3V4	d__Bacteria;p__Actinobacteriota;c__Actinobacteria;o__Actinomycetales;f__Actinomycetaceae;g__Actinomyces	1	0.00
8454e15db297968395307aef9bf3fde4	V3V4	d__Bacteria;p__Firmicutes;c__Bacilli;o__Staphylococcales;f__Staphylococcaceae;g__Staphylococcus	1	0.00
17ca9aee028bd10cc2bd929cbd0c54fb	V3V4	d__Bacteria;p__Firmicutes;c__Bacilli;o__Lactobacillales;f__Lactobacillaceae;g__Lactobacillus	1	0.00
233af22eb710eb9ddd3e0c2178da7f39	V3V4	d__Bacteria;p__Proteobacteria;c__Gammaproteobacteria;o__Enterobacterales;f__Enterobacteriaceae;g__Escherichia-Shigella	3	0.00
9c1d5c5045803e15a96a235f8de9dc24	V3V4	d__Bacteria;p__Actinobacteriota;c__Actinobacteria;o__Propionibacteriales;f__Propionibacteriaceae;g__Cutibacterium	3	0.00
55ecec4f9cd6b3f10605efc76a6823dc	V3V4	d__Bacteria;p__Firmicutes;c__Bacilli;o__Lactobacillales;f__Enterococcaceae;g__Enterococcus	2	0.00
488a8665cc64ed5fba5a8685d8df507a	V3V4		4	0.00
6023071f6f9e948bed1c969f0601d684	V3V4	d__Bacteria;p__Firmicutes;c__Bacilli;o__Lactobacillales;f__Enterococcaceae;g__Enterococcus	4	0.00
4be7df120f0d1c5c7c0e6079210d6042	V3V4	d__Bacteria;p__Proteobacteria;c__Alphaproteobacteria;o__Rhodobacterales;f__Rhodobacteraceae;g__Rhodobacter	1	0.00
3a493d430b6c9ebfc351bdf059799e78	V3V4	d__Bacteria;p__Firmicutes;c__Bacilli;o__Lactobacillales;f__Lactobacillaceae;g__Lactobacillus	2	0.00
ce388ec374f5d252945e6b8743ee90c0	V3V4	d__Bacteria;p__Deinococcota;c__Deinococci;o__Deinococcales;f__Deinococcaceae;g__Deinococcus	5	0.01

fa91d0754e33b45a3cda25ae630c7eb1	V3V4		d__Bacteria;p__Actinobacteriota;c__Actinobacteria;o__Propionibacteriales;f__Propionibacteriaceae;g__Cutibacterium	1	0.00
5ef26d58b743e2c2b49b51badcbf8900	V3V4			4	0.00
9e10aab3874583d56021f515a5c4cbf4	V3V4		d__Bacteria;p__Firmicutes;c__Clostridia;o__Clostridiales;f__Clostridiaceae;g__Clostridium_sensu_stricto_1	1	0.00
0253bd5f7388df35c5f43b2d85b267fa	V3V4			1	0.00
3942209bbeeb672021ff36f82794ae6e	V3V4	d__Bacteria;p__Campilobacterota;c__Campylobacteria;o__Campylobacterales;f__Helicobacteraceae;g__Helicobacter;s__uncultured_Helicobacter	d__Bacteria;p__Campilobacterota;c__Campylobacteria;o__Campylobacterales;f__Helicobacteraceae;g__Helicobacter	4	0.00
e0c987cbfa9d14ae13e9cf00023dd23b	V3V4			6	0.01
cb81aae5ff4960b2c64970d7c8ef0cb1	V3V4			2	0.00
1f9d41814f8c4832104632e6be3add7d	V3V4		d__Bacteria;p__Proteobacteria;c__Gammaproteobacteria;o__Enterobacterales;f__Enterobacteriaceae;g__Escherichia-Shigella	12	0.01
de6c5767675daea2809d212ff3e6db03	V3V4			2	0.00
acab893a2330effacc9f74ae5a59944f	V3V4		d__Bacteria;p__Proteobacteria;c__Gammaproteobacteria;o__Burkholderiales;f__Neisseriaceae;g__Neisseria	3	0.00
b4393dd2898000d8bf94ad374a84e508	V3V4		d__Bacteria;p__Firmicutes;c__Bacilli;o__Lactobacillales;f__Lactobacillaceae;g__Lactobacillus	1	0.00
3534d05428b2f9d14741eb884c9a3144	V3V4		d__Bacteria;p__Actinobacteriota;c__Actinobacteria;o__Propionibacteriales;f__Propionibacteriaceae;g__Cutibacterium	1	0.00
6b1e9704d9c7b3dcfcfdba6c995e64d8	V3V4	d__Bacteria;p__Proteobacteria;c__Gammaproteobacteria;o__Enterobacterales;f__Enterobacteriaceae;g__Escherichia-Shigella;s__Streptomyces_sp.	d__Bacteria;p__Proteobacteria;c__Gammaproteobacteria;o__Enterobacterales;f__Enterobacteriaceae;g__Escherichia-Shigella	4	0.00

36b49d1cdea36609242c463e217b7880	V3V4		d__Bacteria;p__Actinobacteriota;c__Actinobacteria;o__Propionibacteriales;f__Propionibacteriaceae;g__Cutibacterium	7	0.01
03b94133f169109cf1d00b403c5f0989	V3V4		d__Bacteria;p__Actinobacteriota;c__Actinobacteria;o__Bifidobacteriales;f__Bifidobacteriaceae;g__Bifidobacterium	7	0.01
9674fc3295b7329cfdf903d5da7838ee	V3V4		d__Bacteria;p__Proteobacteria;c__Gammaproteobacteria;o__Pseudomonadales;f__Pseudomonadaceae;g__Pseudomonas	1	0.00
1face95889737aec126a40991f8032ef	V3V4		d__Bacteria;p__Actinobacteriota;c__Actinobacteria;o__Bifidobacteriales;f__Bifidobacteriaceae;g__Bifidobacterium	1	0.00
06ec07e2478fcd1a6a1420ba11e83e3c	V3V4		d__Bacteria;p__Proteobacteria;c__Gammaproteobacteria;o__Enterobacterales;f__Enterobacteriaceae;g__Escherichia-Shigella	4	0.00
63c4d6d4257da3014c495f25430053a5	V3V4		d__Bacteria;p__Proteobacteria;c__Gammaproteobacteria;o__Enterobacterales;f__Enterobacteriaceae;g__Escherichia-Shigella	2	0.00
8fafdc7828b72571545121e1f3d36a36	V3V4		d__Bacteria;p__Proteobacteria;c__Gammaproteobacteria;o__Pseudomonadales;f__Pseudomonadaceae;g__Pseudomonas	10	0.01
9fbc75ff0f6bf962fdbf13b855503241	V3V4		d__Bacteria;p__Actinobacteriota;c__Actinobacteria;o__Bifidobacteriales;f__Bifidobacteriaceae;g__Bifidobacterium	3	0.00
007a2dac44d3879720067e0521158cf0	V3V4		d__Bacteria;p__Firmicutes;c__Bacilli;o__Lactobacillales;f__Enterococcaceae;g__Enterococcus	3	0.00
27ed56bde0d4235bf1f6a57e9da3e72c	V3V4		d__Bacteria;p__Deinococcota;c__Deinococci;o__Deinococcales;f__Deinococcaceae;g__Deinococcus	3	0.00
70e63d68330c3281404f22001f081c99	V3V4		d__Bacteria;p__Proteobacteria;c__Gammaproteobacteria;o__Pseudomonadales;f__Pseudomonadaceae;g__Pseudomonas	6	0.01

b1b42f4f1c0d778afcd2ceebbbaf5dbc					V3V4				d__Bacteria;p__Bacteroidota;c__Bacteroidia;o__Bacteroidales;f__Bacteroidaceae;g__Bacteroides	3	0.00
33aea641c7dd0a325acf8de4e821c12d					V3V4					2	0.00
b7756945b974867c86021564547c2a6d					V3V4					3	0.00
48705a13153b62ac0af4ab27b73192e3					V3V4			d__Bacteria;p__Firmicutes;c__Bacilli;o__Lactobacillales;f__Streptococcaceae;g__Streptococcus;s__uncultured_prokaryote	d__Bacteria;p__Firmicutes;c__Bacilli;o__Lactobacillales;f__Streptococcaceae;g__Streptococcus	6	0.01
1b5ca727a776fec4cc49a2e3388a7bd5					V3V4					2	0.00
a47fbbed7375d0d17895e59409f03a53					V3V4				d__Bacteria;p__Firmicutes;c__Bacilli;o__Lactobacillales;f__Enterococcaceae;g__Enterococcus	1	0.00
da79262b440ec0361d03c434e6dd7956					V3V4				d__Bacteria;p__Firmicutes;c__Bacilli;o__Lactobacillales;f__Streptococcaceae;g__Streptococcus	1	0.00
362e0b0c0223e3f1e0f39abbd4f87eeb					V3V4				d__Bacteria;p__Proteobacteria;c__Alphaproteobacteria;o__Rhodobacterales;f__Rhodobacteraceae;g__Rhodobacter	1	0.00
d285d1af9dd4fe2762db30c758452a4c					V3V4					2	0.00
e202f18d818883a9a0e8a8019c46d960	1eb3021faaec49c2_1	97.196	455325	455751	V3V4	Escherichia_coli_ATCC_700926	True		d__Bacteria;p__Proteobacteria;c__Gammaproteobacteria;o__Enterobacterales;f__Enterobacteriaceae;g__Escherichia-Shigella	1	0.00
5497318e515a8c328a68f95975d9c7d4	f5bcb58692924cb7_1	100	1597643	1597217	V3V4	Staphylococcus_epidermidis_ATCC_12228	True		d__Bacteria;p__Firmicutes;c__Bacilli;o__Staphylococcales;f__Staphylococcaceae;g__Staphylococcus	5.15E+03	5.17
2daa31dbdedabfa287858d52c4ade4d7	f5bcb58692924cb7_1	99.063	1597643	1597217	V3V4	Staphylococcus_epidermidis_ATCC_12228	True		d__Bacteria;p__Firmicutes;c__Bacilli;o__Staphylococcales;f__Staphylococcaceae;g__Staphylococcus	10	0.01

d33a6211611cada51a0eda6d00b3f45f	c45a1c57adf4460e_2	100	23603	23859	V3V4	Bifidobacterium_adolescentis_ATCC_15703	True		d__Bacteria;p__Actinobacteriota;c__Actinobacteria;o__Bifidobacteriales;f__Bifidobacteriaceae;g__Bifidobacterium	31	0.03
9a004eaacc9a867ffef598974a724e24	f5bcb58692924cb7_1	98.364	1597643	1597217	V3V4	Staphylococcus_epidermidis_ATCC_12228	True		d__Bacteria;p__Firmicutes;c__Bacilli;o__Staphylococcales;f__Staphylococcaceae;g__Staphylococcus	2	0.00
5c09851da6fa2dce3f470ae14beeb4d1	58ae911973b74ffc_1	99.766	16666	17092	V3V4	Streptococcus_agalactiae_ATCC_BAA_611	True	d__Bacteria;p__Firmicutes;c__Bacilli;o__Lactobacillales;f__Streptococcaceae;g__Streptococcus;s__Streptococcus_agalactiae	d__Bacteria;p__Firmicutes;c__Bacilli;o__Lactobacillales;f__Streptococcaceae;g__Streptococcus	210	0.21
7aa282961d474a9fe44dea17c37b2cf5	58ae911973b74ffc_1	99.766	16666	17092	V3V4	Streptococcus_agalactiae_ATCC_BAA_611	True	d__Bacteria;p__Firmicutes;c__Bacilli;o__Lactobacillales;f__Streptococcaceae;g__Streptococcus;s__Streptococcus_agalactiae	d__Bacteria;p__Firmicutes;c__Bacilli;o__Lactobacillales;f__Streptococcaceae;g__Streptococcus	1	0.00
ae724118f1bdef6e863be0561de2aaff	1eb3021faaec49c2_1	99.766	455325	455751	V3V4	Escherichia_coli_ATCC_700926	True		d__Bacteria;p__Proteobacteria;c__Gammaproteobacteria;o__Enterobacterales;f__Enterobacteriaceae;g__Escherichia-Shigella	33	0.03
9a6453463075448b4ef5e1b93fe2e362	5964adb8d0df4fde_1	99.766	477829	478255	V3V4	Lactobacillus_gasseri_ATCC_33323	True	d__Bacteria;p__Firmicutes;c__Bacilli;o__Lactobacillales;f__Lactobacillaceae;g__Lactobacillus;s__Lactobacillus_gasseri	d__Bacteria;p__Firmicutes;c__Bacilli;o__Lactobacillales;f__Lactobacillaceae;g__Lactobacillus	2070	2.08
88de790328dd10b3ebad7cb963d17770	adb6d513b7374f84_1	98.444	17219	17474	V3V4	Streptococcus_mutans_ATCC_700610	True		d__Bacteria;p__Firmicutes;c__Bacilli;o__Lactobacillales;f__Streptococcaceae;g__Streptococcus	2	0.00
cb9c3959a2d40cbc54a6fb9714c881ca	5964adb8d0df4fde_1	99.766	477829	478255	V3V4	Lactobacillus_gasseri_ATCC_33323	True	d__Bacteria;p__Firmicutes;c__Bacilli;o__Lactobacillales;f__Lactobacillaceae;g__Lactobacillus;s__Lactobacillus_gasseri	d__Bacteria;p__Firmicutes;c__Bacilli;o__Lactobacillales;f__Lactobacillaceae;g__Lactobacillus	5	0.01
51732e34618ee0de8cb1b1d79c585fc3	5964adb8d0df4fde_1	98.829	477829	478255	V3V4	Lactobacillus_gasseri_ATCC_33323	True		d__Bacteria;p__Firmicutes;c__Bacilli;o__Lactobacillales;f__Lactobacillaceae;g__Lactobacillus	2.00E+00	0.00
24af7433c4486d1fd3fd32423ad699b5	045df7c7cb0f42b5_1	99.203	1222984	1222734	V3V4	Neisseria_meningitidis_ATCC_BAA_335	True		d__Bacteria;p__Proteobacteria;c__Gammaproteobacteria;o__Burkholderiales;f__Neisseriaceae;g__Neisseria	2	0.00

b6635d67cb594473ddba9f8cfba5d13d	d889f9dd971b473d_1	100	347873	347452	V3V4	Phocaeicola_vulgatus_ATCC_8482	True	d__Bacteria;p__Bacteroidota;c__Bacteroidia;o__Bacteroidales;f__Bacteroidaceae;g__Bacteroides;s__Bacteroides_vulgatus	d__Bacteria;p__Bacteroidota;c__Bacteroidia;o__Bacteroidales;f__Bacteroidaceae;g__Bacteroides	4744	4.76
e4c0868fdefcdf2037ab5f7a071e4cc7	2b7fa0e1de5f42de_1	100	182859	183272	V3V4	Deinococcus_radiodurans_ATCC_BAA_816	True	d__Bacteria;p__Deinococcota;c__Deinococci;o__Deinococcales;f__Deinococcaceae;g__Deinococcus;s__Deinococcus_radiodurans	d__Bacteria;p__Deinococcota;c__Deinococci;o__Deinococcales;f__Deinococcaceae;g__Deinococcus	2036	2.04
318669d5d926e9b81ca6911da00a14ea	3042186b85304e8d_1	100	9691	10092	V3V4	Clostridium_beijerinckii_ATCC_35702	True		d__Bacteria;p__Firmicutes;c__Clostridia;o__Clostridiales;f__Clostridiaceae;g__Clostridium_sensu_stricto_1	12195	12.25
64a7212b599e1f720f712f4a72f567a2	adb6d513b7374f84_1	97.613	17048	17465	V3V4	Streptococcus_mutans_ATCC_700610	True	d__Bacteria;p__Firmicutes;c__Bacilli;o__Lactobacillales;f__Streptococcaceae;g__Streptococcus;s__Streptococcus_mutans	d__Bacteria;p__Firmicutes;c__Bacilli;o__Lactobacillales;f__Streptococcaceae;g__Streptococcus	1	0.00
b4d126d7b5117251ec9a72501c1093c8	5964adb8d0df4fde_1	97.658	477829	478255	V3V4	Lactobacillus_gasseri_ATCC_33323	True	d__Bacteria;p__Firmicutes;c__Bacilli;o__Lactobacillales;f__Lactobacillaceae;g__Lactobacillus;s__uncultured_bacterium	d__Bacteria;p__Firmicutes;c__Bacilli;o__Lactobacillales;f__Lactobacillaceae;g__Lactobacillus	2	0.00
63fcaf02bc7b34ab33308d40414e7307	d889f9dd971b473d_1	99.526	347873	347452	V3V4	Phocaeicola_vulgatus_ATCC_8482	True	d__Bacteria;p__Bacteroidota;c__Bacteroidia;o__Bacteroidales;f__Bacteroidaceae;g__Bacteroides;s__Bacteroides_vulgatus	d__Bacteria;p__Bacteroidota;c__Bacteroidia;o__Bacteroidales;f__Bacteroidaceae;g__Bacteroides	822	0.83
908e9b387f6b9ce7d3f794e658fba37e	f5bcb58692924cb7_1	99.766	1597643	1597217	V3V4	Staphylococcus_epidermidis_ATCC_12228	True		d__Bacteria;p__Firmicutes;c__Bacilli;o__Staphylococcales;f__Staphylococcaceae;g__Staphylococcus	897	0.90
056129de3ff9eab079fac9ec56181a4a	2504aeb524e74d25_1	100	229673	230094	V3V4	Porphyromonas_gingivalis_ATCC_33277	True	d__Bacteria;p__Bacteroidota;c__Bacteroidia;o__Bacteroidales;f__Porphyromonadaceae;g__Porphyromonas;s__Porphyromonas_gingivalis	d__Bacteria;p__Bacteroidota;c__Bacteroidia;o__Bacteroidales;f__Porphyromonadaceae;g__Porphyromonas	6361	6.39
afa4f6bdca4c647eb4492953ac855721	c45a1c57adf4460e_2	99.757	23449	23859	V3V4	Bifidobacterium_adolescentis_ATCC_15703	True		d__Bacteria;p__Actinobacteriota;c__Actinobacteria;o__Bifidobacteriales;f__Bifidobacteriaceae;g__Bifidobacterium	147	0.15
3f3a0eaeea9c0690b6ede1b17b4fd8ce	c45a1c57adf4460e_2	100	23449	23859	V3V4	Bifidobacterium_adolescentis_	True	d__Bacteria;p__Actinobacteriota;c__Actinobacteria;o__Bifidobacteriales;f__Bifidobacteriaceae;g__Bifidobacter	d__Bacteria;p__Actinobacteriota;c__Actinobacteria;o__Bifidobacteriales;f__Bifidobacteriaceae;g__Bifidobacterium	3815	3.83

						ATCC_15703		ium;s__Bifidobacterium_adolescentis			
521820dea22098c03e64451bdeca66be	c45a1c57adf4460e_2	98.718	23626	23859	V3V4	Bifidobacterium_adolescentis_ATCC_15703	True		d__Bacteria;p__Actinobacteriota;c__Actinobacteria;o__Bifidobacteriales;f__Bifidobacteriaceae;g__Bifidobacterium	12	0.01
e31c7e269125382d9bd6ee8fcc1fde21	d889f9dd971b473d_1	99.527	347873	347452	V3V4	Phocaeicola_vulgatus_ATCC_8482	True	d__Bacteria;p__Bacteroidota;c__Bacteroidia;o__Bacteroidales;f__Bacteroidaceae;g__Bacteroides;s__Bacteroides_vulgatus	d__Bacteria;p__Bacteroidota;c__Bacteroidia;o__Bacteroidales;f__Bacteroidaceae;g__Bacteroides	965	0.97
332b70897316f7f62b81dfc53f41ca52	266a835027f7464b_2	100	38225	37824	V3V4	Rhodobacter_sphaeroides_ATCC_17029	True	d__Bacteria;p__Proteobacteria;c__Alphaproteobacteria;o__Rhodobacterales;f__Rhodobacteraceae;g__Rhodobacter;s__Rhodobacter_sphaeroides	d__Bacteria;p__Proteobacteria;c__Alphaproteobacteria;o__Rhodobacterales;f__Rhodobacteraceae;g__Rhodobacter	762	0.77
ffc36e27c82042664a16bcd4d380b286	1eb3021faaec49c2_1	100	455325	455751	V3V4	Escherichia_coli_ATCC_700926	True		d__Bacteria;p__Proteobacteria;c__Gammaproteobacteria;o__Enterobacterales;f__Enterobacteriaceae;g__Escherichia-Shigella	6772	6.80
f71e5aacd4c976a1833958c2870b1d8b	045df7c7cb0f42b5_1	100	1223181	1222755	V3V4	Neisseria_meningitidis_ATCC_BAA_335	True	d__Bacteria;p__Proteobacteria;c__Gammaproteobacteria;o__Burkholderiales;f__Neisseriaceae;g__Neisseria;s__Neisseria_meningitidis	d__Bacteria;p__Proteobacteria;c__Gammaproteobacteria;o__Burkholderiales;f__Neisseriaceae;g__Neisseria	3480	3.50
88a3a8e95e3605bd051054f937cde102	3042186b85304e8d_1	99.751	9691	10092	V3V4	Clostridium_beijerinckii_ATCC_35702	True		d__Bacteria;p__Firmicutes;c__Clostridia;o__Clostridiales;f__Clostridiaceae;g__Clostridium_sensu_stricto_1	904	0.91
e8de9432f2ada1078a2fda56ba92675a	4762fdd895094939_1	100	96706	97108	V3V4	Helicobacter_pylori_ATCC_700392	True	d__Bacteria;p__Campilobacterota;c__Campylobacteria;o__Campylobacterales;f__Helicobacteraceae;g__Helicobacter;s__Helicobacter_pylori	d__Bacteria;p__Campilobacterota;c__Campylobacteria;o__Campylobacterales;f__Helicobacteraceae;g__Helicobacter	3097	3.11
052ba7abaeaa968c4f79e3f97d1f0a2f	285a115d88c043f1_1	100	678693	679119	V3V4	Pseudomonas_aeruginosa_ATCC_9027	True		d__Bacteria;p__Proteobacteria;c__Gammaproteobacteria;o__Pseudomonadales;f__Pseudomonadaceae;g__Pseudomonas	2581	2.59
c42488aff4cc842bf285a401dba39cc4	89e35fec14744942_1	100	528661	529080	V3V4	Schaalia_odontolytica_ATCC_17982	True	d__Bacteria;p__Actinobacteriota;c__Actinobacteria;o__Actinomycetales;f__Actinomycetaceae;g__Actinomyces;s__Schaalia_odontolytica	d__Bacteria;p__Actinobacteriota;c__Actinobacteria;o__Actinomycetales;f__Actinomycetaceae;g__Actinomyces	2260	2.27
b05f5459e068b529b2c41e659efdef34	9dc637a985b042c4_1	100	605572	605971	V3V4	Cutibacterium_acnes_ATCC_11828	True		d__Bacteria;p__Actinobacteriota;c__Actinobacteria;o__Propionibacteriales;f__Propionibacteriaceae;g__Cutibacterium	411	0.41

892a20bbdc3ce599dc0c5d9f0866c352	27b35dd1651e48dd_1	100	213739	214165	V3V4	Enterococcus_faecalis_ATCC_47077	True		d__Bacteria;p__Firmicutes;c__Bacilli;o__Lactobacillales;f__Enterococcaceae;g__Enterococcus	2860	2.87
5c035b54ac1cea11c1da765208a66520	5964adb8d0df4fde_1	99.766	477829	478255	V3V4	Lactobacillus_gasseri_ATCC_33323	True	d__Bacteria;p__Firmicutes;c__Bacilli;o__Lactobacillales;f__Lactobacillaceae;g__Lactobacillus;s__Lactobacillus_gasseri	d__Bacteria;p__Firmicutes;c__Bacilli;o__Lactobacillales;f__Lactobacillaceae;g__Lactobacillus	1747	1.75
c3a3a503752209bc052b3995236b079f	58ae911973b74ffc_1	100	16666	17092	V3V4	Streptococcus_agalactiae_ATCC_BAA_611	True	d__Bacteria;p__Firmicutes;c__Bacilli;o__Lactobacillales;f__Streptococcaceae;g__Streptococcus;s__Streptococcus_agalactiae	d__Bacteria;p__Firmicutes;c__Bacilli;o__Lactobacillales;f__Streptococcaceae;g__Streptococcus	6597	6.63
04be702b895fe5ed0568344daf564276	38ad2dab3e5e43b7_1	100	628061	628487	V3V4	Staphylococcus_aureus_subsp_aureus_ATCC_BAA_1556	True		d__Bacteria;p__Firmicutes;c__Bacilli;o__Staphylococcales;f__Staphylococcaceae;g__Staphylococcus	4304	4.32
ef5af48ec2b6c023c5de28c59cb08a40	adb6d513b7374f84_1	100	17048	17474	V3V4	Streptococcus_mutans_ATCC_700610	True	d__Bacteria;p__Firmicutes;c__Bacilli;o__Lactobacillales;f__Streptococcaceae;g__Streptococcus;s__Streptococcus_mutans	d__Bacteria;p__Firmicutes;c__Bacilli;o__Lactobacillales;f__Streptococcaceae;g__Streptococcus	6030	6.06
99f8f7fb3dc2bb4e0990397d1c9e5dc4	38ad2dab3e5e43b7_1	99.766	628061	628487	V3V4	Staphylococcus_aureus_subsp_aureus_ATCC_BAA_1556	True		d__Bacteria;p__Firmicutes;c__Bacilli;o__Staphylococcales;f__Staphylococcaceae;g__Staphylococcus	804	0.81
194692a65aff7f21d0d0029503feeed5	adb6d513b7374f84_1	99.766	17048	17474	V3V4	Streptococcus_mutans_ATCC_700610	True	d__Bacteria;p__Firmicutes;c__Bacilli;o__Lactobacillales;f__Streptococcaceae;g__Streptococcus;s__Streptococcus_mutans	d__Bacteria;p__Firmicutes;c__Bacilli;o__Lactobacillales;f__Streptococcaceae;g__Streptococcus	227	0.23
4e7848491cf85b1d56d3c920629ba5de	285a115d88c043f1_1	97.418	678695	679119	V3V4	Pseudomonas_aeruginosa_ATCC_9027	True		d__Bacteria;p__Proteobacteria;c__Gammaproteobacteria;o__Pseudomonadales;f__Pseudomonadaceae;g__Pseudomonas	2.30E+01	0.02
b8f980ec6ad0c86efb83bd1f64c3a252	d889f9dd971b473d_1	99.763	347873	347452	V3V4	Phocaeicola_vulgatus_ATCC_8482	True	d__Bacteria;p__Bacteroidota;c__Bacteroidia;o__Bacteroidales;f__Bacteroidaceae;g__Bacteroides;s__Bacteroides_vulgatus	d__Bacteria;p__Bacteroidota;c__Bacteroidia;o__Bacteroidales;f__Bacteroidaceae;g__Bacteroides	1030	1.03
3f298e5dbea50d14bc52110ff80df5c0	5964adb8d0df4fde_1	97.19	477829	478255	V3V4	Lactobacillus_gasseri	True		d__Bacteria;p__Firmicutes;c__Bacilli;o__Lactobacillales;f__Lactobacillaceae;g__Lactobacillus	1	0.00

						_ATCC_33323					
c49cc7c2c45bd7a87913453e515ea14f	071a82bb90b94c6f_1	100	495134	495561	V3V4	Acinetobacter_baumannii_ATCC_17978	True	d__Bacteria;p__Proteobacteria;c__Gammaproteobacteria;o__Pseudomonadales;f__Moraxellaceae;g__Acinetobacter;s__Acinetobacter_baumannii	d__Bacteria;p__Proteobacteria;c__Gammaproteobacteria;o__Pseudomonadales;f__Moraxellaceae;g__Acinetobacter	6724	6.75
170cb61a735e0bfe7a859f4613210635	27b35dd1651e48dd_1	98.084	213906	214165	V3V4	Enterococcus_faecalis_ATCC_47077	True		d__Bacteria;p__Firmicutes;c__Bacilli;o__Lactobacillales;f__Enterococcaceae;g__Enterococcus	1	0.00
8cb24777cb48dde0aac60dfeca125d10	5371a9904eb64361_1	100	9266	9693	V3V4	Bacillus_pacificus_ATCC_10987	True		d__Bacteria;p__Firmicutes;c__Bacilli;o__Bacillales;f__Bacillaceae;g__Bacillus	7565	7.60
f85cf38f782a314e3ab5965a58f2e46e	285a115d88c043f1_1	99.766	678693	679119	V3V4	Pseudomonas_aeruginosa_ATCC_9027	True		d__Bacteria;p__Proteobacteria;c__Gammaproteobacteria;o__Pseudomonadales;f__Pseudomonadaceae;g__Pseudomonas	1	0.00
c4252de734fefd8ff07ede127685ee56	f5bcb58692924cb7_1	98.834	1597643	1597217	V3V4	Staphylococcus_epidermidis_ATCC_12228	True		d__Bacteria;p__Firmicutes;c__Bacilli;o__Staphylococcales;f__Staphylococcaceae;g__Staphylococcus	1	0.00
9b23053de8f4269fe6b5ce286dfbef3c	5964adb8d0df4fde_1	100	477829	478255	V3V4	Lactobacillus_gasseri_ATCC_33323	True	d__Bacteria;p__Firmicutes;c__Bacilli;o__Lactobacillales;f__Lactobacillaceae;g__Lactobacillus;s__Lactobacillus_gasseri	d__Bacteria;p__Firmicutes;c__Bacilli;o__Lactobacillales;f__Lactobacillaceae;g__Lactobacillus	1153	1.16
c1f46ee956e06530a83aa00914e4c7cb	adb6d513b7374f84_1	99.766	17048	17474	V3V4	Streptococcus_mutans_ATCC_700610	True	d__Bacteria;p__Firmicutes;c__Bacilli;o__Lactobacillales;f__Streptococcaceae;g__Streptococcus;s__Streptococcus_mutans	d__Bacteria;p__Firmicutes;c__Bacilli;o__Lactobacillales;f__Streptococcaceae;g__Streptococcus	1	0.00
3f460e58e5d5db0dbaf1a71ceef1525d	5964adb8d0df4fde_1	97.424	477829	478255	V3V4	Lactobacillus_gasseri_ATCC_33323	True	d__Bacteria;p__Firmicutes;c__Bacilli;o__Lactobacillales;f__Lactobacillaceae;g__Lactobacillus;s__uncultured_bacterium	d__Bacteria;p__Firmicutes;c__Bacilli;o__Lactobacillales;f__Lactobacillaceae;g__Lactobacillus	5	0.01
f8cad6522103a22e0788a989a039a794	266a835027f7464b_2	97.79	37792	37612	V5V6	Rhodobacter_sphaeroides_ATCC_17029	True	d__Bacteria;p__Proteobacteria;c__Alphaproteobacteria;o__Rhodobacterales;f__Rhodobacteraceae;g__Paracoccus;s__uncultured_Paracoccus	d__Bacteria;p__Proteobacteria;c__Alphaproteobacteria;o__Rhodobacterales;f__Rhodobacteraceae;g__Paracoccus	24	0.03
c640b138c428758d0ee70015ffffefbd	58ae911973b74ffc_1	97.382	17124	17314	V5V6	Streptococcus_agalactiae_ATCC_BAA_611	True	d__Bacteria;p__Firmicutes;c__Bacilli;o__Lactobacillales;f__Streptococcaceae;g__Streptococcus;s__Streptococcus_gordonii	d__Bacteria;p__Firmicutes;c__Bacilli;o__Lactobacillales;f__Streptococcaceae;g__Streptococcus	137	0.20

75e81af1ea94c080a5e24422bf0f8270	5371a9904eb64361_1	97.283	9725	9908	V5V6	Bacillus_pacificus_ATCC_10987	True		d__Bacteria;p__Firmicutes;c__Bacilli;o__Bacillales;f__Bacillaceae;g__Bacillus	199	0.28
41ad2a26bdea29ce20ce5c2f716b71e5	5964adb8d0df4fde_1	97.283	478287	478470	V5V6	Lactobacillus_gasseri_ATCC_33323	True	d__Bacteria;p__Firmicutes;c__Bacilli;o__Lactobacillales;f__Enterococcaceae;g__Enterococcus;s__Enterococcus_mundtii	d__Bacteria;p__Firmicutes;c__Bacilli;o__Lactobacillales;f__Enterococcaceae;g__Enterococcus	137	0.20
b9a27f523947e6b31eaff94f591a55de	5371a9904eb64361_1	100	9725	9924	V5V6	Bacillus_pacificus_ATCC_10987	True		d__Bacteria;p__Firmicutes;c__Bacilli;o__Bacillales;f__Bacillaceae;g__Bacillus	312	0.44
1d9d72ff20c4f8425b3aa55e1d742dc0	285a115d88c043f1_1	97.802	679151	679332	V5V6	Pseudomonas_aeruginosa_ATCC_9027	True	d__Bacteria;p__Proteobacteria;c__Gammaproteobacteria;o__Pseudomonadales;f__Pseudomonadaceae;g__Pseudomonas;s__Pseudomonas_aeruginosa	d__Bacteria;p__Proteobacteria;c__Gammaproteobacteria;o__Pseudomonadales;f__Pseudomonadaceae;g__Pseudomonas	72	0.10
4ba5a36a442f7295337341bae584d319	3042186b85304e8d_1	99.5	10124	10323	V5V6	Clostridium_beijerinckii_ATCC_35702	True	d__Bacteria;p__Firmicutes;c__Clostridia;o__Clostridiales;f__Clostridiaceae;g__Clostridium_sensu_stricto_1;s__uncultured_Clostridium	d__Bacteria;p__Firmicutes;c__Clostridia;o__Clostridiales;f__Clostridiaceae;g__Clostridium_sensu_stricto_1	1536	2.19
42ae093ca8e3d07f4f5318f40d0a20da	3042186b85304e8d_1	100	10124	10340	V5V6	Clostridium_beijerinckii_ATCC_35702	True	d__Bacteria;p__Firmicutes;c__Clostridia;o__Clostridiales;f__Clostridiaceae;g__Clostridium_sensu_stricto_1;s__uncultured_Clostridium	d__Bacteria;p__Firmicutes;c__Clostridia;o__Clostridiales;f__Clostridiaceae;g__Clostridium_sensu_stricto_1	99	0.14
b7f8ec8c56ea6c5a877b899bfa65f6cf	27b35dd1651e48dd_1	97.283	214197	214380	V5V6	Enterococcus_faecalis_ATCC_47077	True		d__Bacteria;p__Firmicutes;c__Bacilli;o__Lactobacillales;f__Enterococcaceae;g__Enterococcus	90	0.13
d00984415738187f2bd75ba771f699d4	3042186b85304e8d_1	99.5	10124	10323	V5V6	Clostridium_beijerinckii_ATCC_35702	True	d__Bacteria;p__Firmicutes;c__Clostridia;o__Clostridiales;f__Clostridiaceae;g__Clostridium_sensu_stricto_1;s__uncultured_Clostridium	d__Bacteria;p__Firmicutes;c__Clostridia;o__Clostridiales;f__Clostridiaceae;g__Clostridium_sensu_stricto_1	3582	5.11
e0604154e9581bcb07e1f13c57121882	f5bcb58692924cb7_1	99.502	1597185	1596986	V5V6	Staphylococcus_epidermidis_ATCC_12228	True	d__Bacteria;p__Firmicutes;c__Bacilli;o__Staphylococcales;f__Staphylococcaceae;g__Staphylococcus;s__Staphylococcus_epidermidis	d__Bacteria;p__Firmicutes;c__Bacilli;o__Staphylococcales;f__Staphylococcaceae;g__Staphylococcus	15	0.02
79dbaba672642ebd014b1e7b9b328112	071a82bb90b94c6f_1	100	19023	19242	V5V6	Acinetobacter_baumannii_ATCC_17978	True		d__Bacteria;p__Proteobacteria;c__Gammaproteobacteria;o__Pseudomonadales;f__Moraxellaceae;g__Acinetobacter	8033	11.45

6a2f7caa6b9c38ac8d74597de800bc87	2504aeb524e74d25_1	100	230126	230325	V5V6	Porphyromonas_gingivalis_ATCC_33277	True			8152	11.62
012b9a89e0c38429987cfa5126ca9e98	58ae911973b74ffc_1	100	17124	17323	V5V6	Streptococcus_agalactiae_ATCC_BAA_611	True	d__Bacteria;p__Firmicutes;c__Bacilli;o__Lactobacillales;f__Streptococcaceae;g__Streptococcus;s__Streptococcus_gordonii	d__Bacteria;p__Firmicutes;c__Bacilli;o__Lactobacillales;f__Streptococcaceae;g__Streptococcus	202	0.29
e0a2bef6846fbd1aa22828c8a8086c86	89e35fec14744942_1	100	529112	529324	V5V6	Schaalia_odontolytica_ATCC_17982	True		d__Bacteria;p__Actinobacteriota;c__Actinobacteria;o__Actinomycetales;f__Actinomycetaceae;g__Actinomyces	141	0.20
21d6f50624ff01e5bee69a84112530a0	f5bcb58692924cb7_1	100	1597185	1596986	V5V6	Staphylococcus_epidermidis_ATCC_12228	True	d__Bacteria;p__Firmicutes;c__Bacilli;o__Staphylococcales;f__Staphylococcaceae;g__Staphylococcus;s__Staphylococcus_sp.	d__Bacteria;p__Firmicutes;c__Bacilli;o__Staphylococcales;f__Staphylococcaceae;g__Staphylococcus	9126	13.01
3704c6a53ad64ba61b11b9bb6e7e06f7	3042186b85304e8d_1	100	10124	10323	V5V6	Clostridium_beijerinckii_ATCC_35702	True	d__Bacteria;p__Firmicutes;c__Clostridia;o__Clostridiales;f__Clostridiaceae;g__Clostridium_sensu_stricto_1;s__uncultured_Clostridium	d__Bacteria;p__Firmicutes;c__Clostridia;o__Clostridiales;f__Clostridiaceae;g__Clostridium_sensu_stricto_1	21062	30.03
56c7c15ecc0af90185a9c2e39af9a4a4	1eb3021faaec49c2_1	100	455783	455994	V5V6	Escherichia_coli_ATCC_700926	True	d__Bacteria;p__Proteobacteria;c__Gammaproteobacteria;o__Enterobacterales;f__Enterobacteriaceae;g__Escherichia-Shigella;s__Escherichia_coli	d__Bacteria;p__Proteobacteria;c__Gammaproteobacteria;o__Enterobacterales;f__Enterobacteriaceae;g__Escherichia-Shigella	387	0.55
9a38446a8cba2f11f974a622d8092fb1	3042186b85304e8d_1	98.913	10124	10306	V5V6	Clostridium_beijerinckii_ATCC_35702	True	d__Bacteria;p__Firmicutes;c__Clostridia;o__Clostridiales;f__Clostridiaceae;g__Clostridium_sensu_stricto_1;s__uncultured_Clostridium	d__Bacteria;p__Firmicutes;c__Clostridia;o__Clostridiales;f__Clostridiaceae;g__Clostridium_sensu_stricto_1	10	0.01
91c2afa196dfa8a99f1dda3f89470f7d	2504aeb524e74d25_1	100	230126	230349	V5V6	Porphyromonas_gingivalis_ATCC_33277	True			61	0.09
28b80793effa80e95f17ba78f86ba3a1	5964adb8d0df4fde_1	100	478287	478487	V5V6	Lactobacillus_gasseri_ATCC_33323	True			105	0.15
8eead67fce32a188c8e0264c8c648a2d	4762fdd895094939_1	100	97140	97366	V5V6	Helicobacter_pylori_ATCC_700392	True	d__Bacteria;p__Campilobacterota;c__Campylobacteria;o__Campylobacterales;f__Helicobacteraceae;g__uncult	d__Bacteria;p__Campilobacterota;c__Campylobacteria;o__Campylobacterales;f__Helicobacteraceae;g__uncultured	116	0.17

								ured;s__uncultured_Epsilonproteobacteria			
fce4db9d80643391cfdf2508ac33fa8d	2b7fa0e1de5f42de_1	97.207	183304	183482	V5V6	Deinococcus_radiodurans_ATCC_BAA_816	True	d__Bacteria;p__Firmicutes;c__Clostridia;o__Clostridiales;f__Clostridiaceae;g__Clostridium_sensu_stricto_13;s__rumen_bacterium	d__Bacteria;p__Firmicutes;c__Clostridia;o__Clostridiales;f__Clostridiaceae;g__Clostridium_sensu_stricto_13	33	0.05
0fa7e151a8f8896c85778ba8addc5103	4762fdd895094939_1	98.37	97140	97322	V5V6	Helicobacter_pylori_ATCC_700392	True			15	0.02
f2497fa105c39edd688f04e4a8bb515a	adb6d513b7374f84_1	100	17506	17718	V5V6	Streptococcus_mutans_ATCC_700610	True	d__Bacteria;p__Firmicutes;c__Bacilli;o__Lactobacillales;f__Streptococcaceae;g__Streptococcus;s__Streptococcus_gordonii	d__Bacteria;p__Firmicutes;c__Bacilli;o__Lactobacillales;f__Streptococcaceae;g__Streptococcus	423	0.60
e6b816d934aea16c3b7dd7e8b25a37d0	5371a9904eb64361_1	98.953	9725	9915	V5V6	Bacillus_pacificus_ATCC_10987	True		d__Bacteria;p__Firmicutes;c__Bacilli;o__Bacillales;f__Bacillaceae;g__Bacillus	157	0.22
5ea873145571ae5cc0af20f573bae47f	5964adb8d0df4fde_1	98.37	478287	478470	V5V6	Lactobacillus_gasseri_ATCC_33323	True	d__Bacteria;p__Firmicutes;c__Bacilli;o__Lactobacillales;f__Enterococcaceae;g__Enterococcus;s__Enterococcus_mundtii	d__Bacteria;p__Firmicutes;c__Bacilli;o__Lactobacillales;f__Enterococcaceae;g__Enterococcus	104	0.15
ddf00153358dd52bbc1a5646ff83ff19	c45a1c57adf4460e_2	100	23891	24087	V5V6	Bifidobacterium_adolescentis_ATCC_15703	True	d__Bacteria;p__Actinobacteriota;c__Actinobacteria;o__Bifidobacteriales;f__Bifidobacteriaceae;g__Bifidobacterium;s__Bifidobacterium_sp.	d__Bacteria;p__Actinobacteriota;c__Actinobacteria;o__Bifidobacteriales;f__Bifidobacteriaceae;g__Bifidobacterium	1212	1.73
ab2cd68e1a2959f94c31e0a25fcf6b10	285a115d88c043f1_1	100	679151	679370	V5V6	Pseudomonas_aeruginosa_ATCC_9027	True	d__Bacteria;p__Proteobacteria;c__Gammaproteobacteria;o__Pseudomonadales;f__Pseudomonadaceae;g__Pseudomonas;s__Pseudomonas_aeruginosa	d__Bacteria;p__Proteobacteria;c__Gammaproteobacteria;o__Pseudomonadales;f__Pseudomonadaceae;g__Pseudomonas	136	0.19
f32884e8124f8ed14a6a973f9e59b65f	d889f9dd971b473d_1	100	347420	347221	V5V6	Phocaeicola_vulgatus_ATCC_8482	True	d__Bacteria;p__Bacteroidota;c__Bacteroidia;o__Bacteroidales;f__Paludibacteraceae;g__Paludibacter;s__uncultured_bacterium	d__Bacteria;p__Bacteroidota;c__Bacteroidia;o__Bacteroidales;f__Paludibacteraceae;g__Paludibacter	7810	11.14
e9de1e3fc5a26f227056f0678cdec4df	d889f9dd971b473d_1	99.5	347420	347221	V5V6	Phocaeicola_vulgatus_ATCC_8482	True	d__Bacteria;p__Bacteroidota;c__Bacteroidia;o__Bacteroidales;f__Paludibacteraceae;g__Paludibacter;s__uncultured_bacterium	d__Bacteria;p__Bacteroidota;c__Bacteroidia;o__Bacteroidales;f__Paludibacteraceae;g__Paludibacter	5106	7.28
f9050ac7af24954bcf69dd15c8a47e64	27b35dd1651e48dd_1	98.37	214197	214380	V5V6	Enterococcus_faecalis	True		d__Bacteria;p__Firmicutes;c__Bacilli;o__Lactobacillales;f__Enterococcaceae;g__Enterococcus	73	0.10

						_ATCC_47077					
0dfd270b9832257283af9b9f78acc52b	1eb3021faaec49c2_1	98.585	455783	455994	V5V6	Escherichia_coli_ATCC_700926	True	d__Bacteria;p__Proteobacteria;c__Gammaproteobacteria;o__Enterobacterales;f__Enterobacteriaceae;g__Escherichia-Shigella;s__Escherichia_coli	d__Bacteria;p__Proteobacteria;c__Gammaproteobacteria;o__Enterobacterales;f__Enterobacteriaceae;g__Escherichia-Shigella	45	0.06
2de4f95c844ab13c180b39c7568462a0					V5V6			d__Bacteria;p__Firmicutes;c__Clostridia;o__Clostridiales;f__Clostridiaceae;g__Clostridium_sensu_stricto_13;s__rumen_bacterium	d__Bacteria;p__Firmicutes;c__Clostridia;o__Clostridiales;f__Clostridiaceae;g__Clostridium_sensu_stricto_13	83	0.12
928d3dc22f242a87f28f9219c099c075					V5V6			d__Bacteria;p__Proteobacteria;c__Alphaproteobacteria;o__Rhodobacterales;f__Rhodobacteraceae;g__Paracoccus;s__uncultured_Paracoccus	d__Bacteria;p__Proteobacteria;c__Alphaproteobacteria;o__Rhodobacterales;f__Rhodobacteraceae;g__Paracoccus	19	0.03
8046b007682c31562cd6f016415cb45f					V5V6					12	0.02
c42e0b7c52736f3bd883bf490b386e9e					V5V6				d__Bacteria;p__Proteobacteria;c__Gammaproteobacteria;o__Enterobacterales;f__Enterobacteriaceae;g__Escherichia-Shigella	13	0.02
634f27f65e3b8ecc24f659647f103077					V5V6			d__Bacteria;p__Proteobacteria;c__Gammaproteobacteria;o__Pseudomonadales;f__Moraxellaceae;g__Acinetobacter;s__Acinetobacter_sp.	d__Bacteria;p__Proteobacteria;c__Gammaproteobacteria;o__Pseudomonadales;f__Moraxellaceae;g__Acinetobacter	6	0.01
81feba462792be505fe7ad9288c6616c					V5V6					15	0.02
c79e84621eddb90c282e75399d80fe76					V5V6			d__Bacteria;p__Firmicutes;c__Bacilli;o__Lactobacillales;f__Streptococcaceae;g__Streptococcus;s__Streptococcus_gordonii	d__Bacteria;p__Firmicutes;c__Bacilli;o__Lactobacillales;f__Streptococcaceae;g__Streptococcus	7	0.01
fbfec8c1653111db7b8f9a5acf06e0e4					V5V6				d__Bacteria;p__Proteobacteria;c__Gammaproteobacteria;o__Burkholderiales;f__Oxalobacteraceae;g__Herbaspirillum	2	0.00
3f52d098be6c09dc2688c232e0522e6e					V5V6					74	0.11
e3007d82e148295473b573cb0cfd1802					V5V6					22	0.03

ea6cfdf2f886a465e1f22845677ca43e					V5V6					6	0.01
678715dd463dd71ad724f2490dfd7364					V5V6					16	0.02
a7468bfe80697ea6b0e8655125ffbec9					V5V6			d__Bacteria;p__Firmicutes;c__Bacilli;o__Lactobacillales;f__Enterococcaceae;g__Enterococcus;s__Enterococcus_mundtii	d__Bacteria;p__Firmicutes;c__Bacilli;o__Lactobacillales;f__Enterococcaceae;g__Enterococcus	90	0.13
4bf0aeed2b0e74b2bc4e2447e45fa6cf					V5V6				d__Bacteria;p__Firmicutes;c__Bacilli;o__Staphylococcales;f__Staphylococcaceae;g__Staphylococcus	12	0.02
105b900b8b271fc1279a9f00f63fd8da					V5V6			d__Bacteria;p__Firmicutes;c__Bacilli;o__Lactobacillales;f__Streptococcaceae;g__Streptococcus;s__Streptococcus_gordonii	d__Bacteria;p__Firmicutes;c__Bacilli;o__Lactobacillales;f__Streptococcaceae;g__Streptococcus	128	0.18
6beaac5c0c09537786551f0740e1f8e6	38ad2dab3e5e43b7_1	100	628519	628718	V5V6	Staphylococcus_aureus_subsp_aureus_ATCC_BAA_1556	True		d__Bacteria;p__Firmicutes;c__Bacilli;o__Staphylococcales;f__Staphylococcaceae;g__Staphylococcus	373	0.53
2d171c4fcb88172f9022e70d09b64578					V5V6			d__Bacteria;p__Firmicutes;c__Clostridia;o__Clostridiales;f__Clostridiaceae;g__Clostridium_sensu_stricto_1;s__uncultured_Clostridium	d__Bacteria;p__Firmicutes;c__Clostridia;o__Clostridiales;f__Clostridiaceae;g__Clostridium_sensu_stricto_1	25	0.04
9356c352f9e2de6d1675a9a73c9608b6					V5V6			d__Bacteria;p__Firmicutes;c__Clostridia;o__Clostridiales;f__Clostridiaceae;g__Clostridium_sensu_stricto_1;s__uncultured_Clostridium	d__Bacteria;p__Firmicutes;c__Clostridia;o__Clostridiales;f__Clostridiaceae;g__Clostridium_sensu_stricto_1	60	0.09
3b08e2713ab41b7a404cfaa9ad2e833a					V5V6			d__Bacteria;p__Firmicutes;c__Bacilli;o__Lactobacillales;f__Streptococcaceae;g__Streptococcus;s__Streptococcus_gordonii	d__Bacteria;p__Firmicutes;c__Bacilli;o__Lactobacillales;f__Streptococcaceae;g__Streptococcus	1	0.00
792bf390207b1d9bc70595633c70b17f					V5V6			d__Bacteria;p__Proteobacteria;c__Alphaproteobacteria;o__Rhodobacterales;f__Rhodobacteraceae;g__Paracoccus;s__uncultured_Paracoccus	d__Bacteria;p__Proteobacteria;c__Alphaproteobacteria;o__Rhodobacterales;f__Rhodobacteraceae;g__Paracoccus	18	0.03
e25ebbf67c652da38afef831ff1bda3f					V5V6			d__Bacteria;p__Proteobacteria;c__Alphaproteobacteria;o__Rhodobacterales;f__Rhodobacteraceae;g__Paracoccus;s__uncultured_Paracoccus	d__Bacteria;p__Proteobacteria;c__Alphaproteobacteria;o__Rhodobacterales;f__Rhodobacteraceae;g__Paracoccus	35	0.05

56f1751149e7f17b825efca9b0ec1569					V5V6			d__Bacteria;p__Proteobacteria;c__Alphaproteobacteria;o__Rhizobiales;f__Rhizobiaceae;g__Mesorhizobium;s__Mesorhizobium_sp.	d__Bacteria;p__Proteobacteria;c__Alphaproteobacteria;o__Rhizobiales;f__Rhizobiaceae;g__Mesorhizobium	6	0.01
5903cb99ef12f590c6c3de7e367df82d					V5V6					1	0.00
c6c3ab4e828fb40d6e05967b7aac9338					V5V6					93	0.13
c81def64b8f2523ef3c7fefc8711edf6					V5V6			d__Bacteria;p__Firmicutes;c__Bacilli;o__Lactobacillales;f__Streptococcaceae;g__Streptococcus;s__Streptococcus_gordonii	d__Bacteria;p__Firmicutes;c__Bacilli;o__Lactobacillales;f__Streptococcaceae;g__Streptococcus	11	0.02
ef3199891723da4b0a64b80682bada68					V5V6					2	0.00
d77aa435264c499b17b7e0eeb121a585	27b35dd1651e48dd_1	100	214197	214396	V5V6	Enterococcus_faecalis_ATCC_47077	True	d__Bacteria;p__Firmicutes;c__Bacilli;o__Lactobacillales;f__Enterococcaceae;g__Enterococcus;s__Enterococcus_faecalis	d__Bacteria;p__Firmicutes;c__Bacilli;o__Lactobacillales;f__Enterococcaceae;g__Enterococcus	123	0.18
20392706f8152cfe29c2fe551e5c408d	2b7fa0e1de5f42de_1	98.324	183304	183482	V5V6	Deinococcus_radiodurans_ATCC_BAA_816	True			20	0.03
5a6c87d6a4eb5e114959f6192f29b641	c45a1c57adf4460e_2	100	23609	23861	V5V6	Bifidobacterium_adolescentis_ATCC_15703	True			30	0.04
d087f1ce2b06f0b0deb360e308d878c1	045df7c7cb0f42b5_1	100	1222723	1222504	V5V6	Neisseria_meningitidis_ATCC_BAA_335	True	d__Bacteria;p__Proteobacteria;c__Gammaproteobacteria;o__Burkholderiales;f__Chromobacteriaceae;g__Vogesella;s__uncultured_bacterium	d__Bacteria;p__Proteobacteria;c__Gammaproteobacteria;o__Burkholderiales;f__Chromobacteriaceae;g__Vogesella	114	0.16
421770193803d8f3fe9cf981628b1cde	1eb3021faaec49c2_1	100	455528	455753	V4	Escherichia_coli_ATCC_700926	True		d__Bacteria;p__Proteobacteria;c__Gammaproteobacteria;o__Enterobacterales;f__Enterobacteriaceae;g__Escherichia-Shigella	3	0.00
ab6b7b90747341ce39f82d0a22a1d37d					V4				d__Bacteria;p__Proteobacteria;c__Alphaproteobacteria;o__Rhizobiales;f__Beijerinckiaceae;g__Methylobacterium-Methylorubrum	4	0.00

a0a0b67a4c65c6778fe8e1e04e7e3d49	V4	d__Bacteria;p__Firmicutes;c__Clostridia;o__Peptostreptococcales-Tissierellales;f__Peptostreptococcaceae;g__Clostridioides;s__Clostridioides_difficile	d__Bacteria;p__Firmicutes;c__Clostridia;o__Peptostreptococcales-Tissierellales;f__Peptostreptococcaceae;g__Clostridioides	9	0.00
efe485bbdd633d28fa7694f38d03608f	V4			2	0.00
8375d98f345343540e7a4d73b2eb1f82	V4		d__Bacteria;p__Firmicutes;c__Clostridia;o__Clostridiales;f__Clostridiaceae;g__Clostridium_sensu_stricto_1	6	0.00
3481fa43fe5fba6aecdc7f9aae6ed9c0	V4			160	0.07
4cbfff144d4e7a4e0f4619ed505be070	V4			55	0.02
f5f5e0da89730462abaf6301a9557193	V4		d__Bacteria;p__Firmicutes;c__Clostridia;o__Oscillospirales;f__Ruminococcaceae;g__Faecalibacterium	20	0.01
fb428d07d03e32be0b85eacbed10df0a	V4	d__Bacteria;p__Firmicutes;c__Clostridia;o__Lachnospirales;f__Lachnospiraceae;g__Coprococcus;s__Coprococcus_eutactus	d__Bacteria;p__Firmicutes;c__Clostridia;o__Lachnospirales;f__Lachnospiraceae;g__Coprococcus	19	0.01
ec6732c2e0d4cf64b3d0350e7fe3defb	V4		d__Bacteria;p__Firmicutes;c__Clostridia;o__Lachnospirales;f__Lachnospiraceae;g__Roseburia	41	0.02
247dd7bf2820990e45df15e2ca76dbd1	V4			1	0.00
c939ee112ba982665e012437dae05c1e	V4			186	0.08
2c982937754e6321f861027032db80f7	V4		d__Bacteria;p__Bacteroidota;c__Bacteroidia;o__Bacteroidales;f__Bacteroidaceae;g__Bacteroides	13	0.01
35cbb315e019e0a8768d4b73862df415	V4		d__Bacteria;p__Actinobacteriota;c__Coriobacteriia;o__Coriobacteriales;f__Coriobacteriaceae;g__Collinsella	11	0.00
9c754ee0918899602c8196cfc5a75692	V4			6	0.00

685a1812a6e46f72d7cf1f4a060b8e96					V4				d__Bacteria;p__Actinobacteriota;c__Actinobacteria;o__Corynebacteriales;f__Corynebacteriaceae;g__Corynebacterium	3	0.00
99bccd58aaeb00f98e22add40be6e36b					V4			d__Bacteria;p__Firmicutes;c__Clostridia;o__Peptostreptococcales-Tissierellales;f__Peptostreptococcaceae;g__Clostridioides;s__Clostridioides_difficile	d__Bacteria;p__Firmicutes;c__Clostridia;o__Peptostreptococcales-Tissierellales;f__Peptostreptococcaceae;g__Clostridioides	408	0.17
fa531a9e5007f7d080e555401270d85a					V4				d__Bacteria;p__Fusobacteriota;c__Fusobacteriia;o__Fusobacteriales;f__Fusobacteriaceae;g__Fusobacterium	105	0.04
cd3526e9ddf608ed31c4f3d648ca8034					V4				d__Bacteria;p__Proteobacteria;c__Gammaproteobacteria;o__Burkholderiales;f__Sutterellaceae;g__Parasutterella	6	0.00
a19be9fe0ba2ea284bd53cb94f622471					V4				d__Bacteria;p__Firmicutes;c__Clostridia;o__Clostridiales;f__Clostridiaceae;g__Clostridium_sensu_stricto_1	2	0.00
5a6c87d6a4eb5e114959f6192f29b641	c45a1c57adf4460e_2	100	23609	23861	V4	Bifidobacterium_adolescentis_ATCC_15703	True		d__Bacteria;p__Actinobacteriota;c__Actinobacteria;o__Bifidobacteriales;f__Bifidobacteriaceae;g__Bifidobacterium	9848	4.17
8df399e29acb5a583f449a82db4e17c9					V4				d__Bacteria;p__Firmicutes;c__Bacilli;o__Lactobacillales;f__Lactobacillaceae;g__Lactobacillus	46	0.02
da5bc53279a680c25d503cb1bdc0e57a	9dc637a985b042c4_1	100	605730	605977	V4	Cutibacterium_acnes_ATCC_11828	True		d__Bacteria;p__Actinobacteriota;c__Actinobacteria;o__Propionibacteriales;f__Propionibacteriaceae;g__Cutibacterium	3097	1.31
463d246ab2d6bc2481d5aab8de075a8b	9dc637a985b042c4_1	100	605754	605977	V4	Cutibacterium_acnes_ATCC_11828	True		d__Bacteria;p__Actinobacteriota;c__Actinobacteria;o__Propionibacteriales;f__Propionibacteriaceae;g__Cutibacterium	3	0.00
e7cfd084265c4df4856ca07b1c9b24ee	adb6d513b7374f84_1	100	17225	17476	V4	Streptococcus_mutans_ATCC_700610	True	d__Bacteria;p__Firmicutes;c__Bacilli;o__Lactobacillales;f__Streptococcaceae;g__Streptococcus;s__Streptococcus_mutans	d__Bacteria;p__Firmicutes;c__Bacilli;o__Lactobacillales;f__Streptococcaceae;g__Streptococcus	10661	4.52
65d43491988bfe557da4d86a5ba25dae	f5bcb58692924cb7_1	100	1597467	1597215	V4	Staphylococcus_epidermidis_ATCC_12228	True		d__Bacteria;p__Firmicutes;c__Bacilli;o__Staphylococcales;f__Staphylococcaceae;g__Staphylococcus	27015	11.45

e3055f1b3a2ef5ffe239567f02e0e758	58ae911973b74ffc_1	100	16843	17094	V4	Streptococcus_agalactiae_ATCC_BAA_611	True	d__Bacteria;p__Firmicutes;c__Bacilli;o__Lactobacillales;f__Streptococcaceae;g__Streptococcus;s__Streptococcus_agalactiae	d__Bacteria;p__Firmicutes;c__Bacilli;o__Lactobacillales;f__Streptococcaceae;g__Streptococcus	13110	5.55
e832be098a5318684958d14305267752	4762fdd895094939_1	100	96857	97110	V4	Helicobacter_pylori_ATCC_700392	True	d__Bacteria;p__Campilobacterota;c__Campylobacteria;o__Campylobacterales;f__Helicobacteraceae;g__Helicobacter;s__Helicobacter_pylori	d__Bacteria;p__Campilobacterota;c__Campylobacteria;o__Campylobacterales;f__Helicobacteraceae;g__Helicobacter	5943	2.52
9fd9ac1df429568b5d1ca6aa0b9f0b34	071a82bb90b94c6f_1	99.213	18741	18993	V4	Acinetobacter_baumannii_ATCC_17978	True		d__Bacteria;p__Proteobacteria;c__Gammaproteobacteria;o__Pseudomonadales;f__Moraxellaceae;g__Acinetobacter	16	0.01
df8456a1abbfb4c8a2c450b44378d4cb	89e35fec14744942_1	100	528830	529082	V4	Schaalia_odontolytica_ATCC_17982	True	d__Bacteria;p__Actinobacteriota;c__Actinobacteria;o__Actinomycetales;f__Actinomycetaceae;g__Actinomyces;s__Schaalia_odontolytica	d__Bacteria;p__Actinobacteriota;c__Actinobacteria;o__Actinomycetales;f__Actinomycetaceae;g__Actinomyces	4128	1.75
cb97fb83d4c8cc6eccded352a4ca3f8f	3042186b85304e8d_1	99.605	9842	10094	V4	Clostridium_beijerinckii_ATCC_35702	True	d__Bacteria;p__Firmicutes;c__Clostridia;o__Clostridiales;f__Clostridiaceae;g__Clostridium_sensu_stricto_1;s__Clostridium_beijerinckii	d__Bacteria;p__Firmicutes;c__Clostridia;o__Clostridiales;f__Clostridiaceae;g__Clostridium_sensu_stricto_1	1954	0.83
ff9d93d7b7e46787568f2d241caeaf3b	285a115d88c043f1_1	100	678869	679121	V4	Pseudomonas_aeruginosa_ATCC_9027	True		d__Bacteria;p__Proteobacteria;c__Gammaproteobacteria;o__Pseudomonadales;f__Pseudomonadaceae;g__Pseudomonas	5608	2.38
2385fe1c2dd5a3f83237272a6644088b	2b7fa0e1de5f42de_1	100	183022	183274	V4	Deinococcus_radiodurans_ATCC_BAA_816	True	d__Bacteria;p__Deinococcota;c__Deinococci;o__Deinococcales;f__Deinococcaceae;g__Deinococcus;s__Deinococcus_radiodurans	d__Bacteria;p__Deinococcota;c__Deinococci;o__Deinococcales;f__Deinococcaceae;g__Deinococcus	9108	3.86
bdf8a26094624622d68509a87fa75ba7	5371a9904eb64361_1	100	9443	9695	V4	Bacillus_pacificus_ATCC_10987	True		d__Bacteria;p__Firmicutes;c__Bacilli;o__Bacillales;f__Bacillaceae;g__Bacillus	23458	9.94
e21e804c8cb49a5e0492b8fc5454220f	2504aeb524e74d25_1	100	229844	230096	V4	Porphyromonas_gingivalis_ATCC_33277	True	d__Bacteria;p__Bacteroidota;c__Bacteroidia;o__Bacteroidales;f__Porphyromonadaceae;g__Porphyromonas;s__Porphyromonas_gingivalis	d__Bacteria;p__Bacteroidota;c__Bacteroidia;o__Bacteroidales;f__Porphyromonadaceae;g__Porphyromonas	8004	3.39
dffc86cefa76e3e3d93e7eea450e6807	266a835027f7464b_2	100	38074	37822	V4	Rhodobacter_sphaeroides_ATCC_17029	True		d__Bacteria;p__Proteobacteria;c__Alphaproteobacteria;o__Rhodobacterales;f__Rhodobacteraceae;g__Rhodobacter	3076	1.30

b2062ae94fb00a931014b67b499295a8	d889f9dd971b473d_1	99.605	347702	347450	V4	Phocaeicola_vulgatus_ATCC_8482	True	d__Bacteria;p__Bacteroidota;c__Bacteroidia;o__Bacteroidales;f__Bacteroidaceae;g__Bacteroides;s__Bacteroides_vulgatus	d__Bacteria;p__Bacteroidota;c__Bacteroidia;o__Bacteroidales;f__Bacteroidaceae;g__Bacteroides	2269	0.96
1d19b18db48daa1837d18d3171334ded	f5bcb58692924cb7_1	99.605	1597467	1597215	V4	Staphylococcus_epidermidis_ATCC_12228	True		d__Bacteria;p__Firmicutes;c__Bacilli;o__Staphylococcales;f__Staphylococcaceae;g__Staphylococcus	2051	0.87
096e60ea01b8425c20593919d66c37d2	5371a9904eb64361_1	98.819	9443	9695	V4	Bacillus_pacificus_ATCC_10987	True		d__Bacteria;p__Firmicutes;c__Bacilli;o__Bacillales;f__Bacillaceae;g__Bacillus	3	0.00
99deb3c5ecb022ec05609ebd1112a557	d889f9dd971b473d_1	100	347702	347450	V4	Phocaeicola_vulgatus_ATCC_8482	True	d__Bacteria;p__Bacteroidota;c__Bacteroidia;o__Bacteroidales;f__Bacteroidaceae;g__Bacteroides;s__Bacteroides_vulgatus	d__Bacteria;p__Bacteroidota;c__Bacteroidia;o__Bacteroidales;f__Bacteroidaceae;g__Bacteroides	11308	4.79
ea403646ed22d679fa4586263d8fc32f	071a82bb90b94c6f_1	100	18741	18993	V4	Acinetobacter_baumannii_ATCC_17978	True		d__Bacteria;p__Proteobacteria;c__Gammaproteobacteria;o__Pseudomonadales;f__Moraxellaceae;g__Acinetobacter	16954	7.18
4e8d7a4662640b90817f015280cf5713	3042186b85304e8d_1	100	9842	10094	V4	Clostridium_beijerinckii_ATCC_35702	True		d__Bacteria;p__Firmicutes;c__Clostridia;o__Clostridiales;f__Clostridiaceae;g__Clostridium_sensu_stricto_1	30566	12.95
8001826dafd338cf078dd28c2eae05ed	d889f9dd971b473d_1	99.605	347702	347450	V4	Phocaeicola_vulgatus_ATCC_8482	True	d__Bacteria;p__Bacteroidota;c__Bacteroidia;o__Bacteroidales;f__Bacteroidaceae;g__Bacteroides;s__Bacteroides_vulgatus	d__Bacteria;p__Bacteroidota;c__Bacteroidia;o__Bacteroidales;f__Bacteroidaceae;g__Bacteroides	2140	0.91
9908fffab7ed4f3bec44cda2f5084d49	27b35dd1651e48dd_1	100	213915	214167	V4	Enterococcus_faecalis_ATCC_47077	True		d__Bacteria;p__Firmicutes;c__Bacilli;o__Lactobacillales;f__Enterococcaceae;g__Enterococcus	8450	3.58
8224351b2abd16dd4d58c3015ff5e795	045df7c7cb0f42b5_1	100	1223005	1222753	V4	Neisseria_meningitidis_ATCC_BAA_335	True	d__Bacteria;p__Proteobacteria;c__Gammaproteobacteria;o__Burkholderiales;f__Neisseriaceae;g__Neisseria;s__Neisseria_meningitidis	d__Bacteria;p__Proteobacteria;c__Gammaproteobacteria;o__Burkholderiales;f__Neisseriaceae;g__Neisseria	9076	3.85
d46e2205f0c6ecf67b51f83d111c509c	1eb3021faaec49c2_1	100	455501	455753	V4	Escherichia_coli_ATCC_700926	True		d__Bacteria;p__Proteobacteria;c__Gammaproteobacteria;o__Enterobacterales;f__Enterobacteriaceae;g__Escherichia-Shigella	13582	5.75
0df6c802966e8670279671824da4f10a	5964adb8d0df4fde_1	100	478005	478257	V4	Lactobacillus_gasseri	True		d__Bacteria;p__Firmicutes;c__Bacilli;o__Lactobacillales;f__Lactobacillaceae;g__Lactobacillus	13490	5.72

		_ATCC_33323				
EU776082.1.1401	Multiplex		d__Bacteria;p__Proteobacteria;c__Gammaproteobacteria;o__Enterobacterales;f__Enterobacteriaceae;g__Escherichia-Shigella;s__uncultured_bacterium	d__Bacteria;p__Proteobacteria;c__Gammaproteobacteria;o__Enterobacterales;f__Enterobacteriaceae;g__Escherichia-Shigella	3	0.00
MF629745.1.1219	Multiplex		d__Bacteria;p__Proteobacteria;c__Gammaproteobacteria;o__Enterobacterales;f__Enterobacteriaceae;g__Enterobacter;s__Enterobacter_cloacae	d__Bacteria;p__Proteobacteria;c__Gammaproteobacteria;o__Enterobacterales;f__Enterobacteriaceae;g__Enterobacter	6	0.01
AF349929.1.1503	Multiplex		d__Bacteria;p__Firmicutes;c__Bacilli;o__Lactobacillales;f__Enterococcaceae;g__Enterococcus;s__Enterococcus_sp.	d__Bacteria;p__Firmicutes;c__Bacilli;o__Lactobacillales;f__Enterococcaceae;g__Enterococcus	18	0.02
CP020424.2046841.204	Multiplex				4	0.00
EU511507.1.1388_005	Multiplex				1	0.00
DQ809036.1.1400_002	Multiplex				10.02	0.01
JX426120.1.1502	Multiplex		d__Bacteria;p__Firmicutes;c__Bacilli;o__Lactobacillales;f__Lactobacillaceae;g__Lactobacillus;s__Lactobacillus_plantarum	d__Bacteria;p__Firmicutes;c__Bacilli;o__Lactobacillales;f__Lactobacillaceae;g__Lactobacillus	6	0.01
CP019858.4077753.407	Multiplex				3	0.00
CP012650.1579871.158	Multiplex				6	0.01
KC584759.1.1459	Multiplex		d__Bacteria;p__Proteobacteria;c__Gammaproteobacteria;o__Enterobacterales;f__Enterobacteriaceae;g__Klebsiella;s__Klebsiella_sp.	d__Bacteria;p__Proteobacteria;c__Gammaproteobacteria;o__Enterobacterales;f__Enterobacteriaceae;g__Klebsiella	2	0.00
JN013122.1.1460_053	Multiplex				1	0.00
JF772072.1.1504	Multiplex		d__Bacteria;p__Proteobacteria;c__Gammaproteobacteria;o__Enterobacterales;f__Enterobacteriaceae;g__Klebsiella;s__Klebsiella_oxytoca	d__Bacteria;p__Proteobacteria;c__Gammaproteobacteria;o__Enterobacterales;f__Enterobacteriaceae;g__Klebsiella	2	0.00
MF369967.1.1401	Multiplex		d__Bacteria;p__Firmicutes;c__Bacilli;o__Lactobacillales;f__Enterococcaceae;g__Enterococcus;s__Enterococcus_faecium	d__Bacteria;p__Firmicutes;c__Bacilli;o__Lactobacillales;f__Enterococcaceae;g__Enterococcus	2	0.00

EU776683.1.1429	Multiplex	d__Bacteria;p__Firmicutes;c__Bacilli;o__Lactobacillales;f__Lactobacillaceae;g__Lactobacillus;s__uncultured_bacterium	d__Bacteria;p__Firmicutes;c__Bacilli;o__Lactobacillales;f__Lactobacillaceae;g__Lactobacillus	19	0.02
KY228992.1.1320	Multiplex	d__Bacteria;p__Firmicutes;c__Bacilli;o__Lactobacillales;f__Lactobacillaceae;g__Lactobacillus;s__Lactobacillus_sp.	d__Bacteria;p__Firmicutes;c__Bacilli;o__Lactobacillales;f__Lactobacillaceae;g__Lactobacillus	1	0.00
LFDY01000027.124228.	Multiplex			11	0.01
DQ057465.1.1471_001	Multiplex			3.19	0.00
DQ823798.1.1415	Multiplex	d__Bacteria;p__Bacteroidota;c__Bacteroidia;o__Bacteroidales;f__Muribaculaceae;g__Muribaculaceae;s__uncultured_bacterium	d__Bacteria;p__Bacteroidota;c__Bacteroidia;o__Bacteroidales;f__Muribaculaceae;g__Muribaculaceae	3	0.00
EU776602.1.1302	Multiplex	d__Bacteria;p__Firmicutes;c__Clostridia;o__Peptostreptococcales-Tissierellales;f__Peptostreptococcaceae;g__Terrisporobacter;s__uncultured_bacterium	d__Bacteria;p__Firmicutes;c__Clostridia;o__Peptostreptococcales-Tissierellales;f__Peptostreptococcaceae;g__Terrisporobacter	25	0.03
CP019858.1093194.109	Multiplex			7	0.01
AAML04000003.230439.	Multiplex			4	0.00
KM819125.1.1527	Multiplex	d__Bacteria;p__Firmicutes;c__Bacilli;o__Lactobacillales;f__Lactobacillaceae;g__Lactobacillus;s__uncultured_Bacillus	d__Bacteria;p__Firmicutes;c__Bacilli;o__Lactobacillales;f__Lactobacillaceae;g__Lactobacillus	1	0.00
EU772368.1.1408	Multiplex	d__Bacteria;p__Firmicutes;c__Bacilli;o__Lactobacillales;f__Leuconostocaceae;g__Leuconostoc;s__uncultured_bacterium	d__Bacteria;p__Firmicutes;c__Bacilli;o__Lactobacillales;f__Leuconostocaceae;g__Leuconostoc	4	0.00
DQ002385.1.1486_001	Multiplex			12.29	0.01
EF096405.1.1378_011	Multiplex			2.35	0.00
EF406388.1.1504_077	Multiplex			1.99	0.00
EF097505.1.1302	Multiplex	d__Bacteria;p__Actinobacteriota;c__Coriobacteriia;o__Coriobacteriales;f__Atopobiaceae;g__Coriobacteriaceae_UCG-002;s__uncultured_bacterium	d__Bacteria;p__Actinobacteriota;c__Coriobacteriia;o__Coriobacteriales;f__Atopobiaceae;g__Coriobacteriaceae_UCG-002	1	0.00

EU467051.1.1321	Multiplex	d__Bacteria;p__Firmicutes;c__Clostridia;o__Clostridiales;f__Clostridiaceae;g__Clostridium_sensu_stricto_1;s__uncultured_bacterium	d__Bacteria;p__Firmicutes;c__Clostridia;o__Clostridiales;f__Clostridiaceae;g__Clostridium_sensu_stricto_1	2	0.00
GQ416526.1.1465_001	Multiplex			0.41	0.00
AM286415.17506.18994_001	Multiplex			7235.31	8.45
GQ448270.1.1398	Multiplex	d__Bacteria;p__Bacteroidota;c__Bacteroidia;o__Bacteroidales;f__Prevotellaceae;g__Prevotella;s__uncultured_bacterium	d__Bacteria;p__Bacteroidota;c__Bacteroidia;o__Bacteroidales;f__Prevotellaceae;g__Prevotella	7	0.01
CP015126.1170520.117	Multiplex			7	0.01
FN668375.4306350.430	Multiplex			130	0.15
KP641129.1.1470	Multiplex	d__Bacteria;p__Proteobacteria;c__Gammaproteobacteria;o__Enterobacterales;f__Enterobacteriaceae;g__Escherichia-Shigella;s__uncultured_bacterium	d__Bacteria;p__Proteobacteria;c__Gammaproteobacteria;o__Enterobacterales;f__Enterobacteriaceae;g__Escherichia-Shigella	4	0.00
EU136678.1.2011_001	Multiplex			4984.34	5.82
JQ471932.1.1385	Multiplex	d__Bacteria;p__Fusobacteriota;c__Fusobacteriia;o__Fusobacteriales;f__Fusobacteriaceae;g__Fusobacterium;s__uncultured_bacterium	d__Bacteria;p__Fusobacteriota;c__Fusobacteriia;o__Fusobacteriales;f__Fusobacteriaceae;g__Fusobacterium	2	0.00
HQ745021.1.1418	Multiplex	d__Bacteria;p__Fusobacteriota;c__Fusobacteriia;o__Fusobacteriales;f__Fusobacteriaceae;g__Fusobacterium;s__uncultured_organism	d__Bacteria;p__Fusobacteriota;c__Fusobacteriia;o__Fusobacteriales;f__Fusobacteriaceae;g__Fusobacterium	46	0.05
CP018044.567281.5688	Multiplex			1	0.00
EU778419.1.1388	Multiplex	d__Bacteria;p__Proteobacteria;c__Gammaproteobacteria;o__Enterobacterales;f__Enterobacteriaceae;g__Escherichia-Shigella;s__uncultured_bacterium	d__Bacteria;p__Proteobacteria;c__Gammaproteobacteria;o__Enterobacterales;f__Enterobacteriaceae;g__Escherichia-Shigella	52	0.06
CP011118.356520.358068_001	Multiplex			183.07	0.21
CP026598.16203.17711	Multiplex	d__Bacteria;p__Firmicutes;c__Clostridia;o__Peptostreptococcales-Tissierellales;f__Peptostreptococcace	d__Bacteria;p__Firmicutes;c__Clostridia;o__Peptostreptococcales-	14	0.02

		ae;g__Clostridioides;s__Clostridioides_difficile	Tissierellales;f__Peptostreptococcaceae;g__Clostridioides		
EU451868.1.1386_001	Multiplex			1	0.00
AJ289177.1.1394	Multiplex	d__Bacteria;p__Fusobacteriota;c__Fusobacteriia;o__Fusobacteriales;f__Fusobacteriaceae;g__Fusobacterium;s__uncultured_eubacterium	d__Bacteria;p__Fusobacteriota;c__Fusobacteriia;o__Fusobacteriales;f__Fusobacteriaceae;g__Fusobacterium	23	0.03
KF625184.1.1787	Multiplex	d__Bacteria;p__Proteobacteria;c__Gammaproteobacteria;o__Enterobacterales;f__Yersiniaceae;g__Serratia;s__Serratia_proteamaculans	d__Bacteria;p__Proteobacteria;c__Gammaproteobacteria;o__Enterobacterales;f__Yersiniaceae;g__Serratia	5	0.01
HQ798408.1.1428	Multiplex	d__Bacteria;p__Bacteroidota;c__Bacteroidia;o__Bacteroidales;f__Bacteroidaceae;g__Bacteroides;s__uncultured_organism	d__Bacteria;p__Bacteroidota;c__Bacteroidia;o__Bacteroidales;f__Bacteroidaceae;g__Bacteroides	1	0.00
HQ818413.1.1435	Multiplex	d__Bacteria;p__Bacteroidota;c__Bacteroidia;o__Bacteroidales;f__Bacteroidaceae;g__Bacteroides;s__uncultured_organism	d__Bacteria;p__Bacteroidota;c__Bacteroidia;o__Bacteroidales;f__Bacteroidaceae;g__Bacteroides	4	0.00
JQ477578.1.1386	Multiplex	d__Bacteria;p__Fusobacteriota;c__Fusobacteriia;o__Fusobacteriales;f__Fusobacteriaceae;g__Fusobacterium;s__uncultured_bacterium	d__Bacteria;p__Fusobacteriota;c__Fusobacteriia;o__Fusobacteriales;f__Fusobacteriaceae;g__Fusobacterium	4	0.00
KR027010.1.1568	Multiplex	d__Bacteria;p__Firmicutes;c__Bacilli;o__Lactobacillales;f__Streptococcaceae;g__Lactococcus;s__bacterium_NXKED9	d__Bacteria;p__Firmicutes;c__Bacilli;o__Lactobacillales;f__Streptococcaceae;g__Lactococcus	5	0.01
JX003602.1.1336	Multiplex	d__Bacteria;p__Firmicutes;c__Bacilli;o__Lactobacillales;f__Lactobacillaceae;g__Lactobacillus;s__Lactobacillus_plantarum	d__Bacteria;p__Firmicutes;c__Bacilli;o__Lactobacillales;f__Lactobacillaceae;g__Lactobacillus	15	0.02
CP015966.2805828.280	Multiplex			9	0.01
DQ801774.1.1401	Multiplex	d__Bacteria;p__Bacteroidota;c__Bacteroidia;o__Bacteroidales;f__Bacteroidaceae;g__Bacteroides;s__uncultured_bacterium	d__Bacteria;p__Bacteroidota;c__Bacteroidia;o__Bacteroidales;f__Bacteroidaceae;g__Bacteroides	6	0.01
MH899355.1.1236	Multiplex	d__Bacteria;p__Firmicutes;c__Bacilli;o__Lactobacillales;f__Lactobacillaceae;g__Lactobacillus;s__Lactobacillus_casei	d__Bacteria;p__Firmicutes;c__Bacilli;o__Lactobacillales;f__Lactobacillaceae;g__Lactobacillus	4	0.00
MUJV01000001.176.164	Multiplex			5	0.01

EU453007.1.1441	Multiplex	d__Bacteria;p__Firmicutes;c__Bacilli;o__Lactobacillales;f__Lactobacillaceae;g__Lactobacillus;s__uncultured_bacterium	d__Bacteria;p__Firmicutes;c__Bacilli;o__Lactobacillales;f__Lactobacillaceae;g__Lactobacillus	1	0.00
CP012323.1102504.110	Multiplex			41	0.05
JN559663.1.1511	Multiplex	d__Bacteria;p__Firmicutes;c__Clostridia;o__Lachnospirales;f__Lachnospiraceae;g__Lachnospiraceae_UCG-004;s__uncultured_bacterium	d__Bacteria;p__Firmicutes;c__Clostridia;o__Lachnospirales;f__Lachnospiraceae;g__Lachnospiraceae_UCG-004	19	0.02
DQ818682.1.1490	Multiplex	d__Bacteria;p__Firmicutes;c__Bacilli;o__Lactobacillales;f__Enterococcaceae;g__Enterococcus;s__uncultured_bacterium	d__Bacteria;p__Firmicutes;c__Bacilli;o__Lactobacillales;f__Enterococcaceae;g__Enterococcus	45	0.05
CP028529.16107.17614	Multiplex	d__Bacteria;p__Firmicutes;c__Clostridia;o__Peptostreptococcales-Tissierellales;f__Peptostreptococcaceae;g__Clostridioides;s__Clostridioides_difficile	d__Bacteria;p__Firmicutes;c__Clostridia;o__Peptostreptococcales-Tissierellales;f__Peptostreptococcaceae;g__Clostridioides	112	0.13
EU511242.1.1396_005	Multiplex			1	0.00
DQ808680.1.1386	Multiplex	d__Bacteria;p__Firmicutes;c__Clostridia;o__Oscillospirales;f__Ruminococcaceae;g__Faecalibacterium;s__uncultured_bacterium	d__Bacteria;p__Firmicutes;c__Clostridia;o__Oscillospirales;f__Ruminococcaceae;g__Faecalibacterium	17	0.02
DQ818104.1.1460	Multiplex	d__Bacteria;p__Firmicutes;c__Bacilli;o__Staphylococcales;f__Staphylococcaceae;g__Staphylococcus;s__uncultured_bacterium	d__Bacteria;p__Firmicutes;c__Bacilli;o__Staphylococcales;f__Staphylococcaceae;g__Staphylococcus	4	0.00
GQ156444.1.1349	Multiplex	d__Bacteria;p__Proteobacteria;c__Gammaproteobacteria;o__Enterobacterales;f__Enterobacteriaceae;g__Escherichia-Shigella;s__uncultured_bacterium	d__Bacteria;p__Proteobacteria;c__Gammaproteobacteria;o__Enterobacterales;f__Enterobacteriaceae;g__Escherichia-Shigella	1	0.00
FAGH01000127.1.1295	Multiplex	d__Bacteria;p__Firmicutes;c__Clostridia;o__Peptostreptococcales-Tissierellales;f__Peptostreptococcaceae;g__Clostridioides;s__Clostridioides_difficile	d__Bacteria;p__Firmicutes;c__Clostridia;o__Peptostreptococcales-Tissierellales;f__Peptostreptococcaceae;g__Clostridioides	16	0.02
EU772591.1.1407	Multiplex	d__Bacteria;p__Firmicutes;c__Bacilli;o__Lactobacillales;f__Streptococcaceae;g__Streptococcus;s__uncultured_bacterium	d__Bacteria;p__Firmicutes;c__Bacilli;o__Lactobacillales;f__Streptococcaceae;g__Streptococcus	42	0.05

CP012266.244171.2457	Multiplex			2	0.00
CQBO01000060.27.1557	Multiplex	d__Bacteria;p__Proteobacteria;c__Gammaproteobacteria;o__Enterobacterales;f__Yersiniaceae;g__Yersinia;s__Yersinia_frederiksenii	d__Bacteria;p__Proteobacteria;c__Gammaproteobacteria;o__Enterobacterales;f__Yersiniaceae;g__Yersinia	6	0.01
GU808376.1.1496	Multiplex	d__Bacteria;p__Proteobacteria;c__Gammaproteobacteria;o__Enterobacterales;f__Erwiniaceae;g__Pantoea;s__uncultured_bacterium	d__Bacteria;p__Proteobacteria;c__Gammaproteobacteria;o__Enterobacterales;f__Erwiniaceae;g__Pantoea	10	0.01
GQ897892.1.1481	Multiplex	d__Bacteria;p__Firmicutes;c__Clostridia;o__Peptostreptococcales-Tissierellales;f__Peptostreptococcaceae;g__Romboutsia;s__uncultured_bacterium	d__Bacteria;p__Firmicutes;c__Clostridia;o__Peptostreptococcales-Tissierellales;f__Peptostreptococcaceae;g__Romboutsia	15	0.02
CP009846.3963135.396	Multiplex			28	0.03
AM277978.1.1525	Multiplex	d__Bacteria;p__Actinobacteriota;c__Actinobacteria;o__Bifidobacteriales;f__Bifidobacteriaceae;g__Bifidobacterium;s__uncultured_bacterium	d__Bacteria;p__Actinobacteriota;c__Actinobacteria;o__Bifidobacteriales;f__Bifidobacteriaceae;g__Bifidobacterium	9	0.01
EU460412.1.1431_001	Multiplex			91.7	0.11
CP020424.2041390.204	Multiplex			8	0.01
EU772683.1.1391	Multiplex	d__Bacteria;p__Firmicutes;c__Clostridia;o__Lachnospirales;f__Lachnospiraceae;g__Blautia;s__uncultured_bacterium	d__Bacteria;p__Firmicutes;c__Clostridia;o__Lachnospirales;f__Lachnospiraceae;g__Blautia	23	0.03
EU774926.1.1421	Multiplex	d__Bacteria;p__Firmicutes;c__Bacilli;o__Lactobacillales;f__Lactobacillaceae;g__Lactobacillus;s__uncultured_bacterium	d__Bacteria;p__Firmicutes;c__Bacilli;o__Lactobacillales;f__Lactobacillaceae;g__Lactobacillus	1	0.00
GQ155746.1.1359	Multiplex	d__Bacteria;p__Firmicutes;c__Bacilli;o__Lactobacillales;f__Enterococcaceae;g__Enterococcus;s__uncultured_bacterium	d__Bacteria;p__Firmicutes;c__Bacilli;o__Lactobacillales;f__Enterococcaceae;g__Enterococcus	3	0.00
LF649083.20.1480	Multiplex	d__Bacteria;p__Firmicutes;c__Bacilli;o__Lactobacillales;f__Lactobacillaceae;g__Lactobacillus;s__Lactobacillus_paraplantarum	d__Bacteria;p__Firmicutes;c__Bacilli;o__Lactobacillales;f__Lactobacillaceae;g__Lactobacillus	14	0.02
FN665654.138327.139831_006	Multiplex			19224	22.46

MK045817.1.1425	Multiplex	d__Bacteria;p__Actinobacteriota;c__Actinobacteria;o__Bifidobacteriales;f__Bifidobacteriaceae;g__Bifidobacterium;s__Bifidobacterium_longum	d__Bacteria;p__Actinobacteriota;c__Actinobacteria;o__Bifidobacteriales;f__Bifidobacteriaceae;g__Bifidobacterium	6	0.01
EU776680.1.1401_001	Multiplex			16.99	0.02
DQ814052.1.1485	Multiplex	d__Bacteria;p__Proteobacteria;c__Gammaproteobacteria;o__Enterobacterales;f__Yersiniaceae;g__Serratia;s__uncultured_bacterium	d__Bacteria;p__Proteobacteria;c__Gammaproteobacteria;o__Enterobacterales;f__Yersiniaceae;g__Serratia	14	0.02
DQ089673.1.1505	Multiplex	d__Bacteria;p__Proteobacteria;c__Gammaproteobacteria;o__Enterobacterales;f__Enterobacteriaceae;g__Enterobacter;s__Enterobacter_cloacae	d__Bacteria;p__Proteobacteria;c__Gammaproteobacteria;o__Enterobacterales;f__Enterobacteriaceae;g__Enterobacter	2	0.00
FJ976017.1.1357	Multiplex	d__Bacteria;p__Firmicutes;c__Bacilli;o__Lactobacillales;f__Lactobacillaceae;g__Lactobacillus;s__uncultured_Lactobacillaceae	d__Bacteria;p__Firmicutes;c__Bacilli;o__Lactobacillales;f__Lactobacillaceae;g__Lactobacillus	1	0.00
HQ804113.1.1452	Multiplex	d__Bacteria;p__Bacteroidota;c__Bacteroidia;o__Bacteroidales;f__Bacteroidaceae;g__Bacteroides;s__uncultured_organism	d__Bacteria;p__Bacteroidota;c__Bacteroidia;o__Bacteroidales;f__Bacteroidaceae;g__Bacteroides	12	0.01
HQ767681.1.1432	Multiplex	d__Bacteria;p__Bacteroidota;c__Bacteroidia;o__Bacteroidales;f__Bacteroidaceae;g__Bacteroides;s__uncultured_organism	d__Bacteria;p__Bacteroidota;c__Bacteroidia;o__Bacteroidales;f__Bacteroidaceae;g__Bacteroides	27	0.03
JF043143.1.1362	Multiplex	d__Bacteria;p__Proteobacteria;c__Gammaproteobacteria;o__Enterobacterales;f__Enterobacteriaceae;g__Klebsiella;s__uncultured_bacterium	d__Bacteria;p__Proteobacteria;c__Gammaproteobacteria;o__Enterobacterales;f__Enterobacteriaceae;g__Klebsiella	24	0.03
DQ795160.1.1400	Multiplex	d__Bacteria;p__Bacteroidota;c__Bacteroidia;o__Bacteroidales;f__Prevotellaceae;g__Prevotella;s__uncultured_bacterium	d__Bacteria;p__Bacteroidota;c__Bacteroidia;o__Bacteroidales;f__Prevotellaceae;g__Prevotella	2	0.00
EU767611.1.1349	Multiplex	d__Bacteria;p__Actinobacteriota;c__Actinobacteria;o__Bifidobacteriales;f__Bifidobacteriaceae;g__Bifidobacterium;s__uncultured_bacterium	d__Bacteria;p__Actinobacteriota;c__Actinobacteria;o__Bifidobacteriales;f__Bifidobacteriaceae;g__Bifidobacterium	5	0.01
EU467709.1.1380	Multiplex	d__Bacteria;p__Bacteroidota;c__Bacteroidia;o__Bacteroidales;f__Bacteroidaceae;g__Bacteroides;s__uncultured_bacterium	d__Bacteria;p__Bacteroidota;c__Bacteroidia;o__Bacteroidales;f__Bacteroidaceae;g__Bacteroides	9	0.01
JQ002525.1.1373	Multiplex	d__Bacteria;p__Actinobacteriota;c__Actinobacteria;o__Bifidobacteriales;f	d__Bacteria;p__Actinobacteriota;c__Actinobacteria;o__Bifidobacteriales;f	4	0.00

		__Bifidobacteriaceae;g__Bifidobacterium;s__Bifidobacterium_choerinum	__Bifidobacteriaceae;g__Bifidobacterium		
DQ799377.1.1384	Multiplex	d__Bacteria;p__Firmicutes;c__Clostridia;o__Oscillospirales;f__Ruminococcaceae;g__Ruminococcus;s__uncultured_bacterium	d__Bacteria;p__Firmicutes;c__Clostridia;o__Oscillospirales;f__Ruminococcaceae;g__Ruminococcus	1	0.00
JQ475464.1.1374	Multiplex	d__Bacteria;p__Fusobacteriota;c__Fusobacteriia;o__Fusobacteriales;f__Fusobacteriaceae;g__Fusobacterium;s__uncultured_bacterium	d__Bacteria;p__Fusobacteriota;c__Fusobacteriia;o__Fusobacteriales;f__Fusobacteriaceae;g__Fusobacterium	29	0.03
CP017954.2756793.275	Multiplex			1	0.00
GQ477833.1.1442	Multiplex	d__Bacteria;p__Proteobacteria;c__Alphaproteobacteria;o__Acetobacterales;f__Acetobacteraceae;g__Acetobacter;s__uncultured_bacterium	d__Bacteria;p__Proteobacteria;c__Alphaproteobacteria;o__Acetobacterales;f__Acetobacteraceae;g__Acetobacter	14	0.02
FPLN01008504.13.1528	Multiplex	d__Bacteria;p__Proteobacteria;c__Gammaproteobacteria;o__Pseudomonadales;f__Pseudomonadaceae;g__Pseudomonas;s__metagenome	d__Bacteria;p__Proteobacteria;c__Gammaproteobacteria;o__Pseudomonadales;f__Pseudomonadaceae;g__Pseudomonas	1	0.00
JQ468014.1.1391	Multiplex	d__Bacteria;p__Bacteroidota;c__Bacteroidia;o__Flavobacteriales;f__Flavobacteriaceae;g__Capnocytophaga;s__uncultured_bacterium	d__Bacteria;p__Bacteroidota;c__Bacteroidia;o__Flavobacteriales;f__Flavobacteriaceae;g__Capnocytophaga	3	0.00
HQ800683.1.1439	Multiplex	d__Bacteria;p__Bacteroidota;c__Bacteroidia;o__Bacteroidales;f__Bacteroidaceae;g__Bacteroides;s__uncultured_organism	d__Bacteria;p__Bacteroidota;c__Bacteroidia;o__Bacteroidales;f__Bacteroidaceae;g__Bacteroides	14	0.02
FJ975799.1.1361	Multiplex	d__Bacteria;p__Firmicutes;c__Bacilli;o__Lactobacillales;f__Enterococcaceae;g__Enterococcus;s__uncultured_Lactobacillales	d__Bacteria;p__Firmicutes;c__Bacilli;o__Lactobacillales;f__Enterococcaceae;g__Enterococcus	28	0.03
EF097527.1.1405_001	Multiplex			0.99	0.00
GQ448246.1.1389	Multiplex	d__Bacteria;p__Bacteroidota;c__Bacteroidia;o__Bacteroidales;f__Bacteroidaceae;g__Bacteroides;s__uncultured_bacterium	d__Bacteria;p__Bacteroidota;c__Bacteroidia;o__Bacteroidales;f__Bacteroidaceae;g__Bacteroides	11	0.01
D86186.1.1530	Multiplex	d__Bacteria;p__Actinobacteriota;c__Actinobacteria;o__Bifidobacteriales;f__Bifidobacteriaceae;g__Bifidobacterium;s__Bifidobacterium_choerinum	d__Bacteria;p__Actinobacteriota;c__Actinobacteria;o__Bifidobacteriales;f__Bifidobacteriaceae;g__Bifidobacterium	8	0.01

AB969632.1.1565	Multiplex	d__Bacteria;p__Firmicutes;c__Bacilli;o__Lactobacillales;f__Lactobacillaceae;g__Lactobacillus;s__uncultured_bacterium	d__Bacteria;p__Firmicutes;c__Bacilli;o__Lactobacillales;f__Lactobacillaceae;g__Lactobacillus	1	0.00
DQ798156.1.1398	Multiplex	d__Bacteria;p__Bacteroidota;c__Bacteroidia;o__Bacteroidales;f__Bacteroidaceae;g__Bacteroides;s__uncultured_bacterium	d__Bacteria;p__Bacteroidota;c__Bacteroidia;o__Bacteroidales;f__Bacteroidaceae;g__Bacteroides	2	0.00
EU458418.1.1398	Multiplex	d__Bacteria;p__Firmicutes;c__Clostridia;o__Lachnospirales;f__Lachnospiraceae;g__[Ruminococcus]_torques_group;s__uncultured_bacterium	d__Bacteria;p__Firmicutes;c__Clostridia;o__Lachnospirales;f__Lachnospiraceae;g__[Ruminococcus]_torques_group	38	0.04
FN667917.1.1426	Multiplex	d__Bacteria;p__Firmicutes;c__Bacilli;o__Lactobacillales;f__Lactobacillaceae;g__Lactobacillus;s__Lactobacillus_sp.	d__Bacteria;p__Firmicutes;c__Bacilli;o__Lactobacillales;f__Lactobacillaceae;g__Lactobacillus	2	0.00
JQ477027.1.1419	Multiplex	d__Bacteria;p__Actinobacteriota;c__Actinobacteria;o__Actinomycetales;f__Actinomycetaceae;g__Actinomyces;s__uncultured_bacterium	d__Bacteria;p__Actinobacteriota;c__Actinobacteria;o__Actinomycetales;f__Actinomycetaceae;g__Actinomyces	11	0.01
KF842135.1.1408	Multiplex	d__Bacteria;p__Bacteroidota;c__Bacteroidia;o__Bacteroidales;f__Bacteroidaceae;g__Bacteroides;s__uncultured_bacterium	d__Bacteria;p__Bacteroidota;c__Bacteroidia;o__Bacteroidales;f__Bacteroidaceae;g__Bacteroides	34	0.04
EU778419.1.1388_001	Multiplex			92.61	0.11
EU456966.1.1435_002	Multiplex			1.16	0.00
EU762218.1.1357	Multiplex	d__Bacteria;p__Bacteroidota;c__Bacteroidia;o__Bacteroidales;f__Bacteroidaceae;g__Bacteroides;s__uncultured_bacterium	d__Bacteria;p__Bacteroidota;c__Bacteroidia;o__Bacteroidales;f__Bacteroidaceae;g__Bacteroides	34	0.04
HQ762784.1.1447_001	Multiplex			463.2	0.54
EU560876.1.1492_014	Multiplex			11.9	0.01
AM286415.3550757.355	Multiplex			1	0.00
EF097373.1.1429	Multiplex	d__Bacteria;p__Firmicutes;c__Bacilli;o__Lactobacillales;f__Lactobacillaceae;g__Lactobacillus;s__uncultured_bacterium	d__Bacteria;p__Firmicutes;c__Bacilli;o__Lactobacillales;f__Lactobacillaceae;g__Lactobacillus	7	0.01

CP010888.648648.6501	Multiplex			28	0.03
EU511760.1.1389_004	Multiplex			1.99	0.00
DQ794217.1.1397	Multiplex	d__Bacteria;p__Bacteroidota;c__Bacteroidia;o__Bacteroidales;f__Bacteroidaceae;g__Bacteroides;s__uncultured_bacterium	d__Bacteria;p__Bacteroidota;c__Bacteroidia;o__Bacteroidales;f__Bacteroidaceae;g__Bacteroides	7	0.01
EU777738.1.1370	Multiplex	d__Bacteria;p__Firmicutes;c__Clostridia;o__Clostridiales;f__Clostridiaceae;g__Sarcina;s__uncultured_bacterium	d__Bacteria;p__Firmicutes;c__Clostridia;o__Clostridiales;f__Clostridiaceae;g__Sarcina	3	0.00
EU287343.1.1529	Multiplex	d__Bacteria;p__Firmicutes;c__Bacilli;o__Lactobacillales;f__Lactobacillaceae;g__Lactobacillus;s__uncultured_bacterium	d__Bacteria;p__Firmicutes;c__Bacilli;o__Lactobacillales;f__Lactobacillaceae;g__Lactobacillus	12	0.01
EU775617.1.1401	Multiplex	d__Bacteria;p__Proteobacteria;c__Gammaproteobacteria;o__Enterobacterales;f__Enterobacteriaceae;g__Escherichia-Shigella;s__uncultured_bacterium	d__Bacteria;p__Proteobacteria;c__Gammaproteobacteria;o__Enterobacterales;f__Enterobacteriaceae;g__Escherichia-Shigella	37	0.04
KF817757.1.1414	Multiplex	d__Bacteria;p__Proteobacteria;c__Gammaproteobacteria;o__Enterobacterales;f__Yersiniaceae;g__Rahnella1;s__Rahnella_aquatilis	d__Bacteria;p__Proteobacteria;c__Gammaproteobacteria;o__Enterobacterales;f__Yersiniaceae;g__Rahnella1	5	0.01
EU457166.1.1396	Multiplex	d__Bacteria;p__Bacteroidota;c__Bacteroidia;o__Bacteroidales;f__Bacteroidaceae;g__Bacteroides;s__uncultured_bacterium	d__Bacteria;p__Bacteroidota;c__Bacteroidia;o__Bacteroidales;f__Bacteroidaceae;g__Bacteroides	2	0.00
EU458418.1.1398_015	Multiplex			119	0.14
KT424977.1.1468	Multiplex	d__Bacteria;p__Proteobacteria;c__Gammaproteobacteria;o__Alteromonadales;f__Shewanellaceae;g__Shewanella;s__Shewanella_sp.	d__Bacteria;p__Proteobacteria;c__Gammaproteobacteria;o__Alteromonadales;f__Shewanellaceae;g__Shewanella	46	0.05
CP002246.1189358.119	Multiplex			2	0.00
BAAX01003885.346.185	Multiplex			24	0.03
EU778512.1.1403_001	Multiplex			42.13	0.05
CP019858.124243.1257	Multiplex			16	0.02

EU510219.1.1285_014	Multiplex			1.16	0.00
DQ824048.1.1400_001	Multiplex			0.18	0.00
AL935263.2944928.294	Multiplex			2	0.00
KM819108.1.1529	Multiplex	d__Bacteria;p__Firmicutes;c__Bacilli;o__Lactobacillales;f__Lactobacillaceae;g__Lactobacillus;s__uncultured_Bacillus	d__Bacteria;p__Firmicutes;c__Bacilli;o__Lactobacillales;f__Lactobacillaceae;g__Lactobacillus	1	0.00
EU775617.1.1401_001	Multiplex			1896.28	2.22
HQ818432.1.1426	Multiplex	d__Bacteria;p__Bacteroidota;c__Bacteroidia;o__Bacteroidales;f__Bacteroidaceae;g__Bacteroides;s__uncultured_organism	d__Bacteria;p__Bacteroidota;c__Bacteroidia;o__Bacteroidales;f__Bacteroidaceae;g__Bacteroides	11	0.01
HQ774712.1.1452_001	Multiplex			0.3	0.00
CP019857.10819.12310	Multiplex	d__Bacteria;p__Firmicutes;c__Clostridia;o__Peptostreptococcales-Tissierellales;f__Peptostreptococcaceae;g__Clostridioides;s__Clostridioides_difficile	d__Bacteria;p__Firmicutes;c__Clostridia;o__Peptostreptococcales-Tissierellales;f__Peptostreptococcaceae;g__Clostridioides	13	0.02
CQBX01000005.209397.	Multiplex			11	0.01
EU451957.1.1393_001	Multiplex			3	0.00
KM819135.1.1519	Multiplex	d__Bacteria;p__Firmicutes;c__Bacilli;o__Lactobacillales;f__Lactobacillaceae;g__Lactobacillus;s__uncultured_Bacillus	d__Bacteria;p__Firmicutes;c__Bacilli;o__Lactobacillales;f__Lactobacillaceae;g__Lactobacillus	2	0.00
CP022544.1495640.149	Multiplex			1	0.00
CP027190.484364.4859	Multiplex			16	0.02
FPLN01003709.10.1525	Multiplex	d__Bacteria;p__Proteobacteria;c__Gammaproteobacteria;o__Pseudomonadales;f__Pseudomonadaceae;g__Pseudomonas;s__metagenome	d__Bacteria;p__Proteobacteria;c__Gammaproteobacteria;o__Pseudomonadales;f__Pseudomonadaceae;g__Pseudomonas	9	0.01
DQ800097.1.1403	Multiplex	d__Bacteria;p__Firmicutes;c__Clostridia;o__Lachnospirales;f__Lachnospiraceae;g__Lachnospiraceae_UCG-004;s__uncultured_bacterium	d__Bacteria;p__Firmicutes;c__Clostridia;o__Lachnospirales;f__Lachnospiraceae;g__Lachnospiraceae_UCG-004	11	0.01

MH548358.1.1508					Multiplex			d__Bacteria;p__Firmicutes;c__Bacilli;o__Lactobacillales;f__Lactobacillaceae;g__Lactobacillus;s__Lactobacillus_plantarum	d__Bacteria;p__Firmicutes;c__Bacilli;o__Lactobacillales;f__Lactobacillaceae;g__Lactobacillus	1	0.00
JQ809309.1.1538					Multiplex			d__Bacteria;p__Firmicutes;c__Bacilli;o__Lactobacillales;f__Lactobacillaceae;g__Lactobacillus;s__uncultured_Lactobacillaceae	d__Bacteria;p__Firmicutes;c__Bacilli;o__Lactobacillales;f__Lactobacillaceae;g__Lactobacillus	1	0.00
EF406803.1.1509_078					Multiplex					0.02	0.00
DQ800202.1.1374					Multiplex			d__Bacteria;p__Firmicutes;c__Clostridia;o__Oscillospirales;f__Ruminococcaceae;g__Ruminococcus;s__uncultured_bacterium	d__Bacteria;p__Firmicutes;c__Clostridia;o__Oscillospirales;f__Ruminococcaceae;g__Ruminococcus	2	0.00
JQ861287.1.1510_001	1eb3021faaec49c2_1	97.543	4584412	4582910	Multiplex	Escherichia_coli_ATCC_700926	True			0.42	0.00
JN208201.1.1488	1eb3021faaec49c2_1	97.383	3660897	3659410	Multiplex	Escherichia_coli_ATCC_700926	True	d__Bacteria;p__Proteobacteria;c__Gammaproteobacteria;o__Enterobacterales;f__Enterobacteriaceae;g__Enterobacter;s__Enterobacter_sp.	d__Bacteria;p__Proteobacteria;c__Gammaproteobacteria;o__Enterobacterales;f__Enterobacteriaceae;g__Enterobacter	29	0.03
CP031558.1500822.1502336_001	4762fdd895094939_1	98.328	96370	97853	Multiplex	Helicobacter_pylori_ATCC_700392	True			1234	1.44
DQ325862.1.1349	c45a1c57adf4460e_2	98.298	23119	24468	Multiplex	Bifidobacterium_adolescentis_ATCC_15703	True	d__Bacteria;p__Actinobacteriota;c__Actinobacteria;o__Bifidobacteriales;f__Bifidobacteriaceae;g__Bifidobacterium;s__uncultured_bacterium	d__Bacteria;p__Actinobacteriota;c__Actinobacteria;o__Bifidobacteriales;f__Bifidobacteriaceae;g__Bifidobacterium	3	0.00
EU451160.1.1434_001	5964adb8d0df4fde_1	97.629	477450	478879	Multiplex	Lactobacillus_gasseri_ATCC_33323	True			2.15	0.00
HQ807338.1.1448	1eb3021faaec49c2_1	98.758	3660866	3659419	Multiplex	Escherichia_coli_ATCC_700926	True	d__Bacteria;p__Proteobacteria;c__Gammaproteobacteria;o__Enterobacterales;f__Enterobacteriaceae;g__Escherichia-Shigella;s__uncultured_organism	d__Bacteria;p__Proteobacteria;c__Gammaproteobacteria;o__Enterobacterales;f__Enterobacteriaceae;g__Escherichia-Shigella	4	0.00
MH105767.1.1436	27b35dd1651e48dd_1	98.468	213417	214852	Multiplex	Enterococcus_faecalis_ATCC_47077	True	d__Bacteria;p__Firmicutes;c__Bacilli;o__Lactobacillales;f__Enterococcaceae;g__Enterococcus;s__Enterococcus_faecalis	d__Bacteria;p__Firmicutes;c__Bacilli;o__Lactobacillales;f__Enterococcaceae;g__Enterococcus	25	0.03

CP029907.3714363.371	1eb3021faaec49c2_1	97.508	1152580	1154102	Multiplex	Escherichia_coli_ATCC_700926	True			8	0.01
CP024019.97500.99009	4762fdd895094939_1	99.73	96370	97853	Multiplex	Helicobacter_pylori_ATCC_700392	True	d__Bacteria;p__Campilobacterota;c__Campylobacteria;o__Campylobacterales;f__Helicobacteraceae;g__Helicobacter;s__Helicobacter_pylori	d__Bacteria;p__Campilobacterota;c__Campylobacteria;o__Campylobacterales;f__Helicobacteraceae;g__Helicobacter	4	0.00
GQ155927.1.1371	27b35dd1651e48dd_1	98.939	213471	214789	Multiplex	Enterococcus_faecalis_ATCC_47077	True	d__Bacteria;p__Firmicutes;c__Bacilli;o__Lactobacillales;f__Enterococcaceae;g__Enterococcus;s__uncultured_bacterium	d__Bacteria;p__Firmicutes;c__Bacilli;o__Lactobacillales;f__Enterococcaceae;g__Enterococcus	5	0.01
HQ762728.1.1437	d889f9dd971b473d_1	97.985	2587752	2589184	Multiplex	Phocaeicola_vulgatus_ATCC_8482	True	d__Bacteria;p__Bacteroidota;c__Bacteroidia;o__Bacteroidales;f__Bacteroidaceae;g__Bacteroides;s__uncultured_organism	d__Bacteria;p__Bacteroidota;c__Bacteroidia;o__Bacteroidales;f__Bacteroidaceae;g__Bacteroides	4	0.00
DQ905255.1.1454	d889f9dd971b473d_1	98.418	2095469	2096922	Multiplex	Phocaeicola_vulgatus_ATCC_8482	True	d__Bacteria;p__Bacteroidota;c__Bacteroidia;o__Bacteroidales;f__Bacteroidaceae;g__Bacteroides;s__uncultured_bacterium	d__Bacteria;p__Bacteroidota;c__Bacteroidia;o__Bacteroidales;f__Bacteroidaceae;g__Bacteroides	6	0.01
HQ793115.1.1453	d889f9dd971b473d_1	98.486	348208	346822	Multiplex	Phocaeicola_vulgatus_ATCC_8482	True	d__Bacteria;p__Bacteroidota;c__Bacteroidia;o__Bacteroidales;f__Bacteroidaceae;g__Bacteroides;s__uncultured_organism	d__Bacteria;p__Bacteroidota;c__Bacteroidia;o__Bacteroidales;f__Bacteroidaceae;g__Bacteroides	4	0.00
MXOH01000001.553880.555401_001	1eb3021faaec49c2_1	97.639	1152580	1154101	Multiplex	Escherichia_coli_ATCC_700926	True			0.47	0.00
AP011943.1149268.115	4762fdd895094939_1	99.461	96370	97853	Multiplex	Helicobacter_pylori_ATCC_700392	True			15	0.02
AP009256.1988380.198	c45a1c57adf4460e_2	100	23099	24608	Multiplex	Bifidobacterium_adolescentis_ATCC_15703	True			19	0.02
CP002246.4158244.4159734_001	1eb3021faaec49c2_1	97.455	3660889	3659402	Multiplex	Escherichia_coli_ATCC_700926	True			0.3	0.00
HQ774224.1.1417	d889f9dd971b473d_1	99.012	2095476	2096890	Multiplex	Phocaeicola_vulgatu	True	d__Bacteria;p__Bacteroidota;c__Bacteroidia;o__Bacteroidales;f__Bacteroi	d__Bacteria;p__Bacteroidota;c__Bacteroidia;o__Bacteroidales;f__Bacteroidaceae;g__Bacteroides	3	0.00

						s_ATCC_8482		daceae;g__Bacteroides;s__uncultured_organism			
CP009561.1275010.127	1eb3021faaec49c2_1	97.507	1152580	1154101	Multiplex	Escherichia_coli_ATCC_700926	True			2	0.00
CP030233.4191849.419	1eb3021faaec49c2_1	97.244	1152580	1154101	Multiplex	Escherichia_coli_ATCC_700926	True			3	0.00
JQ446508.1.1373	27b35dd1651e48dd_1	98.621	213452	214827	Multiplex	Enterococcus_faecalis_ATCC_47077	True	d__Bacteria;p__Firmicutes;c__Bacilli;o__Lactobacillales;f__Enterococcaceae;g__Enterococcus;s__Enterococcus_faecalis	d__Bacteria;p__Firmicutes;c__Bacilli;o__Lactobacillales;f__Enterococcaceae;g__Enterococcus	2	0.00
CP029992.3310618.331	1eb3021faaec49c2_1	97.636	1152580	1154101	Multiplex	Escherichia_coli_ATCC_700926	True			1	0.00
CP011975.2004273.200					Multiplex					2	0.00
KM506787.1.1448					Multiplex			d__Bacteria;p__Firmicutes;c__Bacilli;o__Lactobacillales;f__Lactobacillaceae;g__Lactobacillus;s__Lactobacillus_plantarum	d__Bacteria;p__Firmicutes;c__Bacilli;o__Lactobacillales;f__Lactobacillaceae;g__Lactobacillus	2	0.00
CP009712.4129838.413					Multiplex					7	0.01
EU777753.1.1312					Multiplex			d__Bacteria;p__Proteobacteria;c__Gammaproteobacteria;o__Enterobacterales;f__Enterobacteriaceae;g__Escherichia-Shigella;s__uncultured_bacterium	d__Bacteria;p__Proteobacteria;c__Gammaproteobacteria;o__Enterobacterales;f__Enterobacteriaceae;g__Escherichia-Shigella	5	0.01
EU767274.1.1360					Multiplex			d__Bacteria;p__Actinobacteriota;c__Actinobacteria;o__Bifidobacteriales;f__Bifidobacteriaceae;g__Bifidobacterium;s__uncultured_bacterium	d__Bacteria;p__Actinobacteriota;c__Actinobacteria;o__Bifidobacteriales;f__Bifidobacteriaceae;g__Bifidobacterium	6	0.01
AJKY01000083.3720.52					Multiplex					21	0.02
FN665654.138327.1398					Multiplex					12	0.01
HQ761701.1.1434					Multiplex			d__Bacteria;p__Bacteroidota;c__Bacteroidia;o__Bacteroidales;f__Bacteroidaceae;g__Bacteroides;s__uncultured_organism	d__Bacteria;p__Bacteroidota;c__Bacteroidia;o__Bacteroidales;f__Bacteroidaceae;g__Bacteroides	3	0.00

EU454583.1.1411_019					Multiplex					2.99	0.00
CP028526.334281.3357					Multiplex					35	0.04
CQEC01000020.91536.9					Multiplex					28	0.03
AY634580.1.1308					Multiplex			d__Bacteria;p__Campilobacterota;c__Campylobacteria;o__Campylobacterales;f__Helicobacteraceae;g__Helicobacter;s__uncultured_Helicobacter	d__Bacteria;p__Campilobacterota;c__Campylobacteria;o__Campylobacterales;f__Helicobacteraceae;g__Helicobacter	28	0.03
CP009364.3139876.314					Multiplex					17	0.02
DQ797217.1.1403					Multiplex			d__Bacteria;p__Bacteroidota;c__Bacteroidia;o__Bacteroidales;f__Bacteroidaceae;g__Bacteroides;s__uncultured_bacterium	d__Bacteria;p__Bacteroidota;c__Bacteroidia;o__Bacteroidales;f__Bacteroidaceae;g__Bacteroides	9	0.01
CP012323.10819.12309					Multiplex			d__Bacteria;p__Firmicutes;c__Clostridia;o__Peptostreptococcales-Tissierellales;f__Peptostreptococcaceae;g__Clostridioides;s__Clostridioides_difficile	d__Bacteria;p__Firmicutes;c__Clostridia;o__Peptostreptococcales-Tissierellales;f__Peptostreptococcaceae;g__Clostridioides	7	0.01
HQ810958.1.1447_001	d889f9dd971b473d_1	98.135	348197	346759	Multiplex	Phocaeicola_vulgatus_ATCC_8482	True			0.32	0.00
HQ793333.1.1443	1eb3021faaec49c2_1	99.422	3660881	3659498	Multiplex	Escherichia_coli_ATCC_700926	True	d__Bacteria;p__Proteobacteria;c__Gammaproteobacteria;o__Enterobacterales;f__Enterobacteriaceae;g__Escherichia-Shigella;s__uncultured_organism	d__Bacteria;p__Proteobacteria;c__Gammaproteobacteria;o__Enterobacterales;f__Enterobacteriaceae;g__Escherichia-Shigella	9	0.01
JQ187021.1.1359	c45a1c57adf4460e_1	97.277	1850147	1848801	Multiplex	Bifidobacterium_adolescentis_ATCC_15703	True	d__Bacteria;p__Actinobacteriota;c__Actinobacteria;o__Bifidobacteriales;f__Bifidobacteriaceae;g__Bifidobacterium;s__uncultured_bacterium	d__Bacteria;p__Actinobacteriota;c__Actinobacteria;o__Bifidobacteriales;f__Bifidobacteriaceae;g__Bifidobacterium	47	0.05
AF506794.1.1466	4762fdd895094939_1	97.614	96370	97835	Multiplex	Helicobacter_pylori_ATCC_700392	True	d__Bacteria;p__Campilobacterota;c__Campylobacteria;o__Campylobacterales;f__Helicobacteraceae;g__Helicobacter;s__Helicobacter_heilmannii	d__Bacteria;p__Campilobacterota;c__Campylobacteria;o__Campylobacterales;f__Helicobacteraceae;g__Helicobacter	1	0.00
EU655795.1.1320_001					Multiplex					1	0.00
HQ741049.1.1434_001	d889f9dd971b473d_1	98.953	4744593	4743163	Multiplex	Phocaeicola_vulgatu	True			6718.65	7.85

						s_ATCC_8482					
HQ802459.1.1485_001	27b35dd1651e48dd_1	98.991	213399	214884	Multiplex	Enterococcus_faecalis_ATCC_47077	True			0.18	0.00
KF843118.1.1413_009	1eb3021faaec49c2_1	97.013	3660907	3659509	Multiplex	Escherichia_coli_ATCC_700926	True			2.79	0.00
EU136687.1.1985	d889f9dd971b473d_1	100	2095444	2096948	Multiplex	Phocaeicola_vulgatus_ATCC_8482	True	d__Bacteria;p__Bacteroidota;c__Bacteroidia;o__Bacteroidales;f__Bacteroidaceae;g__Bacteroides;s__Bacteroides_vulgatus	d__Bacteria;p__Bacteroidota;c__Bacteroidia;o__Bacteroidales;f__Bacteroidaceae;g__Bacteroides	2	0.00
HQ771701.1.1438_001	d889f9dd971b473d_1	98.956	348191	346759	Multiplex	Phocaeicola_vulgatus_ATCC_8482	True			0.3	0.00
FPLO01005702.1.1404	c45a1c57adf4460e_2	99.004	23204	24608	Multiplex	Bifidobacterium_adolescentis_ATCC_15703	True	d__Bacteria;p__Actinobacteriota;c__Actinobacteria;o__Bifidobacteriales;f__Bifidobacteriaceae;g__Bifidobacterium;s__metagenome	d__Bacteria;p__Actinobacteriota;c__Actinobacteria;o__Bifidobacteriales;f__Bifidobacteriaceae;g__Bifidobacterium	24	0.03
CP029595.3631004.363	1eb3021faaec49c2_1	97.244	1152580	1154101	Multiplex	Escherichia_coli_ATCC_700926	True			5	0.01
CP022712.1786281.1787846_001	27b35dd1651e48dd_1	99.351	213371	214911	Multiplex	Enterococcus_faecalis_ATCC_47077	True			2978.78	3.48
CP030236.2819838.2821391_004	1eb3021faaec49c2_1	97.967	1152580	1154101	Multiplex	Escherichia_coli_ATCC_700926	True			0.39	0.00
HQ810947.1.1443	d889f9dd971b473d_1	98.406	348195	346760	Multiplex	Phocaeicola_vulgatus_ATCC_8482	True	d__Bacteria;p__Bacteroidota;c__Bacteroidia;o__Bacteroidales;f__Bacteroidaceae;g__Bacteroides;s__uncultured_organism	d__Bacteria;p__Bacteroidota;c__Bacteroidia;o__Bacteroidales;f__Bacteroidaceae;g__Bacteroides	27	0.03
CP030838.463295.464833_001	1eb3021faaec49c2_1	97.246	454975	456496	Multiplex	Escherichia_coli_ATCC_700926	True			107.97	0.13

CP012268.3124033.3125588_001	1eb3021faaec49c2_1	97.375	3660909	3659388	Multiplex	Escherichia_coli_ATCC_700926	True			2929.26	3.42
EU723852.1.1527_001	27b35dd1651e48dd_1	99.147	213374	214889	Multiplex	Enterococcus_faecalis_ATCC_47077	True			0.18	0.00
ABAK02000001.2157381	1eb3021faaec49c2_1	97.705	3660909	3659388	Multiplex	Escherichia_coli_ATCC_700926	True			2	0.00
JN981928.1.1516_004	1eb3021faaec49c2_1	98.809	3660909	3659404	Multiplex	Escherichia_coli_ATCC_700926	True			0.2	0.00
D86197.1.1437_001	c45a1c57adf4460e_2	99.087	23185	24608	Multiplex	Bifidobacterium_adolescentis_ATCC_15703	True			6048.81	7.07
CP026197.417048.4185	1eb3021faaec49c2_1	97.113	1152580	1154101	Multiplex	Escherichia_coli_ATCC_700926	True			2	0.00
AJ301831.1.1554	27b35dd1651e48dd_1	99.676	213371	214911	Multiplex	Enterococcus_faecalis_ATCC_47077	True	d__Bacteria;p__Firmicutes;c__Bacilli;o__Lactobacillales;f__Enterococcaceae;g__Enterococcus;s__Enterococcus_faecalis	d__Bacteria;p__Firmicutes;c__Bacilli;o__Lactobacillales;f__Enterococcaceae;g__Enterococcus	14	0.02
CP023005.2008138.200	c45a1c57adf4460e_1	99.934	1850167	1848661	Multiplex	Bifidobacterium_adolescentis_ATCC_15703	True			44	0.05
AP011945.1176263.117	4762fdd895094939_1	99.663	96370	97853	Multiplex	Helicobacter_pylori_ATCC_700392	True			4	0.00
CXRJ01000078.4200.57	27b35dd1651e48dd_1	97.542	213371	214911	Multiplex	Enterococcus_faecalis_ATCC_47077	True			7	0.01

HQ778631.1.1433	d889f9dd971b473d_1	97.491	2587764	2589193	Multiplex	Phocaeicola_vulgatus_ATCC_8482	True	d__Bacteria;p__Bacteroidota;c__Bacteroidia;o__Bacteroidales;f__Bacteroidaceae;g__Bacteroides;s__uncultured_organism	d__Bacteria;p__Bacteroidota;c__Bacteroidia;o__Bacteroidales;f__Bacteroidaceae;g__Bacteroides	6	0.01
HQ814803.1.1453	d889f9dd971b473d_1	98.986	4431936	4430557	Multiplex	Phocaeicola_vulgatus_ATCC_8482	True	d__Bacteria;p__Bacteroidota;c__Bacteroidia;o__Bacteroidales;f__Bacteroidaceae;g__Bacteroides;s__uncultured_organism	d__Bacteria;p__Bacteroidota;c__Bacteroidia;o__Bacteroidales;f__Bacteroidaceae;g__Bacteroides	2	0.00
CP026807.3341963.334	1eb3021faaec49c2_1	99.671	1152580	1154101	Multiplex	Escherichia_coli_ATCC_700926	True			2	0.00
AP012600.1432722.143	4762fdd895094939_1	99.73	96370	97853	Multiplex	Helicobacter_pylori_ATCC_700392	True			4	0.00
HQ739773.1.1442	1eb3021faaec49c2_1	99.141	1152596	1153991	Multiplex	Escherichia_coli_ATCC_700926	True	d__Bacteria;p__Proteobacteria;c__Gammaproteobacteria;o__Enterobacterales;f__Enterobacteriaceae;g__Escherichia-Shigella;s__uncultured_bacterium	d__Bacteria;p__Proteobacteria;c__Gammaproteobacteria;o__Enterobacterales;f__Enterobacteriaceae;g__Escherichia-Shigella	2	0.00
LT558811.1.1486	c45a1c57adf4460e_1	99.048	1850154	1848685	Multiplex	Bifidobacterium_adolescentis_ATCC_15703	True	d__Bacteria;p__Actinobacteriota;c__Actinobacteria;o__Bifidobacteriales;f__Bifidobacteriaceae;g__Bifidobacterium;s__Bifidobacterium_adolescentis	d__Bacteria;p__Actinobacteriota;c__Actinobacteria;o__Bifidobacteriales;f__Bifidobacteriaceae;g__Bifidobacterium	9	0.01
CP015574.3854679.385	1eb3021faaec49c2_1	97.31	1152580	1154101	Multiplex	Escherichia_coli_ATCC_700926	True			3	0.00
CP022712.1786281.178	27b35dd1651e48dd_1	99.351	213371	214911	Multiplex	Enterococcus_faecalis_ATCC_47077	True			33	0.04
AY634599.1.1307	4762fdd895094939_1	99.375	96471	97750	Multiplex	Helicobacter_pylori_ATCC_700392	True	d__Bacteria;p__Campilobacterota;c__Campylobacteria;o__Campylobacterales;f__Helicobacteraceae;g__Helicobacter;s__uncultured_Helicobacter	d__Bacteria;p__Campilobacterota;c__Campylobacteria;o__Campylobacterales;f__Helicobacteraceae;g__Helicobacter	1	0.00
CP030235.2050034.205	1eb3021faaec49c2_1	97.31	1152580	1154101	Multiplex	Escherichia_coli_ATCC_700926	True			3	0.00

HQ815539.1.1438	d889f9dd971b473d_1	98.329	2587742	2589169	Multiplex	Phocaeicola_vulgatus_ATCC_8482	True	d__Bacteria;p__Bacteroidota;c__Bacteroidia;o__Bacteroidales;f__Bacteroidaceae;g__Bacteroides;s__uncultured_organism	d__Bacteria;p__Bacteroidota;c__Bacteroidia;o__Bacteroidales;f__Bacteroidaceae;g__Bacteroides	5	0.01
CP022139.4711002.471	1eb3021faaec49c2_1	97.244	1152580	1154101	Multiplex	Escherichia_coli_ATCC_700926	True			7	0.01
CP010437.2145122.214	c45a1c57adf4460e_2	98.844	23099	24566	Multiplex	Bifidobacterium_adolescentis_ATCC_15703	True			3	0.00
EU456992.1.1435_002	5964adb8d0df4fde_1	98.259	477450	478879	Multiplex	Lactobacillus_gasseri_ATCC_33323	True			0.02	0.00
CP026325.1211466.121	4762fdd895094939_1	100	96370	97853	Multiplex	Helicobacter_pylori_ATCC_700392	True			3	0.00
EU764185.1.1352	c45a1c57adf4460e_1	98.816	1850147	1848802	Multiplex	Bifidobacterium_adolescentis_ATCC_15703	True	d__Bacteria;p__Actinobacteriota;c__Actinobacteria;o__Bifidobacteriales;f__Bifidobacteriaceae;g__Bifidobacterium;s__uncultured_bacterium	d__Bacteria;p__Actinobacteriota;c__Actinobacteria;o__Bifidobacteriales;f__Bifidobacteriaceae;g__Bifidobacterium	37	0.04
FJ611793.1.1416	c45a1c57adf4460e_1	98.804	1850147	1848729	Multiplex	Bifidobacterium_adolescentis_ATCC_15703	True	d__Bacteria;p__Actinobacteriota;c__Actinobacteria;o__Bifidobacteriales;f__Bifidobacteriaceae;g__Bifidobacterium;s__Bifidobacterium_adolescentis	d__Bacteria;p__Actinobacteriota;c__Actinobacteria;o__Bifidobacteriales;f__Bifidobacteriaceae;g__Bifidobacterium	9	0.01
GQ155993.1.1370	27b35dd1651e48dd_1	97.801	213471	214789	Multiplex	Enterococcus_faecalis_ATCC_47077	True	d__Bacteria;p__Firmicutes;c__Bacilli;o__Lactobacillales;f__Enterococcaceae;g__Enterococcus;s__uncultured_bacterium	d__Bacteria;p__Firmicutes;c__Bacilli;o__Lactobacillales;f__Enterococcaceae;g__Enterococcus	5	0.01
AY850534.1.1248					Multiplex			d__Bacteria;p__Actinobacteriota;c__Actinobacteria;o__Bifidobacteriales;f__Bifidobacteriaceae;g__Bifidobacterium;s__uncultured_bacterium	d__Bacteria;p__Actinobacteriota;c__Actinobacteria;o__Bifidobacteriales;f__Bifidobacteriaceae;g__Bifidobacterium	3	0.00
HQ765079.1.1455					Multiplex			d__Bacteria;p__Bacteroidota;c__Bacteroidia;o__Bacteroidales;f__Bacteroidaceae;g__Bacteroides;s__uncultured_organism	d__Bacteria;p__Bacteroidota;c__Bacteroidia;o__Bacteroidales;f__Bacteroidaceae;g__Bacteroides	4	0.00

FPLN01001016.2.1422	Multiplex	d__Bacteria;p__Proteobacteria;c__Gammaproteobacteria;o__Pseudomonadales;f__Pseudomonadaceae;g__Pseudomonas;s__metagenome	d__Bacteria;p__Proteobacteria;c__Gammaproteobacteria;o__Pseudomonadales;f__Pseudomonadaceae;g__Pseudomonas	9	0.01
AJ133496.1.1479	Multiplex	d__Bacteria;p__Fusobacteriota;c__Fusobacteriia;o__Fusobacteriales;f__Fusobacteriaceae;g__Fusobacterium;s__Fusobacterium_nucleatum	d__Bacteria;p__Fusobacteriota;c__Fusobacteriia;o__Fusobacteriales;f__Fusobacteriaceae;g__Fusobacterium	67	0.08
KC011147.1.1494_005	Multiplex			85.34	0.10
EU775002.1.1308	Multiplex	d__Bacteria;p__Firmicutes;c__Clostridia;o__Oscillospirales;f__UCG-011;g__UCG-011;s__uncultured_bacterium	d__Bacteria;p__Firmicutes;c__Clostridia;o__Oscillospirales;f__UCG-011;g__UCG-011	4	0.00
EU463378.1.1375	Multiplex	d__Bacteria;p__Firmicutes;c__Clostridia;o__Clostridiales;f__Clostridiaceae;g__Clostridium_sensu_stricto_1;s__uncultured_bacterium	d__Bacteria;p__Firmicutes;c__Clostridia;o__Clostridiales;f__Clostridiaceae;g__Clostridium_sensu_stricto_1	4	0.00
DQ345464.1.1523	Multiplex	d__Bacteria;p__Firmicutes;c__Bacilli;o__Lactobacillales;f__Enterococcaceae;g__Tetragenococcus;s__uncultured_compost	d__Bacteria;p__Firmicutes;c__Bacilli;o__Lactobacillales;f__Enterococcaceae;g__Tetragenococcus	6	0.01
AJ621551.1.1466	Multiplex	d__Bacteria;p__Firmicutes;c__Bacilli;o__Lactobacillales;f__Lactobacillaceae;g__Lactobacillus;s__Lactobacillus_graminis	d__Bacteria;p__Firmicutes;c__Bacilli;o__Lactobacillales;f__Lactobacillaceae;g__Lactobacillus	5	0.01
EU776535.1.1401	Multiplex	d__Bacteria;p__Proteobacteria;c__Gammaproteobacteria;o__Enterobacterales;f__Enterobacteriaceae;g__Escherichia-Shigella;s__uncultured_bacterium	d__Bacteria;p__Proteobacteria;c__Gammaproteobacteria;o__Enterobacterales;f__Enterobacteriaceae;g__Escherichia-Shigella	15	0.02
MH392797.1.1488	Multiplex	d__Bacteria;p__Firmicutes;c__Bacilli;o__Lactobacillales;f__Lactobacillaceae;g__Lactobacillus;s__Lactobacillus_plantarum	d__Bacteria;p__Firmicutes;c__Bacilli;o__Lactobacillales;f__Lactobacillaceae;g__Lactobacillus	22	0.03
MG028631.1.1454	Multiplex	d__Bacteria;p__Actinobacteriota;c__Actinobacteria;o__Bifidobacteriales;f__Bifidobacteriaceae;g__Bifidobacterium;s__Bifidobacterium_sp.	d__Bacteria;p__Actinobacteriota;c__Actinobacteria;o__Bifidobacteriales;f__Bifidobacteriaceae;g__Bifidobacterium	1	0.00
HQ778752.1.1429	Multiplex	d__Bacteria;p__Bacteroidota;c__Bacteroidia;o__Bacteroidales;f__Bacteroidaceae;g__Bacteroides;s__uncultured_organism	d__Bacteria;p__Bacteroidota;c__Bacteroidia;o__Bacteroidales;f__Bacteroidaceae;g__Bacteroides	3	0.00

FN538970.134629.1361	Multiplex			6	0.01
DQ015435.1.1341_001	Multiplex			1	0.00
CP002246.166593.1680	Multiplex			8	0.01
HQ806906.1.1437	Multiplex	d__Bacteria;p__Bacteroidota;c__Bacteroidia;o__Bacteroidales;f__Bacteroidaceae;g__Bacteroides;s__uncultured_organism	d__Bacteria;p__Bacteroidota;c__Bacteroidia;o__Bacteroidales;f__Bacteroidaceae;g__Bacteroides	7	0.01
JF766405.1.1514_001	Multiplex			15596.18	18.22
EF179833.1.1502	Multiplex	d__Bacteria;p__Proteobacteria;c__Gammaproteobacteria;o__Enterobacterales;f__Enterobacteriaceae;g__Klebsiella;s__uncultured_bacterium	d__Bacteria;p__Proteobacteria;c__Gammaproteobacteria;o__Enterobacterales;f__Enterobacteriaceae;g__Klebsiella	5	0.01
JX426119.1.1502	Multiplex	d__Bacteria;p__Firmicutes;c__Bacilli;o__Lactobacillales;f__Lactobacillaceae;g__Lactobacillus;s__Lactobacillus_plantarum	d__Bacteria;p__Firmicutes;c__Bacilli;o__Lactobacillales;f__Lactobacillaceae;g__Lactobacillus	2	0.00
EU511872.1.1384_002	Multiplex			3	0.00
HQ728217.1.1500	Multiplex	d__Bacteria;p__Spirochaetota;c__Spirochaetia;o__Spirochaetales;f__Spirochaetaceae;g__Sphaerochaeta;s__uncultured_bacterium	d__Bacteria;p__Spirochaetota;c__Spirochaetia;o__Spirochaetales;f__Spirochaetaceae;g__Sphaerochaeta	1	0.00
AXLF01000003.4612176.4613708_001	Multiplex			135.98	0.16
AB573068.1.1481	Multiplex	d__Bacteria;p__Fusobacteriota;c__Fusobacteriia;o__Fusobacteriales;f__Fusobacteriaceae;g__Fusobacterium;s__Fusobacterium_nucleatum	d__Bacteria;p__Fusobacteriota;c__Fusobacteriia;o__Fusobacteriales;f__Fusobacteriaceae;g__Fusobacterium	6	0.01
KM819119.1.1536	Multiplex	d__Bacteria;p__Firmicutes;c__Bacilli;o__Lactobacillales;f__Lactobacillaceae;g__Lactobacillus;s__uncultured_Bacillus	d__Bacteria;p__Firmicutes;c__Bacilli;o__Lactobacillales;f__Lactobacillaceae;g__Lactobacillus	1	0.00
CP019469.23204.24685	Multiplex	d__Bacteria;p__Firmicutes;c__Clostridia;o__Peptostreptococcales-Tissierellales;f__Peptostreptococcaceae;g__Clostridioides;s__Clostridioides_difficile	d__Bacteria;p__Firmicutes;c__Clostridia;o__Peptostreptococcales-Tissierellales;f__Peptostreptococcaceae;g__Clostridioides	3	0.00

KX960105.1.1558	Multiplex	d__Bacteria;p__Firmicutes;c__Bacilli;o__Lactobacillales;f__Enterococcaceae;g__Enterococcus;s__Enterococcus_faecalis	d__Bacteria;p__Firmicutes;c__Bacilli;o__Lactobacillales;f__Enterococcaceae;g__Enterococcus	14	0.02
FJ975785.1.1361_001	Multiplex			63.96	0.07
EF097149.1.1403_018	Multiplex			1.16	0.00
AM180355.4007692.400	Multiplex			1	0.00
HQ777715.1.1440	Multiplex	d__Bacteria;p__Bacteroidota;c__Bacteroidia;o__Bacteroidales;f__Bacteroidaceae;g__Bacteroides;s__uncultured_organism	d__Bacteria;p__Bacteroidota;c__Bacteroidia;o__Bacteroidales;f__Bacteroidaceae;g__Bacteroides	6	0.01
AB222700.1.1488	Multiplex	d__Bacteria;p__Bacteroidota;c__Bacteroidia;o__Bacteroidales;f__Bacteroidaceae;g__Bacteroides;s__Bacteroides_finegoldii	d__Bacteria;p__Bacteroidota;c__Bacteroidia;o__Bacteroidales;f__Bacteroidaceae;g__Bacteroides	3	0.00
AB006658.1.1520	Multiplex	d__Bacteria;p__Actinobacteriota;c__Actinobacteria;o__Bifidobacteriales;f__Bifidobacteriaceae;g__Bifidobacterium;s__Bifidobacterium_breve	d__Bacteria;p__Actinobacteriota;c__Actinobacteria;o__Bifidobacteriales;f__Bifidobacteriaceae;g__Bifidobacterium	6	0.01
EU468339.1.1395	Multiplex	d__Bacteria;p__Firmicutes;c__Bacilli;o__Lactobacillales;f__Lactobacillaceae;g__Lactobacillus;s__uncultured_bacterium	d__Bacteria;p__Firmicutes;c__Bacilli;o__Lactobacillales;f__Lactobacillaceae;g__Lactobacillus	8	0.01
AM286415.1019206.102	Multiplex			3	0.00
AB616133.1.1466	Multiplex	d__Bacteria;p__Fusobacteriota;c__Fusobacteriia;o__Fusobacteriales;f__Fusobacteriaceae;g__Fusobacterium;s__Fusobacterium_simiae	d__Bacteria;p__Fusobacteriota;c__Fusobacteriia;o__Fusobacteriales;f__Fusobacteriaceae;g__Fusobacterium	5	0.01
X73450.1.1470	Multiplex	d__Bacteria;p__Firmicutes;c__Clostridia;o__Peptostreptococcales-Tissierellales;f__Peptostreptococcaceae;g__Clostridioides;s__Clostridioides_difficile	d__Bacteria;p__Firmicutes;c__Clostridia;o__Peptostreptococcales-Tissierellales;f__Peptostreptococcaceae;g__Clostridioides	12	0.01
CP025044.39590.41097	Multiplex	d__Bacteria;p__Firmicutes;c__Clostridia;o__Peptostreptococcales-Tissierellales;f__Peptostreptococcaceae;g__Clostridioides;s__Clostridioides_difficile	d__Bacteria;p__Firmicutes;c__Clostridia;o__Peptostreptococcales-Tissierellales;f__Peptostreptococcaceae;g__Clostridioides	43	0.05

EU773007.1.1247	Multiplex	d__Bacteria;p__Actinobacteriota;c__Coriobacteriia;o__Coriobacteriales;f__Eggerthellaceae;g__Enterorhabdus;s__uncultured_bacterium	d__Bacteria;p__Actinobacteriota;c__Coriobacteriia;o__Coriobacteriales;f__Eggerthellaceae;g__Enterorhabdus	8	0.01
KF066950.1.1341	Multiplex	d__Bacteria;p__Fusobacteriota;c__Fusobacteriia;o__Fusobacteriales;f__Fusobacteriaceae;g__Fusobacterium;s__uncultured_bacterium	d__Bacteria;p__Fusobacteriota;c__Fusobacteriia;o__Fusobacteriales;f__Fusobacteriaceae;g__Fusobacterium	18	0.02
EU776680.1.1401	Multiplex	d__Bacteria;p__Proteobacteria;c__Gammaproteobacteria;o__Enterobacterales;f__Enterobacteriaceae;g__Escherichia-Shigella;s__uncultured_bacterium	d__Bacteria;p__Proteobacteria;c__Gammaproteobacteria;o__Enterobacterales;f__Enterobacteriaceae;g__Escherichia-Shigella	4	0.00
KF842515.1.1412	Multiplex	d__Bacteria;p__Proteobacteria;c__Gammaproteobacteria;o__Enterobacterales;f__Enterobacteriaceae;g__Escherichia-Shigella;s__uncultured_bacterium	d__Bacteria;p__Proteobacteria;c__Gammaproteobacteria;o__Enterobacterales;f__Enterobacteriaceae;g__Escherichia-Shigella	21	0.02
KX057696.1.1515_002	Multiplex			3479.3	4.06
HL282422.2.1545	Multiplex	d__Bacteria;p__Fusobacteriota;c__Fusobacteriia;o__Fusobacteriales;f__Fusobacteriaceae;g__Fusobacterium;s__unidentified	d__Bacteria;p__Fusobacteriota;c__Fusobacteriia;o__Fusobacteriales;f__Fusobacteriaceae;g__Fusobacterium	10	0.01
AGRM01000003.578375.	Multiplex			5	0.01
KJ722780.1.1464	Multiplex	d__Bacteria;p__Firmicutes;c__Bacilli;o__Lactobacillales;f__Lactobacillaceae;g__Lactobacillus;s__Lactobacillus_plantarum	d__Bacteria;p__Firmicutes;c__Bacilli;o__Lactobacillales;f__Lactobacillaceae;g__Lactobacillus	3	0.00
EU775673.1.1399	Multiplex	d__Bacteria;p__Proteobacteria;c__Gammaproteobacteria;o__Enterobacterales;f__Enterobacteriaceae;g__Escherichia-Shigella;s__uncultured_bacterium	d__Bacteria;p__Proteobacteria;c__Gammaproteobacteria;o__Enterobacterales;f__Enterobacteriaceae;g__Escherichia-Shigella	76	0.09
KJ123815.1.1513_001	Multiplex			0.33	0.00
AY959011.1.1496_001	Multiplex			0.08	0.00
CP009787.3194722.319	Multiplex			2	0.00
EU828415.1.1454	Multiplex	d__Bacteria;p__Proteobacteria;c__Gammaproteobacteria;o__Burkholderia	d__Bacteria;p__Proteobacteria;c__Gammaproteobacteria;o__Burkholderia	7	0.01

		les;f__Oxalobacteraceae;g__Janthinobacterium;s__uncultured_bacterium	les;f__Oxalobacteraceae;g__Janthinobacterium		
HM557383.1.1371	Multiplex	d__Bacteria;p__Proteobacteria;c__Gammaproteobacteria;o__Enterobacterales;f__Enterobacteriaceae;g__Enterobacter;s__uncultured_bacterium	d__Bacteria;p__Proteobacteria;c__Gammaproteobacteria;o__Enterobacterales;f__Enterobacteriaceae;g__Enterobacter	3	0.00
EU778456.1.1401	Multiplex	d__Bacteria;p__Proteobacteria;c__Gammaproteobacteria;o__Enterobacterales;f__Enterobacteriaceae;g__Escherichia-Shigella;s__uncultured_bacterium	d__Bacteria;p__Proteobacteria;c__Gammaproteobacteria;o__Enterobacterales;f__Enterobacteriaceae;g__Escherichia-Shigella	32	0.04
EU560820.1.1485	Multiplex	d__Bacteria;p__Proteobacteria;c__Gammaproteobacteria;o__Enterobacterales;f__Enterobacteriaceae;g__Escherichia-Shigella;s__uncultured_bacterium	d__Bacteria;p__Proteobacteria;c__Gammaproteobacteria;o__Enterobacterales;f__Enterobacteriaceae;g__Escherichia-Shigella	69	0.08
CP019858.3937614.393	Multiplex			101	0.12
KP256081.1.1512	Multiplex	d__Bacteria;p__Firmicutes;c__Bacilli;o__Lactobacillales;f__Lactobacillaceae;g__Lactobacillus;s__Lactobacillus_pentosus	d__Bacteria;p__Firmicutes;c__Bacilli;o__Lactobacillales;f__Lactobacillaceae;g__Lactobacillus	30	0.04
AY081984.1.1503	Multiplex	d__Bacteria;p__Proteobacteria;c__Gammaproteobacteria;o__Pseudomonadales;f__Pseudomonadaceae;g__Pseudomonas;s__uncultured_bacterium	d__Bacteria;p__Proteobacteria;c__Gammaproteobacteria;o__Pseudomonadales;f__Pseudomonadaceae;g__Pseudomonas	3	0.00
AY850535.1.1224	Multiplex	d__Bacteria;p__Actinobacteriota;c__Actinobacteria;o__Bifidobacteriales;f__Bifidobacteriaceae;g__Bifidobacterium;s__uncultured_bacterium	d__Bacteria;p__Actinobacteriota;c__Actinobacteria;o__Bifidobacteriales;f__Bifidobacteriaceae;g__Bifidobacterium	2	0.00
HQ745182.1.1434	Multiplex	d__Bacteria;p__Bacteroidota;c__Bacteroidia;o__Bacteroidales;f__Bacteroidaceae;g__Bacteroides;s__uncultured_organism	d__Bacteria;p__Bacteroidota;c__Bacteroidia;o__Bacteroidales;f__Bacteroidaceae;g__Bacteroides	5	0.01
JQ467938.1.1393	Multiplex	d__Bacteria;p__Fusobacteriota;c__Fusobacteriia;o__Fusobacteriales;f__Leptotrichiaceae;g__Leptotrichia;s__uncultured_bacterium	d__Bacteria;p__Fusobacteriota;c__Fusobacteriia;o__Fusobacteriales;f__Leptotrichiaceae;g__Leptotrichia	90	0.11
KX957774.1.1421	Multiplex	d__Bacteria;p__Proteobacteria;c__Gammaproteobacteria;o__Enterobacterales;f__Enterobacteriaceae;g__Enterobacter;s__Enterobacter_cloacae	d__Bacteria;p__Proteobacteria;c__Gammaproteobacteria;o__Enterobacterales;f__Enterobacteriaceae;g__Enterobacter	5	0.01

HQ790343.1.1432	Multiplex	d__Bacteria;p__Bacteroidota;c__Bacteroidia;o__Bacteroidales;f__Bacteroidaceae;g__Bacteroides;s__uncultured_organism	d__Bacteria;p__Bacteroidota;c__Bacteroidia;o__Bacteroidales;f__Bacteroidaceae;g__Bacteroides	6	0.01
DQ014744.1.1338	Multiplex	d__Bacteria;p__Firmicutes;c__Clostridia;o__Lachnospirales;f__Lachnospiraceae;g__Lachnoclostridium;s__uncultured_bacterium	d__Bacteria;p__Firmicutes;c__Clostridia;o__Lachnospirales;f__Lachnospiraceae;g__Lachnoclostridium	1	0.00
EF097675.1.1407	Multiplex	d__Bacteria;p__Bacteroidota;c__Bacteroidia;o__Bacteroidales;f__Muribaculaceae;g__Muribaculaceae;s__uncultured_bacterium	d__Bacteria;p__Bacteroidota;c__Bacteroidia;o__Bacteroidales;f__Muribaculaceae;g__Muribaculaceae	1	0.00
HQ819022.1.1439_001	Multiplex			8616.22	10.07
DQ441256.1.1313	Multiplex	d__Bacteria;p__Bacteroidota;c__Bacteroidia;o__Bacteroidales;f__Rikenellaceae;g__Alistipes;s__uncultured_bacterium	d__Bacteria;p__Bacteroidota;c__Bacteroidia;o__Bacteroidales;f__Rikenellaceae;g__Alistipes	7	0.01
EU451156.1.1374_009	Multiplex			1	0.00
HQ819061.1.1438	Multiplex	d__Bacteria;p__Fusobacteriota;c__Fusobacteriia;o__Fusobacteriales;f__Fusobacteriaceae;g__Fusobacterium;s__uncultured_organism	d__Bacteria;p__Fusobacteriota;c__Fusobacteriia;o__Fusobacteriales;f__Fusobacteriaceae;g__Fusobacterium	4	0.00
EU622695.1.1497_058	Multiplex			1	0.00
JF121027.1.1363	Multiplex	d__Bacteria;p__Proteobacteria;c__Gammaproteobacteria;o__Enterobacterales;f__Yersiniaceae;g__Serratia;s__uncultured_bacterium	d__Bacteria;p__Proteobacteria;c__Gammaproteobacteria;o__Enterobacterales;f__Yersiniaceae;g__Serratia	2	0.00
EF603126.1.1475_079	Multiplex			3.99	0.00
CP009367.4195509.419	Multiplex			3	0.00
EF179121.1.1461	Multiplex	d__Bacteria;p__Proteobacteria;c__Gammaproteobacteria;o__Enterobacterales;f__Yersiniaceae;g__Yersinia;s__Yersinia_bercovieri	d__Bacteria;p__Proteobacteria;c__Gammaproteobacteria;o__Enterobacterales;f__Yersiniaceae;g__Yersinia	7	0.01
KM819129.1.1528	Multiplex	d__Bacteria;p__Firmicutes;c__Bacilli;o__Lactobacillales;f__Lactobacillaceae;g__Lactobacillus;s__uncultured_Bacillus	d__Bacteria;p__Firmicutes;c__Bacilli;o__Lactobacillales;f__Lactobacillaceae;g__Lactobacillus	6	0.01

DQ002385.1.1486					Multiplex			d__Bacteria;p__Proteobacteria;c__Gammaproteobacteria;o__Enterobacterales;f__Yersiniaceae;g__Serratia;s__Serratia_marcescens	d__Bacteria;p__Proteobacteria;c__Gammaproteobacteria;o__Enterobacterales;f__Yersiniaceae;g__Serratia	2	0.00
EU778438.1.1420					Multiplex			d__Bacteria;p__Firmicutes;c__Bacilli;o__Lactobacillales;f__Enterococcaceae;g__Enterococcus;s__uncultured_bacterium	d__Bacteria;p__Firmicutes;c__Bacilli;o__Lactobacillales;f__Enterococcaceae;g__Enterococcus	7	0.01
CP010888.654100.6556					Multiplex					66	0.08
HQ819061.1.1438_001					Multiplex					10.78	0.01
EF406402.1.1499_010					Multiplex					0.02	0.00
HQ810939.1.1381					Multiplex			d__Bacteria;p__Bacteroidota;c__Bacteroidia;o__Bacteroidales;f__Bacteroidaceae;g__Bacteroides;s__uncultured_organism	d__Bacteria;p__Bacteroidota;c__Bacteroidia;o__Bacteroidales;f__Bacteroidaceae;g__Bacteroides	8	0.01
EU560876.1.1492					Multiplex			d__Bacteria;p__Firmicutes;c__Bacilli;o__Lactobacillales;f__Enterococcaceae;g__Enterococcus;s__uncultured_bacterium	d__Bacteria;p__Firmicutes;c__Bacilli;o__Lactobacillales;f__Enterococcaceae;g__Enterococcus	9	0.01
CDQP01001253.198.170					Multiplex					5	0.01
KC936918.1.1479					Multiplex			d__Bacteria;p__Fusobacteriota;c__Fusobacteriia;o__Fusobacteriales;f__Fusobacteriaceae;g__Fusobacterium;s__uncultured_bacterium	d__Bacteria;p__Fusobacteriota;c__Fusobacteriia;o__Fusobacteriales;f__Fusobacteriaceae;g__Fusobacterium	3	0.00
EU778438.1.1420_005					Multiplex					103.82	0.12
HQ800646.1.1455					Multiplex			d__Bacteria;p__Bacteroidota;c__Bacteroidia;o__Bacteroidales;f__Bacteroidaceae;g__Bacteroides;s__uncultured_organism	d__Bacteria;p__Bacteroidota;c__Bacteroidia;o__Bacteroidales;f__Bacteroidaceae;g__Bacteroides	1	0.00
09a8fa70427aa68f16934c729b826071	1eb3021faaec49c2_1	99.454	3660889	3659426	Full-length	Escherichia_coli_ATCC_700926	True		d__Bacteria;p__Proteobacteria;c__Gammaproteobacteria;o__Enterobacterales;f__Enterobacteriaceae;g__Escherichia-Shigella	308	0.99
4bec1cb362f10c0518dae936b3032c6f	38ad2dab3e5e43b7_1	99.932	627724	629197	Full-length	Staphylococcus_aureus_subsp_aureus_ATCC_BAA_1556	True		d__Bacteria;p__Firmicutes;c__Bacilli;o__Staphylococcales;f__Staphylococcaceae;g__Staphylococcus	224	0.72

51d51e872348c3c5e3b70be7f91c4a32	071a82bb90b94c6f_1	100	494810	496268	Full-length	Acinetobacter_baumannii_ATCC_17978	True	d__Bacteria;p__Proteobacteria;c__Gammaproteobacteria;o__Pseudomonadales;f__Moraxellaceae;g__Acinetobacter;s__Acinetobacter_baumannii	d__Bacteria;p__Proteobacteria;c__Gammaproteobacteria;o__Pseudomonadales;f__Moraxellaceae;g__Acinetobacter	2360	7.60
cd1af128eb857acddb168e57245021ec	38ad2dab3e5e43b7_1	99.796	627724	629197	Full-length	Staphylococcus_aureus_subsp_aureus_ATCC_BAA_1556	True		d__Bacteria;p__Firmicutes;c__Bacilli;o__Staphylococcales;f__Staphylococcaceae;g__Staphylococcus	212	0.68
d5ebbbf0f104adc463156fca557bb6fc					Full-length			d__Bacteria;p__Verrucomicrobiota;c__Verrucomicrobiae;o__Verrucomicrobiales;f__Akkermansiaceae;g__Akkermansia;s__Akkermansia_muciniphila	d__Bacteria;p__Verrucomicrobiota;c__Verrucomicrobiae;o__Verrucomicrobiales;f__Akkermansiaceae;g__Akkermansia	12	0.04
7983269916ac14fd4655f0a0c60f8694	9dc637a985b042c4_1	100	605244	606690	Full-length	Cutibacterium_acnes_ATCC_11828	True		d__Bacteria;p__Actinobacteriota;c__Actinobacteria;o__Propionibacteriales;f__Propionibacteriaceae;g__Cutibacterium	958	3.08
d8a3399acd76da73c1b2b8a3683a7540					Full-length				d__Bacteria;p__Cyanobacteria;c__Cyanobacteriia;o__Chloroplast;f__Chloroplast;g__Chloroplast	24	0.08
c0089458dae2c0559162e58eb2661873	1eb3021faaec49c2_1	100	454995	456458	Full-length	Escherichia_coli_ATCC_700926	True		d__Bacteria;p__Proteobacteria;c__Gammaproteobacteria;o__Enterobacterales;f__Enterobacteriaceae;g__Escherichia-Shigella	281	0.90
a0530711bdec8d611872863731f96dde	adb6d513b7374f84_1	100	16704	18182	Full-length	Streptococcus_mutans_ATCC_700610	True	d__Bacteria;p__Firmicutes;c__Bacilli;o__Lactobacillales;f__Streptococcaceae;g__Streptococcus;s__Streptococcus_mutans	d__Bacteria;p__Firmicutes;c__Bacilli;o__Lactobacillales;f__Streptococcaceae;g__Streptococcus	1293	4.16
994cb47a2b400e6185743e1765244faf	38ad2dab3e5e43b7_1	99.932	627724	629197	Full-length	Staphylococcus_aureus_subsp_aureus_ATCC_BAA_1556	True		d__Bacteria;p__Firmicutes;c__Bacilli;o__Staphylococcales;f__Staphylococcaceae;g__Staphylococcus	214	0.69
13bc41e35faa4f40a01de5a972c0c4ac	1eb3021faaec49c2_1	100	3660889	3659426	Full-length	Escherichia_coli_ATCC_700926	True		d__Bacteria;p__Proteobacteria;c__Gammaproteobacteria;o__Enterobacterales;f__Enterobacteriaceae;g__Escherichia-Shigella	611	1.97
9fc4609d84db9e441de7db46d7172ae4	1eb3021faaec49c2_1	100	1152600	1154063	Full-length	Escherichia_coli_ATCC_700926	True		d__Bacteria;p__Proteobacteria;c__Gammaproteobacteria;o__Enterobacterales;f__Enterobacteriaceae;g__Escherichia-Shigella	256	0.82

0bf3e0641c88ec588bc66b567c3fa63d	1eb3021faaec49c2_1	99.932	3660889	3659426	Full-length	Escherichia_coli_ATCC_700926	True		d__Bacteria;p__Proteobacteria;c__Gammaproteobacteria;o__Enterobacterales;f__Enterobacteriaceae;g__Escherichia-Shigella	285	0.92
9eda36e35469eb0ba4b8045fa7acabd0	d889f9dd971b473d_1	99.862	2587739	2589188	Full-length	Phocaeicola_vulgatus_ATCC_8482	True	d__Bacteria;p__Bacteroidota;c__Bacteroidia;o__Bacteroidales;f__Bacteroidaceae;g__Bacteroides;s__Bacteroides_vulgatus	d__Bacteria;p__Bacteroidota;c__Bacteroidia;o__Bacteroidales;f__Bacteroidaceae;g__Bacteroides	274	0.88
339aac7377a0d948a52bc0b2bf3dfda8	d889f9dd971b473d_1	100	2095461	2096910	Full-length	Phocaeicola_vulgatus_ATCC_8482	True	d__Bacteria;p__Bacteroidota;c__Bacteroidia;o__Bacteroidales;f__Bacteroidaceae;g__Bacteroides;s__Bacteroides_vulgatus	d__Bacteria;p__Bacteroidota;c__Bacteroidia;o__Bacteroidales;f__Bacteroidaceae;g__Bacteroides	350	1.13
e7b11bfbee47d834b64203b6c1294201	3042186b85304e8d_1	99.93	2256227	2257660	Full-length	Clostridium_beijerinckii_ATCC_35702	True	d__Bacteria;p__Firmicutes;c__Clostridia;o__Clostridiales;f__Clostridiaceae;g__Clostridium_sensu_stricto_1;s__Clostridium_beijerinckii	d__Bacteria;p__Firmicutes;c__Clostridia;o__Clostridiales;f__Clostridiaceae;g__Clostridium_sensu_stricto_1	153	0.49
2f66c5bd318dfac1e9738ffd54ca88b8	3042186b85304e8d_1	100	5769886	5768453	Full-length	Clostridium_beijerinckii_ATCC_35702	True	d__Bacteria;p__Firmicutes;c__Clostridia;o__Clostridiales;f__Clostridiaceae;g__Clostridium_sensu_stricto_1;s__Clostridium_beijerinckii	d__Bacteria;p__Firmicutes;c__Clostridia;o__Clostridiales;f__Clostridiaceae;g__Clostridium_sensu_stricto_1	304	0.98
a0ec2668157ffb2374ece8cad58ecc5f	285a115d88c043f1_1	100	678369	679826	Full-length	Pseudomonas_aeruginosa_ATCC_9027	True	d__Bacteria;p__Proteobacteria;c__Gammaproteobacteria;o__Pseudomonadales;f__Pseudomonadaceae;g__Pseudomonas;s__Pseudomonas_aeruginosa	d__Bacteria;p__Proteobacteria;c__Gammaproteobacteria;o__Pseudomonadales;f__Pseudomonadaceae;g__Pseudomonas	630	2.03
0ed2d53fd823b2ef922b6f349cade1cd	045df7c7cb0f42b5_1	100	1223511	1222049	Full-length	Neisseria_meningitidis_ATCC_BAA_335	True	d__Bacteria;p__Proteobacteria;c__Gammaproteobacteria;o__Burkholderiales;f__Neisseriaceae;g__Neisseria;s__Neisseria_meningitidis	d__Bacteria;p__Proteobacteria;c__Gammaproteobacteria;o__Burkholderiales;f__Neisseriaceae;g__Neisseria	2291	7.37
7dcace79bc8188077e07b0085aa57b7f	5371a9904eb64361_1	100	8928	10400	Full-length	Bacillus_pacificus_ATCC_10987	True		d__Bacteria;p__Firmicutes;c__Bacilli;o__Bacillales;f__Bacillaceae;g__Bacillus	496	1.60
aab4d379026eb5e6a7e2b32ec4c715e7	f5bcb58692924cb7_1	99.796	1722262	1720790	Full-length	Staphylococcus_epidermidis_ATCC_12228	True		d__Bacteria;p__Firmicutes;c__Bacilli;o__Staphylococcales;f__Staphylococcaceae;g__Staphylococcus	307	0.99
efe5e4e4ba300f45c88da09e3e9cd3e4	285a115d88c043f1_1	100	5383111	5381654	Full-length	Pseudomonas_aeruginosa_ATCC_9027	True	d__Bacteria;p__Proteobacteria;c__Gammaproteobacteria;o__Pseudomonadales;f__Pseudomonadaceae;g__Pseudomonas;s__Pseudomonas_aeruginosa	d__Bacteria;p__Proteobacteria;c__Gammaproteobacteria;o__Pseudomonadales;f__Pseudomonadaceae;g__Pseudomonas	216	0.70

ad20976e0e8c7e5adcb7f11b5fcf7830	d889f9dd971b473d_1	99.862	4431943	4430494	Full-length	Phocaeicola_vulgatus_ATCC_8482	True	d__Bacteria;p__Bacteroidota;c__Bacteroidia;o__Bacteroidales;f__Bacteroidaceae;g__Bacteroides;s__Bacteroides_vulgatus	d__Bacteria;p__Bacteroidota;c__Bacteroidia;o__Bacteroidales;f__Bacteroidaceae;g__Bacteroides	271	0.87
d5cb708aa8b5ddc54ca7e0528124fe20	2b7fa0e1de5f42de_1	100	2562488	2561065	Full-length	Deinococcus_radiodurans_ATCC_BAA_816	True	d__Bacteria;p__Deinococcota;c__Deinococci;o__Deinococcales;f__Deinococcaceae;g__Deinococcus;s__Deinococcus_radiodurans	d__Bacteria;p__Deinococcota;c__Deinococci;o__Deinococcales;f__Deinococcaceae;g__Deinococcus	374	1.20
34b695df3f78278d604299f2c4adc80c	2b7fa0e1de5f42de_1	100	182548	183971	Full-length	Deinococcus_radiodurans_ATCC_BAA_816	True	d__Bacteria;p__Deinococcota;c__Deinococci;o__Deinococcales;f__Deinococcaceae;g__Deinococcus;s__Deinococcus_radiodurans	d__Bacteria;p__Deinococcota;c__Deinococci;o__Deinococcales;f__Deinococcaceae;g__Deinococcus	1031	3.32
3feab8f97f15836d17829bc4ea074162	3042186b85304e8d_1	99.93	445874	447307	Full-length	Clostridium_beijerinckii_ATCC_35702	True	d__Bacteria;p__Firmicutes;c__Clostridia;o__Clostridiales;f__Clostridiaceae;g__Clostridium_sensu_stricto_1;s__Clostridium_beijerinckii	d__Bacteria;p__Firmicutes;c__Clostridia;o__Clostridiales;f__Clostridiaceae;g__Clostridium_sensu_stricto_1	175	0.56
b2ef16dee036f7ce8b9b74a7234111c1	3042186b85304e8d_1	99.861	3404822	3403389	Full-length	Clostridium_beijerinckii_ATCC_35702	True	d__Bacteria;p__Firmicutes;c__Clostridia;o__Clostridiales;f__Clostridiaceae;g__Clostridium_sensu_stricto_1;s__Clostridium_beijerinckii	d__Bacteria;p__Firmicutes;c__Clostridia;o__Clostridiales;f__Clostridiaceae;g__Clostridium_sensu_stricto_1	144	0.46
9f3938b0148e6fa857077444480b6fd3	d889f9dd971b473d_1	99.724	4744605	4743156	Full-length	Phocaeicola_vulgatus_ATCC_8482	True	d__Bacteria;p__Bacteroidota;c__Bacteroidia;o__Bacteroidales;f__Bacteroidaceae;g__Bacteroides;s__Bacteroides_vulgatus	d__Bacteria;p__Bacteroidota;c__Bacteroidia;o__Bacteroidales;f__Bacteroidaceae;g__Bacteroides	257	0.83
aaf82c612373bafcef3509b72ee24809	3042186b85304e8d_1	99.93	5769886	5768453	Full-length	Clostridium_beijerinckii_ATCC_35702	True	d__Bacteria;p__Firmicutes;c__Clostridia;o__Clostridiales;f__Clostridiaceae;g__Clostridium_sensu_stricto_1;s__Clostridium_beijerinckii	d__Bacteria;p__Firmicutes;c__Clostridia;o__Clostridiales;f__Clostridiaceae;g__Clostridium_sensu_stricto_1	194	0.62
61de72098cbf9f693f09c863d1c5a586	2504aeb524e74d25_1	100	229338	230791	Full-length	Porphyromonas_gingivalis_ATCC_33277	True	d__Bacteria;p__Bacteroidota;c__Bacteroidia;o__Bacteroidales;f__Porphyromonadaceae;g__Porphyromonas;s__Porphyromonas_gingivalis	d__Bacteria;p__Bacteroidota;c__Bacteroidia;o__Bacteroidales;f__Porphyromonadaceae;g__Porphyromonas	2925	9.41
a8f0a9703e902a87d6be02364e684c50	3042186b85304e8d_1	100	5775823	5774390	Full-length	Clostridium_beijerinckii_ATCC_35702	True	d__Bacteria;p__Firmicutes;c__Clostridia;o__Clostridiales;f__Clostridiaceae;g__Clostridium_sensu_stricto_1;s__Clostridium_beijerinckii	d__Bacteria;p__Firmicutes;c__Clostridia;o__Clostridiales;f__Clostridiaceae;g__Clostridium_sensu_stricto_1	215	0.69
913abfe7ecfd10c4eb54ea1dea67f07c	4762fdd895094939_1	100	96390	97815	Full-length	Helicobacter_pylori_ATCC_700392	True	d__Bacteria;p__Campilobacterota;c__Campylobacteria;o__Campylobacterales;f__Helicobacteraceae;g__Helicobacter;s__Helicobacter_pylori	d__Bacteria;p__Campilobacterota;c__Campylobacteria;o__Campylobacterales;f__Helicobacteraceae;g__Helicobacter	1125	3.62

b56b7466c84c7ca53aa71e4ce25f61b0	c45a1c57adf4460e_2	100	23119	24570	Full-length	Bifidobacterium_adolescentis_ATCC_15703	True	d__Bacteria;p__Actinobacteriota;c__Actinobacteria;o__Bifidobacteriales;f__Bifidobacteriaceae;g__Bifidobacterium;s__Bifidobacterium_adolescentis	d__Bacteria;p__Actinobacteriota;c__Actinobacteria;o__Bifidobacteriales;f__Bifidobacteriaceae;g__Bifidobacterium	447	1.44
0ccaf57fc10e3ca943b9911e91c05de1	3042186b85304e8d_1	100	2267882	2269315	Full-length	Clostridium_beijerinckii_ATCC_35702	True	d__Bacteria;p__Firmicutes;c__Clostridia;o__Clostridiales;f__Clostridiaceae;g__Clostridium_sensu_stricto_1;s__Clostridium_beijerinckii	d__Bacteria;p__Firmicutes;c__Clostridia;o__Clostridiales;f__Clostridiaceae;g__Clostridium_sensu_stricto_1	170	0.55
d669559001b98da82874fac6581afc1c	3042186b85304e8d_1	100	4548532	4547099	Full-length	Clostridium_beijerinckii_ATCC_35702	True	d__Bacteria;p__Firmicutes;c__Clostridia;o__Clostridiales;f__Clostridiaceae;g__Clostridium_sensu_stricto_1;s__Clostridium_beijerinckii	d__Bacteria;p__Firmicutes;c__Clostridia;o__Clostridiales;f__Clostridiaceae;g__Clostridium_sensu_stricto_1	171	0.55
21b49156bf40185385c31d7db91a5ad5	5371a9904eb64361_1	100	325854	327326	Full-length	Bacillus_pacificus_ATCC_10987	True		d__Bacteria;p__Firmicutes;c__Bacilli;o__Bacillales;f__Bacillaceae;g__Bacillus	62	0.20
56fa3c3172361f91fadde5f221e6f5b4	c45a1c57adf4460e_2	99.243	23119	24570	Full-length	Bifidobacterium_adolescentis_ATCC_15703	True	d__Bacteria;p__Actinobacteriota;c__Actinobacteria;o__Bifidobacteriales;f__Bifidobacteriaceae;g__Bifidobacterium;s__Bifidobacterium_adolescentis	d__Bacteria;p__Actinobacteriota;c__Actinobacteria;o__Bifidobacteriales;f__Bifidobacteriaceae;g__Bifidobacterium	119	0.38
26cfdb2bcbd3a64a84e9b237d94a97eb	27b35dd1651e48dd_1	100	213391	214873	Full-length	Enterococcus_faecalis_ATCC_47077	True	d__Bacteria;p__Firmicutes;c__Bacilli;o__Lactobacillales;f__Enterococcaceae;g__Enterococcus;s__Enterococcus_faecalis	d__Bacteria;p__Firmicutes;c__Bacilli;o__Lactobacillales;f__Enterococcaceae;g__Enterococcus	738	2.38
07b2978940b50886f3866b04b7f982cf	58ae911973b74ffc_1	100	16330	17800	Full-length	Streptococcus_agalactiae_ATCC_BAA_611	True	d__Bacteria;p__Firmicutes;c__Bacilli;o__Lactobacillales;f__Streptococcaceae;g__Streptococcus;s__Streptococcus_agalactiae	d__Bacteria;p__Firmicutes;c__Bacilli;o__Lactobacillales;f__Streptococcaceae;g__Streptococcus	2250	7.24
7932226c8cd025d2ea117b03d48f63e8	3042186b85304e8d_1	99.93	9364	10797	Full-length	Clostridium_beijerinckii_ATCC_35702	True	d__Bacteria;p__Firmicutes;c__Clostridia;o__Clostridiales;f__Clostridiaceae;g__Clostridium_sensu_stricto_1;s__Clostridium_beijerinckii	d__Bacteria;p__Firmicutes;c__Clostridia;o__Clostridiales;f__Clostridiaceae;g__Clostridium_sensu_stricto_1	190	0.61
34babcf1732962962d2fbbbadf3af637	f5bcb58692924cb7_1	100	1597980	1596508	Full-length	Staphylococcus_epidermidis_ATCC_12228	True		d__Bacteria;p__Firmicutes;c__Bacilli;o__Staphylococcales;f__Staphylococcaceae;g__Staphylococcus	190	0.61
a7e2262894c30111bfd4daee5736414f	f5bcb58692924cb7_1	99.932	1597980	1596508	Full-length	Staphylococcus_epidermidis_	True		d__Bacteria;p__Firmicutes;c__Bacilli;o__Staphylococcales;f__Staphylococcaceae;g__Staphylococcus	37	0.12

						ATCC_12228					
3cc006600b219858ac3c647f2613a32a	d889f9dd971b473d_1	99.931	348208	346759	Full-length	Phocaeicola_vulgatus_ATCC_8482	True	d__Bacteria;p__Bacteroidota;c__Bacteroidia;o__Bacteroidales;f__Bacteroidaceae;g__Bacteroides;s__Bacteroides_vulgatus	d__Bacteria;p__Bacteroidota;c__Bacteroidia;o__Bacteroidales;f__Bacteroidaceae;g__Bacteroides	568	1.83
f3cfb383dcfb753cad226072f6665784	f5bcb58692924cb7_1	100	2335912	2334440	Full-length	Staphylococcus_epidermidis_ATCC_12228	True		d__Bacteria;p__Firmicutes;c__Bacilli;o__Staphylococcales;f__Staphylococcaceae;g__Staphylococcus	648	2.09
dd586a1ae04ed2d129d944286ae71f61	f5bcb58692924cb7_1	100	1722262	1720790	Full-length	Staphylococcus_epidermidis_ATCC_12228	True		d__Bacteria;p__Firmicutes;c__Bacilli;o__Staphylococcales;f__Staphylococcaceae;g__Staphylococcus	410	1.32
a5ebdb1225c13ba7cfd2467533fc6da6	c45a1c57adf4460e_1	100	1850147	1848699	Full-length	Bifidobacterium_adolescentis_ATCC_15703	True	d__Bacteria;p__Actinobacteriota;c__Actinobacteria;o__Bifidobacteriales;f__Bifidobacteriaceae;g__Bifidobacterium;s__Bifidobacterium_adolescentis	d__Bacteria;p__Actinobacteriota;c__Actinobacteria;o__Bifidobacteriales;f__Bifidobacteriaceae;g__Bifidobacterium	110	0.35
8348a732f75c6a0439af2ebffbac5ff1	3042186b85304e8d_1	99.861	2030991	2032424	Full-length	Clostridium_beijerinckii_ATCC_35702	True	d__Bacteria;p__Firmicutes;c__Clostridia;o__Clostridiales;f__Clostridiaceae;g__Clostridium_sensu_stricto_1;s__Clostridium_beijerinckii	d__Bacteria;p__Firmicutes;c__Clostridia;o__Clostridiales;f__Clostridiaceae;g__Clostridium_sensu_stricto_1	140	0.45
4c67b666ce2da2054aec0ba50dd1e494	d889f9dd971b473d_1	99.931	2095461	2096910	Full-length	Phocaeicola_vulgatus_ATCC_8482	True	d__Bacteria;p__Bacteroidota;c__Bacteroidia;o__Bacteroidales;f__Bacteroidaceae;g__Bacteroides;s__Bacteroides_vulgatus	d__Bacteria;p__Bacteroidota;c__Bacteroidia;o__Bacteroidales;f__Bacteroidaceae;g__Bacteroides	78	0.25
fce86ef2355971d83413788cbb660531	5964adb8d0df4fde_1	100	477470	478964	Full-length	Lactobacillus_gasseri_ATCC_33323	True	d__Bacteria;p__Firmicutes;c__Bacilli;o__Lactobacillales;f__Lactobacillaceae;g__Lactobacillus;s__Lactobacillus_gasseri	d__Bacteria;p__Firmicutes;c__Bacilli;o__Lactobacillales;f__Lactobacillaceae;g__Lactobacillus	2102	6.77
04d3de55f475a0888205c9507901a096	89e35fec14744942_1	100	528332	529794	Full-length	Schaalia_odontolytica_ATCC_17982	True	d__Bacteria;p__Actinobacteriota;c__Actinobacteria;o__Actinomycetales;f__Actinomycetaceae;g__Actinomyces;s__Schaalia_odontolytica	d__Bacteria;p__Actinobacteriota;c__Actinobacteria;o__Actinomycetales;f__Actinomycetaceae;g__Actinomyces	112	0.36
021408f68ac3d943f7e16261373b2a61	3042186b85304e8d_1	99.93	924660	926093	Full-length	Clostridium_beijerinckii_ATCC_35702	True	d__Bacteria;p__Firmicutes;c__Clostridia;o__Clostridiales;f__Clostridiaceae;g__Clostridium_sensu_stricto_1;s__Clostridium_beijerinckii	d__Bacteria;p__Firmicutes;c__Clostridia;o__Clostridiales;f__Clostridiaceae;g__Clostridium_sensu_stricto_1	164	0.53
bc7d5e82dae59e2e5e8321bb5c5eae4b	adb6d513b7374f84_1	100	1660154	1658676	Full-length	Streptococcus_muta	True	d__Bacteria;p__Firmicutes;c__Bacilli;o__Lactobacillales;f__Streptococcace	d__Bacteria;p__Firmicutes;c__Bacilli;o__Lactobacillales;f__Streptococcaceae;g__Streptococcus	903	2.91

						ns_ATCC_700610		ae;g__Streptococcus;s__Streptococcus_mutans			
1f1158dbb4126e5a7f04094b61700493	3042186b85304e8d_1	100	142194	143627	Full-length	Clostridium_beijerinckii_ATCC_35702	True	d__Bacteria;p__Firmicutes;c__Clostridia;o__Clostridiales;f__Clostridiaceae;g__Clostridium_sensu_stricto_1;s__Clostridium_beijerinckii	d__Bacteria;p__Firmicutes;c__Clostridia;o__Clostridiales;f__Clostridiaceae;g__Clostridium_sensu_stricto_1	292	0.94
985d4a0d4d7e7d255af6a92044ae5901	38ad2dab3e5e43b7_1	99.932	627724	629197	Full-length	Staphylococcus_aureus_subsp_aureus_ATCC_BAA_1556	True		d__Bacteria;p__Firmicutes;c__Bacilli;o__Staphylococcales;f__Staphylococcaceae;g__Staphylococcus	233	0.75
92bcc461c3dbfbeb88759ada02f95a6b	5371a9904eb64361_1	100	616926	618398	Full-length	Bacillus_pacificus_ATCC_10987	True		d__Bacteria;p__Firmicutes;c__Bacilli;o__Bacillales;f__Bacillaceae;g__Bacillus	54	0.17
27be187284317979c93fd2218ee3722e	38ad2dab3e5e43b7_1	100	627724	629197	Full-length	Staphylococcus_aureus_subsp_aureus_ATCC_BAA_1556	True		d__Bacteria;p__Firmicutes;c__Bacilli;o__Staphylococcales;f__Staphylococcaceae;g__Staphylococcus	467	1.50
b80a1bd6b19379171cafb03a4ddb26a3	266a835027f7464b_2	100	38523	37135	Full-length	Rhodobacter_sphaeroides_ATCC_17029	True	d__Bacteria;p__Proteobacteria;c__Alphaproteobacteria;o__Rhodobacterales;f__Rhodobacteraceae;g__Rhodobacter;s__Rhodobacter_sphaeroides	d__Bacteria;p__Proteobacteria;c__Alphaproteobacteria;o__Rhodobacterales;f__Rhodobacteraceae;g__Rhodobacter	488	1.57
3b44c0cd22f7eeac2386b9f2d475930a	1eb3021faaec49c2_1	100	4584392	4582929	Full-length	Escherichia_coli_ATCC_700926	True		d__Bacteria;p__Proteobacteria;c__Gammaproteobacteria;o__Enterobacterales;f__Enterobacteriaceae;g__Escherichia-Shigella	311	1.00
d3eab112b21cbea371f808f940fa4b0c	f5bcb58692924cb7_1	99.796	1722262	1720790	Full-length	Staphylococcus_epidermidis_ATCC_12228	True		d__Bacteria;p__Firmicutes;c__Bacilli;o__Staphylococcales;f__Staphylococcaceae;g__Staphylococcus	231	0.74
37d8a0129a66ddba216a600739d34631	3042186b85304e8d_1	100	5743540	5742107	Full-length	Clostridium_beijerinckii_ATCC_35702	True	d__Bacteria;p__Firmicutes;c__Clostridia;o__Clostridiales;f__Clostridiaceae;g__Clostridium_sensu_stricto_1;s__Clostridium_beijerinckii	d__Bacteria;p__Firmicutes;c__Clostridia;o__Clostridiales;f__Clostridiaceae;g__Clostridium_sensu_stricto_1	1.83E+02	0.59
7809550a0f7794b926ffcebad7542089	3042186b85304e8d_1	99.861	3404822	3403389	Full-length	Clostridium_beijerinckii_ATCC_35702	True	d__Bacteria;p__Firmicutes;c__Clostridia;o__Clostridiales;f__Clostridiaceae;g__Clostridium_sensu_stricto_1;s__Clostridium_beijerinckii	d__Bacteria;p__Firmicutes;c__Clostridia;o__Clostridiales;f__Clostridiaceae;g__Clostridium_sensu_stricto_1	222	0.71

070e52443d30c5d9e99c8cb06412db1a	3042186b85304e8d_1	99.93	5939487	5938054	Full-length	Clostridium_beijerinckii_ATCC_35702	True	d__Bacteria;p__Firmicutes;c__Clostridia;o__Clostridiales;f__Clostridiaceae;g__Clostridium_sensu_stricto_1;s__Clostridium_beijerinckii	d__Bacteria;p__Firmicutes;c__Clostridia;o__Clostridiales;f__Clostridiaceae;g__Clostridium_sensu_stricto_1	2.11E+02	0.68
