# Supplementary material for: Amplicon-Based Microbiome Profiling: From Second- to Third-Generation Sequencing for Higher Taxonomic Resolution
Source: Genes (Basel). 2023 Jul 31;14(8):1567. doi: 10.3390/genes14081567 (PMC10454624; doi:10.3390/genes14081567)
Supplement: Supplementary file 1 [file genes-14-01567-s001.zip › Supplementary Table S6.pdf]

Supplementary Table S6. Differential abundance analysis of the microbiome, using ANCOM-BC. Statistically significant differences are considered for log fold change (lfc) > |1| and adjusted p-value (q_val) < 0.05. "Comparison" shows the sequencing approaches of the pairwise comparison for each assigned taxon.

Comparison	Rank level	Assigned taxa	lfc	q_val
V3V4 vs Full-length	Phylum	Firmicutes	-0.36	0.00882802
V3V4 vs Full-length	Phylum	Patescibacteria	-0.10	0.81988243
V3V4 vs Full-length	Phylum	Bacteroidota	0.00	0.98865745
V3V4 vs Full-length	Phylum	Proteobacteria	-0.02	0.98865745
V3V4 vs Full-length	Phylum	Verrucomicrobiota	-0.83	0.00437336
V3V4 vs Full-length	Phylum	Actinobacteriota	0.30	0.31606409
V3V4 vs Full-length	Phylum	Cyanobacteria	-0.37	0.06081124
V3V4 vs Full-length	Phylum	Deferribacterota	-0.33	0.334398
V3V4 vs Full-length	Phylum	Desulfobacterota	0.64	0.00550924
V3V4 vs Full-length	Class	Clostridia	0.15	0.71635862
V3V4 vs Full-length	Class	Bacilli	-0.76	0.00586132
V3V4 vs Full-length	Class	Saccharimonadia	-0.09	0.84624623
V3V4 vs Full-length	Class	Bacteroidia	0.00	0.97562163
V3V4 vs Full-length	Class	Alphaproteobacteria	-0.15	0.79416237
V3V4 vs Full-length	Class	Gammaproteobacteria	0.02	0.97562163
V3V4 vs Full-length	Class	Verrucomicrobiae	-0.83	0.01143652
V3V4 vs Full-length	Class	Actinobacteria	-0.19	0.79416237
V3V4 vs Full-length	Class	Coriobacteriia	1.62	6.06E-12
V3V4 vs Full-length	Class	Vampirivibrionia	-0.37	0.19391492
V3V4 vs Full-length	Class	Deferribacteres	-0.33	0.61716614
V3V4 vs Full-length	Class	Desulfovibrionia	0.64	0.03097129
V3V4 vs Full-length	Order	Peptostreptococcales-Tissierellales	0.26	0.19806938
V3V4 vs Full-length	Order	RF39	-0.99	4.10E-05
V3V4 vs Full-length	Order	Lactobacillales	-0.51	0.0674528
V3V4 vs Full-length	Order	Erysipelotrichales	-0.66	0.12157119

V3V4 vs Full-length	Order	Acholeplasmatales	-2.05	1.62E-09
V3V4 vs Full-length	Order	Saccharimonadales	-0.01	0.98980072
V3V4 vs Full-length	Order	Bacteroidales	0.09	0.73706211
V3V4 vs Full-length	Order	Christensenellales	-1.74	0
V3V4 vs Full-length	Order	Clostridia_vadinBB60_group	0.28	0.26669589
V3V4 vs Full-length	Order	Rhodospirillales	-0.07	0.91981644
V3V4 vs Full-length	Order	Clostridiales	-2.33	2.51E-21
V3V4 vs Full-length	Order	Burkholderiales	0.12	0.73706211
V3V4 vs Full-length	Order	Verrucomicrobiales	-0.75	0.02883061
V3V4 vs Full-length	Order	Oscillospirales	0.31	0.12157119
V3V4 vs Full-length	Order	Bifidobacteriales	-0.09	0.91981644
V3V4 vs Full-length	Order	Coriobacteriales	1.70	1.22E-11
V3V4 vs Full-length	Order	Gastranaerophilales	-0.29	0.2804299
V3V4 vs Full-length	Order	Deferribacterales	-0.25	0.64089342
V3V4 vs Full-length	Order	Enterobacterales	-1.46	0.01240503
V3V4 vs Full-length	Order	Desulfovibrionales	0.72	0.00978741
V3V4 vs Full-length	Order	Eubacteriales	-2.01	0
V3V4 vs Full-length	Order	Peptococcales	-0.01	0.98980072
V3V4 vs Full-length	Order	Monoglobales	-0.18	0.76912913
V3V4 vs Full-length	Order	Clostridia_UCG-014	0.00	0.98980072
V3V4 vs Full-length	Order	Lachnospirales	0.28	0.2140446
V3V4 vs Full-length	Family	Anaerovoracaceae	0.13	0.37917324
V3V4 vs Full-length	Family	RF39	-1.12	8.12E-11
V3V4 vs Full-length	Family	Streptococcaceae	0.00	0.99898135
V3V4 vs Full-length	Family	Lactobacillaceae	-0.64	0.00086129
V3V4 vs Full-length	Family	Erysipelatoclostridiaceae	0.11	0.74559684
V3V4 vs Full-length	Family	Erysipelotrichaceae	-0.93	0.02174458
V3V4 vs Full-length	Family	Acholeplasmataceae	-2.18	1.90E-14
V3V4 vs Full-length	Family	Saccharimonadaceae	-0.14	0.63113285

V3V4 vs Full-length	Family	Prevotellaceae	-0.82	0.07533366
V3V4 vs Full-length	Family	Marinifilaceae	-0.06	0.68513029
V3V4 vs Full-length	Family	Rikenellaceae	0.04	0.72627147
V3V4 vs Full-length	Family	Muribaculaceae	-0.03	0.75393095
V3V4 vs Full-length	Family	Bacteroidaceae	-0.05	0.73409521
V3V4 vs Full-length	Family	Christensenellaceae	-1.87	0
V3V4 vs Full-length	Family	Clostridia_vadinBB60_group	0.15	0.50422398
V3V4 vs Full-length	Family	Clostridiaceae	-2.46	2.63E-40
V3V4 vs Full-length	Family	Sutterellaceae	-0.01	0.98068821
V3V4 vs Full-length	Family	Akkermansiaceae	-0.88	0.00068284
V3V4 vs Full-length	Family	Bacteroidales	-1.17	0
V3V4 vs Full-length	Family	UCG-010	-0.29	0.17889018
V3V4 vs Full-length	Family	Bifidobacteriaceae	-0.22	0.63113285
V3V4 vs Full-length	Family	Atopobiaceae	0.91	0.00263392
V3V4 vs Full-length	Family	Eggerthellaceae	2.03	3.63E-40
V3V4 vs Full-length	Family	Gastranaerophilales	-0.42	0.02174458
V3V4 vs Full-length	Family	Deferribacteraceae	-0.38	0.29997227
V3V4 vs Full-length	Family	Enterobacteriaceae	-1.59	0.00167461
V3V4 vs Full-length	Family	Desulfovibrionaceae	0.59	0.00617791
V3V4 vs Full-length	Family	Anaerofustaceae	-2.14	0
V3V4 vs Full-length	Family	Peptococcaceae	-0.14	0.54037102
V3V4 vs Full-length	Family	Butyricicoccaceae	0.82	0.0001502
V3V4 vs Full-length	Family	[Eubacterium]_coprostanoligenes_group	-0.73	0.02473347
V3V4 vs Full-length	Family	Ruminococcaceae	0.10	0.54037102
V3V4 vs Full-length	Family	Oscillospiraceae	0.42	0.02509794
V3V4 vs Full-length	Family	Monoglobaceae	-0.31	0.50422398
V3V4 vs Full-length	Family	Clostridia_UCG-014	-0.13	0.58865687
V3V4 vs Full-length	Family	Lachnospiraceae	0.15	0.44296687
V3V4 vs Full-length	Family	Defluviitaleaceae	0.01	0.98068821

V3V4 vs Full-length	Family	Tannerellaceae	-3.84	0
V3V4 vs Full-length	Genus	Anaerovorax	-1.16	6.31E-08
V3V4 vs Full-length	Genus	Family_XIII_AD3011_group	-1.11	3.93E-05
V3V4 vs Full-length	Genus	[Eubacterium]_nodatum_group	0.04	0.90766349
V3V4 vs Full-length	Genus	[Eubacterium]_brachy_group	-0.20	0.44615202
V3V4 vs Full-length	Genus	Family_XIII_UCG-001	-0.33	0.22959991
V3V4 vs Full-length	Genus	RF39	-1.11	1.99E-09
V3V4 vs Full-length	Genus	Streptococcus	0.02	0.97374242
V3V4 vs Full-length	Genus	Lactobacillus	-0.62	0.0028288
V3V4 vs Full-length	Genus	Erysipelatoclostridium	-0.20	0.62946504
V3V4 vs Full-length	Genus	Faecalibaculum	-1.18	0.0159903
V3V4 vs Full-length	Genus	Anaeroplasma	-2.16	1.47E-14
V3V4 vs Full-length	Genus	Prevotellaceae_UCG-001	-0.80	0.09244068
V3V4 vs Full-length	Genus	Odoribacter	-0.05	0.80986787
V3V4 vs Full-length	Genus	Rikenellaceae_RC9_gut_group	-0.36	0.25679689
V3V4 vs Full-length	Genus	Alistipes	0.08	0.43474886
V3V4 vs Full-length	Genus	Muribaculaceae	-0.02	0.91796392
V3V4 vs Full-length	Genus	Muribaculum	0.19	0.27499928
V3V4 vs Full-length	Genus	Bacteroides	-0.03	0.91796392
V3V4 vs Full-length	Genus	Clostridia_vadinBB60_group	0.16	0.34822098
V3V4 vs Full-length	Genus	Parasutterella	0.01	0.97374242
V3V4 vs Full-length	Genus	Akkermansia	-0.86	0.00176479
V3V4 vs Full-length	Genus	Bacteroidales	-1.15	0
V3V4 vs Full-length	Genus	UCG-010	-0.27	0.24593715
V3V4 vs Full-length	Genus	Bifidobacterium	-0.20	0.68401918
V3V4 vs Full-length	Genus	Coriobacteriaceae_UCG-002	2.48	0
V3V4 vs Full-length	Genus	Enterorhabdus	2.04	3.75E-39
V3V4 vs Full-length	Genus	Gastranaerophilales	-0.40	0.02240791
V3V4 vs Full-length	Genus	Mucispirillum	-0.36	0.24593715

V3V4 vs Full-length	Genus	Salmonella	-1.59	0.00123087
V3V4 vs Full-length	Genus	Bilophila	0.61	0.00098695
V3V4 vs Full-length	Genus	Anaerofustis	-2.12	0
V3V4 vs Full-length	Genus	Peptococcus	-0.66	0.00907555
V3V4 vs Full-length	Genus	UCG-009	0.65	0.0006191
V3V4 vs Full-length	Genus	Butyricicoccus	0.76	0.00201117
V3V4 vs Full-length	Genus	[Eubacterium]_coprostanoligenes_group	-0.71	0.03625896
V3V4 vs Full-length	Genus	Harryflintia	-1.28	1.06E-07
V3V4 vs Full-length	Genus	Negativibacillus	-2.41	2.45E-30
V3V4 vs Full-length	Genus	Paludicola	-1.67	3.21E-16
V3V4 vs Full-length	Genus	Anaerotruncus	0.54	0.02482389
V3V4 vs Full-length	Genus	[Eubacterium]_siraeum_group	-0.79	0.02888684
V3V4 vs Full-length	Genus	Ruminococcaceae	-1.99	1.28E-24
V3V4 vs Full-length	Genus	Ruminococcus	0.01	0.97374242
V3V4 vs Full-length	Genus	UCG-005	-1.70	6.71E-10
V3V4 vs Full-length	Genus	Oscillibacter	-0.12	0.72195842
V3V4 vs Full-length	Genus	NK4A214_group	-0.47	0.07105933
V3V4 vs Full-length	Genus	Colidextribacter	1.02	2.83E-10
V3V4 vs Full-length	Genus	Intestinimonas	0.02	0
V3V4 vs Full-length	Genus	Monoglobus	-0.29	0.46235088
V3V4 vs Full-length	Genus	Clostridia_UCG-014	-0.11	0.66628968
V3V4 vs Full-length	Genus	Tyzzerella	-0.94	6.87E-05
V3V4 vs Full-length	Genus	Defluviitaleaceae_UCG-011	0.03	0.95483939
V3V4 vs Full-length	Genus	Lachnospiraceae_UCG-010	-1.95	8.28E-26
V3V4 vs Full-length	Genus	ASF356	0.32	0.18922993
V3V4 vs Full-length	Genus	Tuzzerella	0.37	0.15592945
V3V4 vs Full-length	Genus	GCA-900066575	1.06	2.51E-08
V3V4 vs Full-length	Genus	Lachnospiraceae	0.97	1.84E-13
V3V4 vs Full-length	Genus	Lachnospiraceae_FCS020_group	0.91	0.00172751

V3V4 vs Full-length	Genus	[Eubacterium]_xylanophilum_group	0.15	0.51344532
V3V4 vs Full-length	Genus	Lachnospiraceae_NK4A136_group	-0.08	0.62946504
V3V4 vs Full-length	Genus	[Eubacterium]_fissicatena_group	-0.23	0.52953284
V3V4 vs Full-length	Genus	Acetatifactor	-2.07	5.09E-24
V3V4 vs Full-length	Genus	Blautia	0.82	0.00196538
V3V4 vs Full-length	Genus	Marvinbryantia	-0.86	0.04295581
V3V4 vs Full-length	Genus	Lachnoclostridium	0.40	0.04351217
V3V4 vs Full-length	Genus	Lachnospiraceae_UCG-006	-0.01	0.97648681
V3V4 vs Full-length	Genus	Roseburia	0.33	0.24593715
V3V4 vs Full-length	Genus	A2	-0.29	0.49267992
V3V4 vs Full-length	Genus	Lachnospiraceae_UCG-001	0.05	0.95483939
V3V4 vs Full-length	Genus	Olsenella	-4.21	0
V3V4 vs Full-length	Genus	Parabacteroides	-3.82	0
V3V4 vs Full-length	Species	Streptococcus_danieliae	0.80	0.00040229
V3V4 vs Full-length	Species	Lactobacillus_murinus	0.77	7.46E-05
V3V4 vs Full-length	Species	Lactobacillus_reuteri	0.35	0.30456009
V3V4 vs Full-length	Species	Bacteroides_acidifaciens	1.37	3.02E-16
V3V4 vs Full-length	Species	Bacteroides_caecimuris	0.01	0.98168479
V3V4 vs Full-length	Species	Bifidobacterium_choerinum	5.37	0
V3V4 vs Full-length	Species	Adlercreutzia_mucosicola	1.34	9.62E-06
V3V4 vs Full-length	Species	Akkermansia_muciniphila	-0.08	0.78618179
V3V4 vs Full-length	Species	[Clostridium]_leptum	1.30	2.65E-13
V3V4 vs Full-length	Species	Eubacterium_plexicaudatum	-2.41	0
V3V4 vs Full-length	Species	Muribaculum_intestinale	-5.41	0
V3V4 vs Full-length	Species	Mucispirillum_schaedleri	-4.53	0
V3V4 vs Full-length	Species	Lactobacillus_johnsonii	-8.22	0
V3V4 vs Full-length	Species	Bifidobacterium_pseudolongum	-6.68	0
V3V4 vs Full-length	Species	[Clostridium]_leptum_3	-3.87	0
V3V4 vs Full-length	Species	Parabacteroides_goldsteinii	-3.04	0

V3V4 vs Multiplex	Phylum	Firmicutes	0.00	0.98
V3V4 vs Multiplex	Phylum	Patescibacteria	2.34	0.00
V3V4 vs Multiplex	Phylum	Bacteroidota	0.78	0.00
V3V4 vs Multiplex	Phylum	Proteobacteria	-1.25	0.00
V3V4 vs Multiplex	Phylum	Verrucomicrobiota	1.07	0.00
V3V4 vs Multiplex	Phylum	Actinobacteriota	-0.60	0.01
V3V4 vs Multiplex	Phylum	Cyanobacteria	4.49	0.00
V3V4 vs Multiplex	Phylum	Deferribacterota	0.05	0.98
V3V4 vs Multiplex	Phylum	Desulfobacterota	4.76	0.00
V3V4 vs Multiplex	Class	Clostridia	-0.32	0.08423645
V3V4 vs Multiplex	Class	Bacilli	0.58	0.01881771
V3V4 vs Multiplex	Class	Saccharimonadia	2.15	0
V3V4 vs Multiplex	Class	Bacteroidia	0.59	9.81E-06
V3V4 vs Multiplex	Class	Alphaproteobacteria	-3.22	4.98E-49
V3V4 vs Multiplex	Class	Gammaproteobacteria	0.03	0.87328174
V3V4 vs Multiplex	Class	Verrucomicrobiae	0.88	0.00324566
V3V4 vs Multiplex	Class	Actinobacteria	-0.73	0.0678394
V3V4 vs Multiplex	Class	Coriobacteriia	-1.12	1.31E-11
V3V4 vs Multiplex	Class	Vampirivibrionia	4.30	0
V3V4 vs Multiplex	Class	Deferribacteres	-0.15	0.69713075
V3V4 vs Multiplex	Class	Desulfovibrionia	4.57	0
V3V4 vs Multiplex	Order	Peptostreptococcales-Tissierellales	-4.99	3.40E-249
V3V4 vs Multiplex	Order	RF39	2.97	6.90E-42
V3V4 vs Multiplex	Order	Staphylococcales	-1.03	1.59E-05
V3V4 vs Multiplex	Order	Lactobacillales	4.85	2.23E-85
V3V4 vs Multiplex	Order	Erysipelotrichales	4.18	2.47E-51
V3V4 vs Multiplex	Order	Acholeplasmatales	0.37	0
V3V4 vs Multiplex	Order	Saccharimonadales	2.02	0
V3V4 vs Multiplex	Order	Bacteroidales	0.62	2.48E-06

V3V4 vs Multiplex	Order	Christensenellales	0.25	0
V3V4 vs Multiplex	Order	Clostridia_vadinBB60_group	5.10	0
V3V4 vs Multiplex	Order	Rhodospirillales	0.98	0.00011844
V3V4 vs Multiplex	Order	Burkholderiales	-0.03	0.88563593
V3V4 vs Multiplex	Order	Verrucomicrobiales	0.76	0.01290805
V3V4 vs Multiplex	Order	Oscillospirales	2.15	1.54E-28
V3V4 vs Multiplex	Order	Propionibacteriales	-0.81	0
V3V4 vs Multiplex	Order	Bifidobacteriales	-0.84	0.04490772
V3V4 vs Multiplex	Order	Coriobacteriales	-1.24	2.70E-12
V3V4 vs Multiplex	Order	Gastranaerophilales	4.18	0
V3V4 vs Multiplex	Order	Deferribacterales	-0.27	0.41752508
V3V4 vs Multiplex	Order	Enterobacterales	-1.05	0.07619272
V3V4 vs Multiplex	Order	Desulfovibrionales	4.45	0
V3V4 vs Multiplex	Order	Eubacteriales	-0.03	0
V3V4 vs Multiplex	Order	Peptococcales	4.33	0
V3V4 vs Multiplex	Order	Monoglobales	3.56	0
V3V4 vs Multiplex	Order	Clostridia_UCG-014	7.09	0
V3V4 vs Multiplex	Order	Lachnospirales	6.20	9.82E-240
V3V4 vs Multiplex	Order	Bacillales	-9.09	0
V3V4 vs Multiplex	Order	Sphingomonadales	-4.50	0
V3V4 vs Multiplex	Order	Caulobacterales	-9.10	0
V3V4 vs Multiplex	Family	Anaerovoracaceae	4.70	0
V3V4 vs Multiplex	Family	RF39	2.53	5.36E-16
V3V4 vs Multiplex	Family	Staphylococcaceae	-1.48	8.21E-06
V3V4 vs Multiplex	Family	Streptococcaceae	2.02	3.83E-10
V3V4 vs Multiplex	Family	Lactobacillaceae	4.41	5.90E-38
V3V4 vs Multiplex	Family	Erysipelatoclostridiaceae	4.50	0
V3V4 vs Multiplex	Family	Erysipelotrichaceae	3.02	8.90E-14
V3V4 vs Multiplex	Family	Acholeplasmataceae	-0.08	0

V3V4 vs Multiplex	Family	Saccharimonadaceae	1.58	0
V3V4 vs Multiplex	Family	Prevotellaceae	3.48	2.76E-14
V3V4 vs Multiplex	Family	Marinifilaceae	0.62	0.11272383
V3V4 vs Multiplex	Family	Rikenellaceae	0.01	0.96502105
V3V4 vs Multiplex	Family	Muribaculaceae	0.65	0.00750378
V3V4 vs Multiplex	Family	Bacteroidaceae	-0.38	0.12258149
V3V4 vs Multiplex	Family	Christensenellaceae	-0.19	0
V3V4 vs Multiplex	Family	Clostridia_vadinBB60_group	4.65	0
V3V4 vs Multiplex	Family	uncultured	0.54	0.12258149
V3V4 vs Multiplex	Family	Sutterellaceae	4.34	3.71E-53
V3V4 vs Multiplex	Family	Akkermansiaceae	0.31	0.44425701
V3V4 vs Multiplex	Family	UCG-010	3.72	0
V3V4 vs Multiplex	Family	Propionibacteriaceae	-1.26	0
V3V4 vs Multiplex	Family	Bifidobacteriaceae	-1.28	0.01200406
V3V4 vs Multiplex	Family	Atopobiaceae	-2.39	8.52E-10
V3V4 vs Multiplex	Family	Eggerthellaceae	-0.84	0.00165684
V3V4 vs Multiplex	Family	Gastranaerophilales	3.73	0
V3V4 vs Multiplex	Family	Deferribacteraceae	-0.71	0.09857396
V3V4 vs Multiplex	Family	Enterobacteriaceae	1.42	0.01117172
V3V4 vs Multiplex	Family	Desulfovibrionaceae	4.00	0
V3V4 vs Multiplex	Family	Anaerofustaceae	-0.47	0
V3V4 vs Multiplex	Family	Peptococcaceae	3.89	0
V3V4 vs Multiplex	Family	Butyricicoccaceae	4.83	1.33E-73
V3V4 vs Multiplex	Family	[Eubacterium]_coprostanoligenes_group	2.64	0
V3V4 vs Multiplex	Family	Ruminococcaceae	3.59	6.76E-31
V3V4 vs Multiplex	Family	Oscillospiraceae	0.53	0.09990978
V3V4 vs Multiplex	Family	Monoglobaceae	3.11	0
V3V4 vs Multiplex	Family	Clostridia_UCG-014	6.65	0
V3V4 vs Multiplex	Family	Lachnospiraceae	5.75	4.32E-92

V3V4 vs Multiplex	Family	Defluviitaleaceae	3.48	0
V3V4 vs Multiplex	Family	Peptostreptococcaceae	-11.17	0
V3V4 vs Multiplex	Family	Bacillaceae	-9.54	0
V3V4 vs Multiplex	Family	Tannerellaceae	-4.28	0
V3V4 vs Multiplex	Family	Sphingomonadaceae	-4.95	0
V3V4 vs Multiplex	Family	Alcaligenaceae	-8.21	0
V3V4 vs Multiplex	Family	Yersiniaceae	-4.20	0
V3V4 vs Multiplex	Family	Barnesiellaceae	-1.56	0
V3V4 vs Multiplex	Family	Parvularculaceae	-9.55	0
V3V4 vs Multiplex	Genus	Anaerovorax	0.14	0
V3V4 vs Multiplex	Genus	Family_XIII_AD3011_group	0.41	0
V3V4 vs Multiplex	Genus	[Eubacterium]_nodatum_group	3.33	0
V3V4 vs Multiplex	Genus	[Eubacterium]_brachy_group	1.86	0
V3V4 vs Multiplex	Genus	Family_XIII_UCG-001	1.00	0
V3V4 vs Multiplex	Genus	RF39	1.80	4.84E-09
V3V4 vs Multiplex	Genus	Staphylococcus	-2.20	6.41E-12
V3V4 vs Multiplex	Genus	Streptococcus	1.29	5.42E-05
V3V4 vs Multiplex	Genus	Lactobacillus	3.68	7.87E-27
V3V4 vs Multiplex	Genus	Erysipelatoclostridium	2.31	0
V3V4 vs Multiplex	Genus	Faecalibaculum	4.52	0
V3V4 vs Multiplex	Genus	Anaeroplasma	-0.80	0
V3V4 vs Multiplex	Genus	Prevotellaceae_UCG-001	3.49	0
V3V4 vs Multiplex	Genus	Odoribacter	-0.10	0.79513448
V3V4 vs Multiplex	Genus	Rikenellaceae_RC9_gut_group	-1.36	0.00051598
V3V4 vs Multiplex	Genus	Alistipes	-0.66	0.00280222
V3V4 vs Multiplex	Genus	Muribaculaceae	7.71	6.25E-185
V3V4 vs Multiplex	Genus	Muribaculum	-3.24	5.58E-36
V3V4 vs Multiplex	Genus	Bacteroides	-1.10	7.19E-06
V3V4 vs Multiplex	Genus	Clostridia_vadinBB60_group	3.93	0

V3V4 vs Multiplex	Genus	Parasutterella	3.61	4.23E-36
V3V4 vs Multiplex	Genus	Akkermansia	-0.41	0.30609411
V3V4 vs Multiplex	Genus	UCG-010	3.00	0
V3V4 vs Multiplex	Genus	Bifidobacterium	-2.00	7.03E-05
V3V4 vs Multiplex	Genus	Coriobacteriaceae_UCG-002	3.37	0
V3V4 vs Multiplex	Genus	Enterorhabdus	-0.96	0.00015616
V3V4 vs Multiplex	Genus	Gastranaerophilales	3.01	0
V3V4 vs Multiplex	Genus	Mucispirillum	-1.44	0.0003291
V3V4 vs Multiplex	Genus	Salmonella	0.93	0
V3V4 vs Multiplex	Genus	Bilophila	3.28	0
V3V4 vs Multiplex	Genus	Anaerofustis	-1.20	0
V3V4 vs Multiplex	Genus	Peptococcus	0.87	0
V3V4 vs Multiplex	Genus	UCG-009	3.51	0
V3V4 vs Multiplex	Genus	Butyricicoccus	2.99	0
V3V4 vs Multiplex	Genus	[Eubacterium]_coprostanoligenes_group	1.91	0
V3V4 vs Multiplex	Genus	Harryflintia	-0.03	0
V3V4 vs Multiplex	Genus	Paludicola	-0.48	0
V3V4 vs Multiplex	Genus	Anaerotruncus	3.96	2.91E-48
V3V4 vs Multiplex	Genus	[Eubacterium]_siraeum_group	1.14	0
V3V4 vs Multiplex	Genus	Ruminococcaceae	-0.95	0
V3V4 vs Multiplex	Genus	Ruminococcus	5.44	4.50E-95
V3V4 vs Multiplex	Genus	UCG-005	-0.12	0
V3V4 vs Multiplex	Genus	Oscillibacter	-1.49	0.00010857
V3V4 vs Multiplex	Genus	NK4A214_group	0.81	0
V3V4 vs Multiplex	Genus	Colidextribacter	3.12	2.72E-26
V3V4 vs Multiplex	Genus	Intestinimonas	0.91	0
V3V4 vs Multiplex	Genus	Monoglobus	2.39	0
V3V4 vs Multiplex	Genus	Clostridia_UCG-014	5.92	0
V3V4 vs Multiplex	Genus	Tyzzerella	0.12	0

V3V4 vs Multiplex	Genus	Defluviitaleaceae_UCG-011	2.75	0
V3V4 vs Multiplex	Genus	Lachnospiraceae_UCG-010	-0.96	0
V3V4 vs Multiplex	Genus	ASF356	3.83	0
V3V4 vs Multiplex	Genus	Tuzzerella	2.40	0
V3V4 vs Multiplex	Genus	GCA-900066575	3.98	0
V3V4 vs Multiplex	Genus	Lachnospiraceae_FCS020_group	2.30	0
V3V4 vs Multiplex	Genus	[Eubacterium]_xylanophilum_group	4.30	0
V3V4 vs Multiplex	Genus	Lachnospiraceae_NK4A136_group	6.79	0
V3V4 vs Multiplex	Genus	[Eubacterium]_fissicatena_group	4.38	0
V3V4 vs Multiplex	Genus	Acetatifactor	-2.47	5.44E-11
V3V4 vs Multiplex	Genus	Blautia	3.09	7.84E-19
V3V4 vs Multiplex	Genus	Marvinbryantia	1.08	0
V3V4 vs Multiplex	Genus	Lachnoclostridium	3.80	2.08E-39
V3V4 vs Multiplex	Genus	Lachnospiraceae_UCG-006	4.58	0
V3V4 vs Multiplex	Genus	Roseburia	4.09	4.62E-39
V3V4 vs Multiplex	Genus	A2	1.16	0
V3V4 vs Multiplex	Genus	Lachnospiraceae_UCG-001	4.71	0
V3V4 vs Multiplex	Genus	Tepidibacter	-11.90	0
V3V4 vs Multiplex	Genus	Bacillus	-10.26	0
V3V4 vs Multiplex	Genus	Zymomonas	-5.67	0
V3V4 vs Multiplex	Genus	Pigmentiphaga	-8.93	0
V3V4 vs Multiplex	Genus	Serratia	-4.92	0
V3V4 vs Multiplex	Genus	Libanicoccus	-4.86	0
V3V4 vs Multiplex	Genus	Barnesiella	-2.28	0
V3V4 vs Multiplex	Genus	Amphiplicatus	-10.27	0
V3V4 vs Multiplex	Genus	UCG-003	-2.27	0
V3V4 vs Multiplex	Genus	Olsenella	-2.75	0
V3V4 vs Multiplex	Species	Streptococcus_danieliae	1.10	0
V3V4 vs Multiplex	Species	Lactobacillus_murinus	5.26	0

V3V4 vs Multiplex	Species	Lactobacillus_reuteri	4.83	0
V3V4 vs Multiplex	Species	Bacteroides_acidifaciens	5.68	0
V3V4 vs Multiplex	Species	Bacteroides_caecimuris	2.44	0
V3V4 vs Multiplex	Species	Bifidobacterium_choerinum	5.17	0
V3V4 vs Multiplex	Species	Adlercreutzia_mucosicola	1.14	0.0005554
V3V4 vs Multiplex	Species	Akkermansia_muciniphila	6.06	0
V3V4 vs Multiplex	Species	[Clostridium]_leptum	1.80	5.04E-13
V3V4 vs Multiplex	Species	Alistipes_finegoldii	-5.13	0
V3V4 vs Multiplex	Species	Lactobacillus_vaginalis	-10.58	0
V3V4 vs Multiplex	Species	Muribaculum_intestinale	-11.31	0
V3V4 vs Multiplex	Species	Mucispirillum_schaedleri	-6.70	0
V3V4 vs Multiplex	Species	Zymomonas_mobilis	-5.99	0
V3V4 vs Multiplex	Species	Alistipes_putredinis	-2.94	0
V3V4 vs Multiplex	Species	[Clostridium]_leptum_1	-6.43	0
V3V4 vs Multiplex	Species	Adlercreutzia_muris	-2.63	0
V3V4 vs Multiplex	Species	Staphylococcus_carnosus	-2.72	0
V3V4 vs Multiplex	Species	Acetatifactor_muris	-3.50	0
V3V4 vs Multiplex	Species	Parasutterella_excrementihominis	-3.23	0
V3V4 vs Multiplex	Species	Alistipes_onderdonkii	-2.62	0
Full-length vs Multiplex	Phylum	Firmicutes	0.00	0.99
Full-length vs Multiplex	Phylum	Bacteroidota	0.42	0.00
Full-length vs Multiplex	Phylum	Kingdom:d__Bacteria	-7.24	0.00
Full-length vs Multiplex	Phylum	Proteobacteria	-1.59	0.00
Full-length vs Multiplex	Phylum	Verrucomicrobiota	1.54	0.00
Full-length vs Multiplex	Phylum	Actinobacteriota	-1.26	0.00
Full-length vs Multiplex	Phylum	Cyanobacteria	4.51	0.00
Full-length vs Multiplex	Phylum	Deferribacterota	0.02	0.99
Full-length vs Multiplex	Phylum	Desulfobacterota	3.77	0.00
Full-length vs Multiplex	Class	Clostridia	-0.47	0.00381139

Full-length vs Multiplex	Class	Bacilli	1.34	6.31E-09
Full-length vs Multiplex	Class	Bacteroidia	0.59	6.31E-09
Full-length vs Multiplex	Class	Alphaproteobacteria	-3.07	2.28E-56
Full-length vs Multiplex	Class	Gammaproteobacteria	0.01	0.96660893
Full-length vs Multiplex	Class	Verrucomicrobiae	1.70	1.83E-12
Full-length vs Multiplex	Class	Actinobacteria	-0.54	0.09744696
Full-length vs Multiplex	Class	Coriobacteriia	-2.74	1.87E-41
Full-length vs Multiplex	Class	Vampirivibrionia	4.67	0
Full-length vs Multiplex	Class	Deferribacteres	0.18	0.58096081
Full-length vs Multiplex	Class	Desulfovibrionia	3.93	0
Full-length vs Multiplex	Order	Peptostreptococcales-Tissierellales	-5.79	1.87E-127
Full-length vs Multiplex	Order	RF39	3.43	1.52E-33
Full-length vs Multiplex	Order	Staphylococcales	0.86	0
Full-length vs Multiplex	Order	Lactobacillales	4.82	6.19E-49
Full-length vs Multiplex	Order	Erysipelotrichales	4.30	3.14E-29
Full-length vs Multiplex	Order	Bacteroidales	0.00	0.99462661
Full-length vs Multiplex	Order	Clostridia_vadinBB60_group	4.29	0
Full-length vs Multiplex	Order	Rhodospirillales	0.52	0.11918383
Full-length vs Multiplex	Order	Burkholderiales	-0.68	0.00680327
Full-length vs Multiplex	Order	Verrucomicrobiales	0.97	0.00529399
Full-length vs Multiplex	Order	Oscillospirales	1.31	1.33E-06
Full-length vs Multiplex	Order	Propionibacteriales	1.15	0
Full-length vs Multiplex	Order	Bifidobacteriales	-1.28	0.00401844
Full-length vs Multiplex	Order	Coriobacteriales	-3.47	2.02E-28
Full-length vs Multiplex	Order	Gastranaerophilales	3.93	0
Full-length vs Multiplex	Order	Deferribacterales	-0.56	0.14895724
Full-length vs Multiplex	Order	Enterobacterales	-0.12	0.84676484
Full-length vs Multiplex	Order	Desulfovibrionales	3.19	0
Full-length vs Multiplex	Order	Peptococcales	3.80	0

Full-length vs Multiplex	Order	Monoglobales	3.20	0
Full-length vs Multiplex	Order	Clostridia_UCG-014	6.56	0
Full-length vs Multiplex	Order	Lachnospirales	5.39	5.02E-89
Full-length vs Multiplex	Order	Bacillales	-7.09	0
Full-length vs Multiplex	Order	Sphingomonadales	-2.50	0
Full-length vs Multiplex	Order	Caulobacterales	-7.10	0
Full-length vs Multiplex	Family	Anaerovoracaceae	3.50	0
Full-length vs Multiplex	Family	RF39	2.58	3.66E-21
Full-length vs Multiplex	Family	Staphylococcaceae	0.00	0
Full-length vs Multiplex	Family	Streptococcaceae	0.94	0.00012765
Full-length vs Multiplex	Family	Lactobacillaceae	3.98	7.88E-36
Full-length vs Multiplex	Family	Erysipelatoclostridiaceae	3.32	0
Full-length vs Multiplex	Family	Erysipelotrichaceae	2.88	7.14E-12
Full-length vs Multiplex	Family	Prevotellaceae	3.22	1.67E-15
Full-length vs Multiplex	Family	Marinifilaceae	-0.39	0.2775509
Full-length vs Multiplex	Family	Rikenellaceae	-1.10	1.60E-08
Full-length vs Multiplex	Family	Muribaculaceae	-0.39	0.06114152
Full-length vs Multiplex	Family	Bacteroidaceae	-1.41	3.14E-12
Full-length vs Multiplex	Family	Clostridia_vadinBB60_group	3.44	0
Full-length vs Multiplex	Family	Sutterellaceae	3.27	4.10E-38
Full-length vs Multiplex	Family	Akkermansiaceae	0.11	0.77213393
Full-length vs Multiplex	Family	UCG-010	2.94	0
Full-length vs Multiplex	Family	Propionibacteriaceae	0.29	0
Full-length vs Multiplex	Family	Bifidobacteriaceae	-2.13	7.61E-07
Full-length vs Multiplex	Family	Atopobiaceae	-4.37	6.40E-36
Full-length vs Multiplex	Family	Eggerthellaceae	-3.94	2.87E-54
Full-length vs Multiplex	Family	Gastranaerophilales	3.08	0
Full-length vs Multiplex	Family	Deferribacteraceae	-1.41	0.0001473
Full-length vs Multiplex	Family	Enterobacteriaceae	1.93	3.24E-07

Full-length vs Multiplex	Family	Desulfovibrionaceae	2.34	0
Full-length vs Multiplex	Family	Peptococcaceae	2.95	0
Full-length vs Multiplex	Family	Butyricicoccaceae	2.93	1.19E-24
Full-length vs Multiplex	Family	[Eubacterium]_coprostanoligenes_group	2.29	0
Full-length vs Multiplex	Family	Ruminococcaceae	2.42	3.08E-18
Full-length vs Multiplex	Family	Oscillospiraceae	-0.97	0.00102157
Full-length vs Multiplex	Family	Monoglobaceae	2.34	0
Full-length vs Multiplex	Family	Clostridia_UCG-014	5.71	0
Full-length vs Multiplex	Family	Lachnospiraceae	4.53	6.76E-70
Full-length vs Multiplex	Family	Defluviitaleaceae	2.39	0
Full-length vs Multiplex	Family	Peptostreptococcaceae	-9.58	0
Full-length vs Multiplex	Family	Bacillaceae	-7.94	0
Full-length vs Multiplex	Family	Tannerellaceae	-1.52	1.43E-06
Full-length vs Multiplex	Family	Sphingomonadaceae	-3.35	0
Full-length vs Multiplex	Family	Alcaligenaceae	-6.61	0
Full-length vs Multiplex	Family	Yersiniaceae	-2.61	0
Full-length vs Multiplex	Family	Barnesiellaceae	0.04	0
Full-length vs Multiplex	Family	Parvularculaceae	-7.95	0
Full-length vs Multiplex	Genus	Anaerovorax	0.00	0
Full-length vs Multiplex	Genus	Family_XIII_AD3011_group	0.21	0
Full-length vs Multiplex	Genus	[Eubacterium]_nodatum_group	1.98	0
Full-length vs Multiplex	Genus	[Eubacterium]_brachy_group	0.76	0
Full-length vs Multiplex	Genus	Family_XIII_UCG-001	0.02	0
Full-length vs Multiplex	Genus	RF39	1.60	5.69E-16
Full-length vs Multiplex	Genus	Staphylococcus	-0.97	0
Full-length vs Multiplex	Genus	Streptococcus	-0.03	0.8788774
Full-length vs Multiplex	Genus	Lactobacillus	3.00	4.71E-34
Full-length vs Multiplex	Genus	Erysipelatoclostridium	1.20	0
Full-length vs Multiplex	Genus	Faecalibaculum	4.39	0

Full-length vs Multiplex	Genus	Prevotellaceae_UCG-001	2.98	0
Full-length vs Multiplex	Genus	Odoribacter	-1.36	2.16E-07
Full-length vs Multiplex	Genus	Rikenellaceae_RC9_gut_group	-2.31	1.32E-19
Full-length vs Multiplex	Genus	Alistipes	-2.05	3.91E-76
Full-length vs Multiplex	Genus	Muribaculaceae	6.43	0
Full-length vs Multiplex	Genus	Muribaculum	-4.73	6.10E-234
Full-length vs Multiplex	Genus	Bacteroides	-2.38	1.01E-79
Full-length vs Multiplex	Genus	Clostridia_vadinBB60_group	2.46	0
Full-length vs Multiplex	Genus	Parasutterella	2.30	1.21E-35
Full-length vs Multiplex	Genus	Akkermansia	-0.86	0.00099096
Full-length vs Multiplex	Genus	UCG-010	1.97	0
Full-length vs Multiplex	Genus	Cutibacterium	-0.68	0
Full-length vs Multiplex	Genus	Bifidobacterium	-3.11	2.29E-18
Full-length vs Multiplex	Genus	Enterorhabdus	-4.30	9.04E-134
Full-length vs Multiplex	Genus	Gastranaerophilales	2.11	0
Full-length vs Multiplex	Genus	Mucispirillum	-2.38	5.67E-17
Full-length vs Multiplex	Genus	Salmonella	1.22	0
Full-length vs Multiplex	Genus	Bilophila	1.37	0
Full-length vs Multiplex	Genus	Peptococcus	0.22	0
Full-length vs Multiplex	Genus	uncultured_3	1.89	0
Full-length vs Multiplex	Genus	UCG-009	1.55	0
Full-length vs Multiplex	Genus	Butyricicoccus	0.92	0
Full-length vs Multiplex	Genus	[Eubacterium]_coprostanoligenes_group	1.32	0
Full-length vs Multiplex	Genus	Harryflintia	-0.05	0
Full-length vs Multiplex	Genus	Paludicola	-0.12	0
Full-length vs Multiplex	Genus	Anaerotruncus	2.12	8.00E-22
Full-length vs Multiplex	Genus	[Eubacterium]_siraeum_group	0.63	0
Full-length vs Multiplex	Genus	Ruminococcus	4.12	2.05E-155
Full-length vs Multiplex	Genus	UCG-005	0.27	0

Full-length vs Multiplex	Genus	Oscillibacter	-2.68	2.14E-28
Full-length vs Multiplex	Genus	NK4A214_group	-0.02	0
Full-length vs Multiplex	Genus	Colidextribacter	0.79	0.00048695
Full-length vs Multiplex	Genus	Monoglobus	1.37	0
Full-length vs Multiplex	Genus	Clostridia_UCG-014	4.73	0
Full-length vs Multiplex	Genus	Defluviitaleaceae_UCG-011	1.42	0
Full-length vs Multiplex	Genus	ASF356	2.21	0
Full-length vs Multiplex	Genus	Tuzzerella	0.72	0
Full-length vs Multiplex	Genus	GCA-900066575	1.61	0
Full-length vs Multiplex	Genus	Lachnospiraceae_FCS020_group	0.08	0
Full-length vs Multiplex	Genus	[Eubacterium]_xylanophilum_group	2.85	0
Full-length vs Multiplex	Genus	Lachnospiraceae_NK4A136_group	5.56	0
Full-length vs Multiplex	Genus	[Eubacterium]_fissicatena_group	3.31	0
Full-length vs Multiplex	Genus	Acetatifactor	-1.70	1.55E-16
Full-length vs Multiplex	Genus	Blautia	0.96	0.00014162
Full-length vs Multiplex	Genus	Marvinbryantia	0.64	0
Full-length vs Multiplex	Genus	Lachnoclostridium	2.10	2.07E-22
Full-length vs Multiplex	Genus	Lachnospiraceae_UCG-006	3.28	0
Full-length vs Multiplex	Genus	Roseburia	2.46	2.56E-20
Full-length vs Multiplex	Genus	A2	0.14	0
Full-length vs Multiplex	Genus	Lachnospiraceae_UCG-001	3.36	0
Full-length vs Multiplex	Genus	Tepidibacter	-10.55	0
Full-length vs Multiplex	Genus	Bacillus	-8.91	0
Full-length vs Multiplex	Genus	Zymomonas	-4.33	0
Full-length vs Multiplex	Genus	Pigmentiphaga	-7.59	0
Full-length vs Multiplex	Genus	Serratia	-3.58	0
Full-length vs Multiplex	Genus	Libanicoccus	-3.52	0
Full-length vs Multiplex	Genus	Barnesiella	-0.94	0
Full-length vs Multiplex	Genus	Amphiplicatus	-8.92	0

Full-length vs Multiplex	Genus	UCG-003	-0.93	0
Full-length vs Multiplex	Genus	Olsenella	0.15	0.59050629
Full-length vs Multiplex	Genus	Parabacteroides	0.75	0
Full-length vs Multiplex	Species	Streptococcus_danieliae	-0.14	0
Full-length vs Multiplex	Species	Lactobacillus_murinus	4.05	0
Full-length vs Multiplex	Species	Lactobacillus_reuteri	4.04	0
Full-length vs Multiplex	Species	Bacteroides_acidifaciens	3.88	0
Full-length vs Multiplex	Species	Bacteroides_caecimuris	2.00	0
Full-length vs Multiplex	Species	Adlercreutzia_mucosicola	-0.63	0.00089084
Full-length vs Multiplex	Species	Akkermansia_muciniphila	5.70	0
Full-length vs Multiplex	Species	Burkholderiales_bacterium	4.27	0
Full-length vs Multiplex	Species	Alistipes_finegoldii	-3.69	0
Full-length vs Multiplex	Species	Lactobacillus_vaginalis	-9.14	0
Full-length vs Multiplex	Species	Muribaculum_intestinale	-6.34	5.94E-271
Full-length vs Multiplex	Species	Mucispirillum_schaedleri	-2.60	1.02E-22
Full-length vs Multiplex	Species	Zymomonas_mobilis	-4.55	0
Full-length vs Multiplex	Species	Alistipes_putredinis	-1.50	0
Full-length vs Multiplex	Species	[Clostridium]_leptum_1	-4.99	0
Full-length vs Multiplex	Species	Adlercreutzia_muris	-1.18	0
Full-length vs Multiplex	Species	Staphylococcus_carnosus	-1.28	0
Full-length vs Multiplex	Species	Acetatifactor_muris	-2.06	0
Full-length vs Multiplex	Species	Parasutterella_excrementihominis	-1.79	0
Full-length vs Multiplex	Species	Alistipes_onderdonkii	-1.18	0
Full-length vs Multiplex	Species	Lactobacillus_johnsonii	5.70	0
Full-length vs Multiplex	Species	Bifidobacterium_pseudolongum	4.16	0
Full-length vs Multiplex	Species	[Clostridium]_leptum_3	1.35	0
Full-length vs Multiplex	Species	Parabacteroides_goldsteinii	0.53	0
