# Supplementary figures and images for: Amplicon-Based Microbiome Profiling: From Second- to Third-Generation Sequencing for Higher Taxonomic Resolution
Source: Genes (Basel). 2023 Jul 31;14(8):1567. doi: 10.3390/genes14081567 (PMC10454624; doi:10.3390/genes14081567)

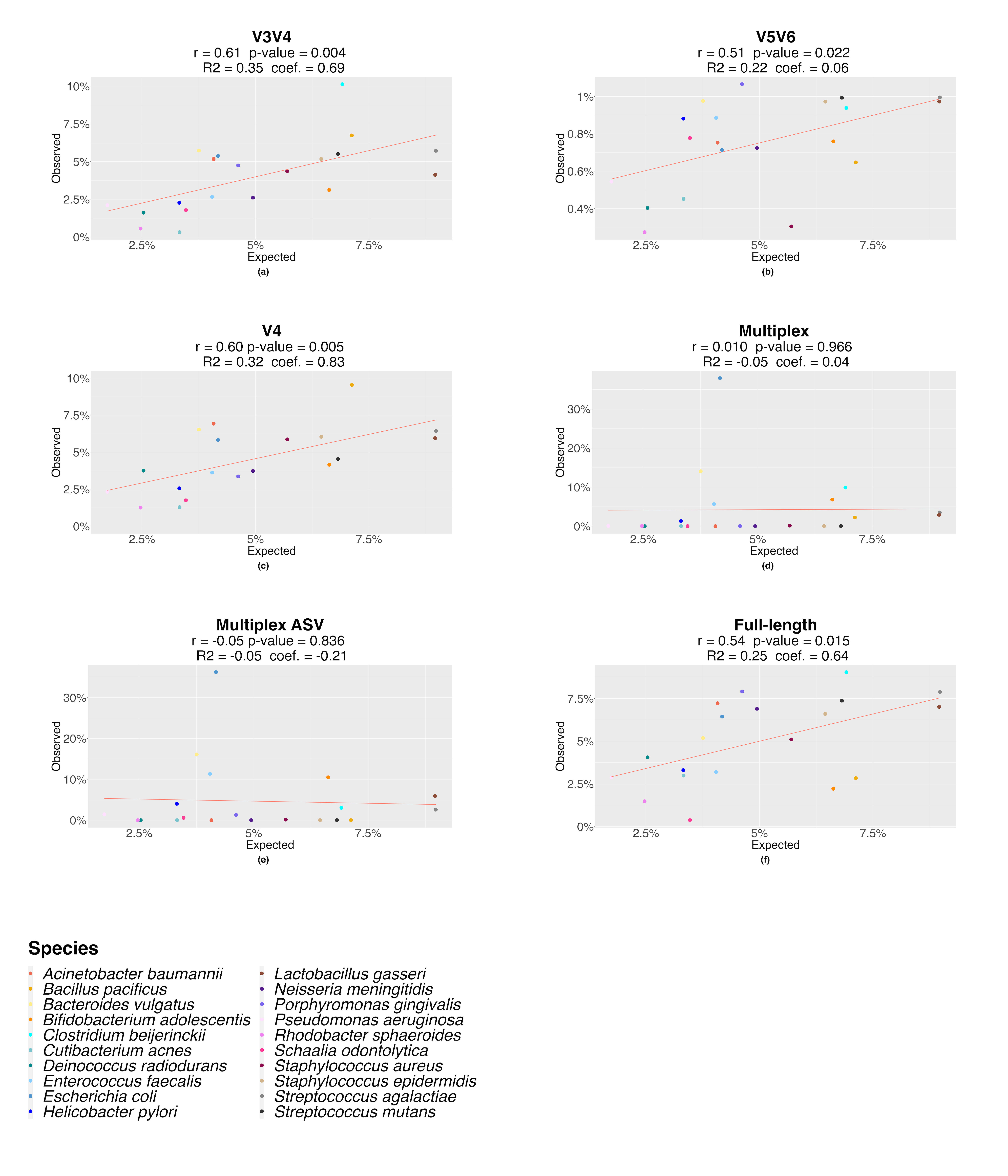

Supplement: Supplementary file 1 [file genes-14-01567-s001.zip › Figure S1.jpeg]

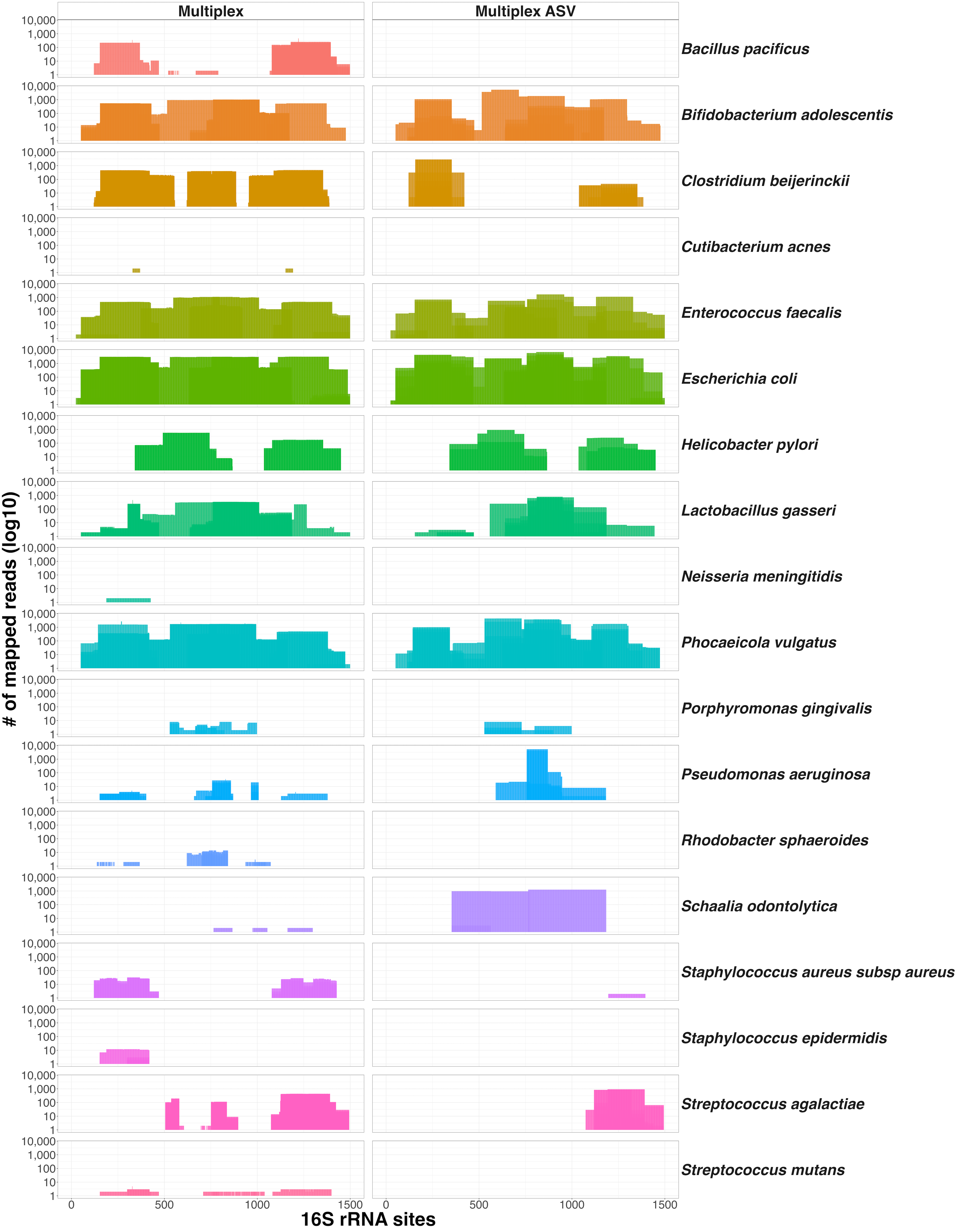

Supplement: Supplementary file 1 [file genes-14-01567-s001.zip › Figure S2.png]

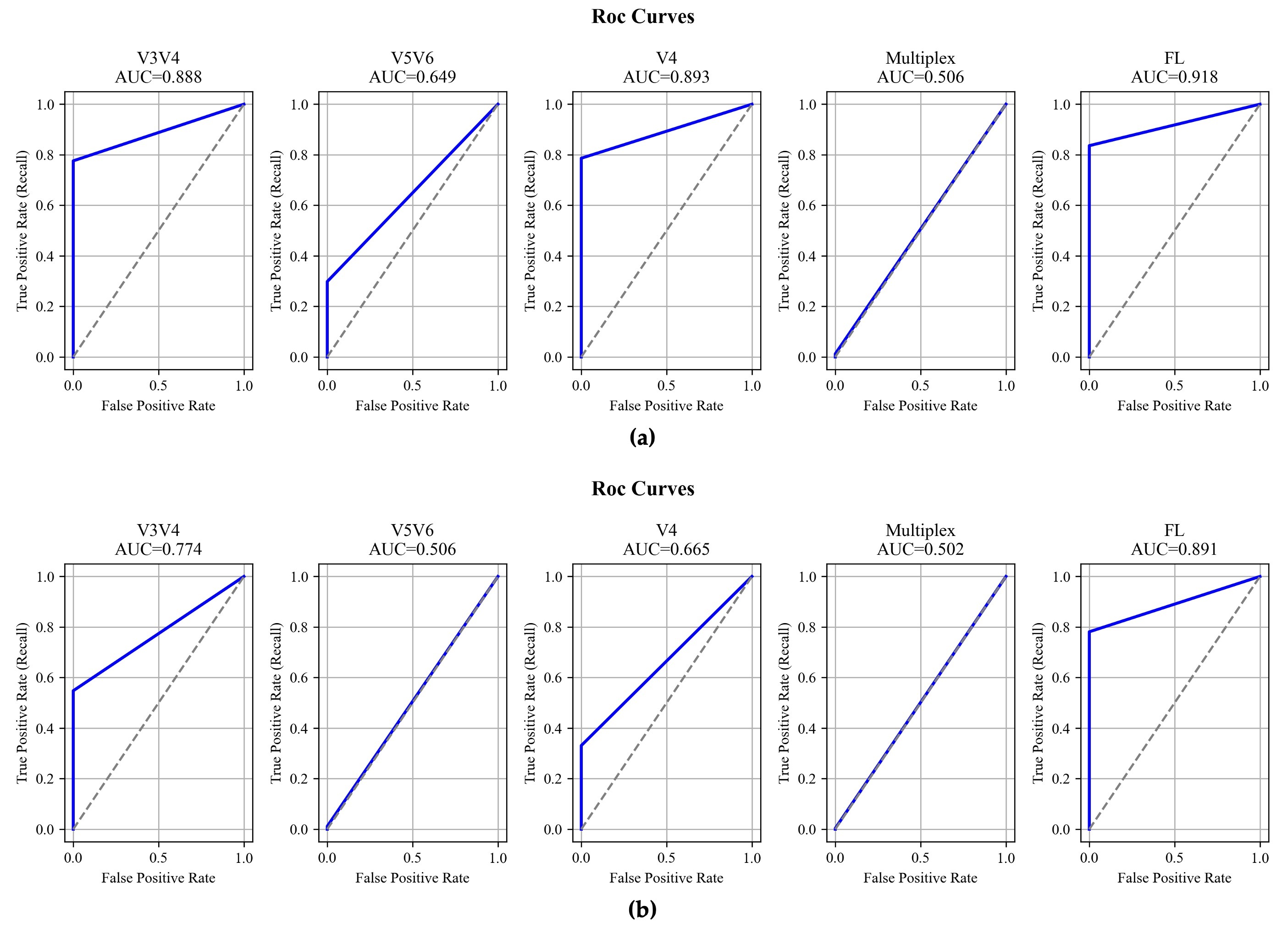

Supplement: Supplementary file 1 [file genes-14-01567-s001.zip › Figure S3.jpg]
